# Conformational Differences between Open and Closed States of the Eukaryotic Translation Initiation Complex

**DOI:** 10.1016/j.molcel.2015.06.033

**Published:** 2015-08-06

**Authors:** Jose L. Llácer, Tanweer Hussain, Laura Marler, Colin Echeverría Aitken, Anil Thakur, Jon R. Lorsch, Alan G. Hinnebusch, V. Ramakrishnan

**Affiliations:** 1 MRC Laboratory of Molecular Biology, Cambridge CB2 0QH, UK; 2 Laboratory of Gene Regulation and Development, Eunice K. Shriver National Institute of Child Health and Human Development, National Institutes of Health, Bethesda, MD 20892, USA; 3 Laboratory on the Mechanism and Regulation of Protein Synthesis, Eunice K. Shriver National Institute of Child Health and Human Development, National Institutes of Health, Bethesda, MD 20892, USA

## Abstract

Translation initiation in eukaryotes begins with the formation of a pre-initiation complex (PIC) containing the 40S ribosomal subunit, eIF1, eIF1A, eIF3, ternary complex (eIF2-GTP-Met-tRNA_i_), and eIF5. The PIC, in an open conformation, attaches to the 5′ end of the mRNA and scans to locate the start codon, whereupon it closes to arrest scanning. We present single particle cryo-electron microscopy (cryo-EM) reconstructions of 48S PICs from yeast in these open and closed states, at 6.0 Å and 4.9 Å, respectively. These reconstructions show eIF2β as well as a configuration of eIF3 that appears to encircle the 40S, occupying part of the subunit interface. Comparison of the complexes reveals a large conformational change in the 40S head from an open mRNA latch conformation to a closed one that constricts the mRNA entry channel and narrows the P site to enclose tRNA_i_, thus elucidating key events in start codon recognition.

## Highlights

• Structures of eukaryotic translation initiation complexes in open and closed states
• In the open complex the 40S head moves upward to open the mRNA entry channel latch
• Transition to closed state locks initiator tRNA in the P site base-paired with AUG
• The structures show how eIF3 contacts eIF2 and eIF1 on the 40S subunit interface

## Graphical Abstract

**Figure:**
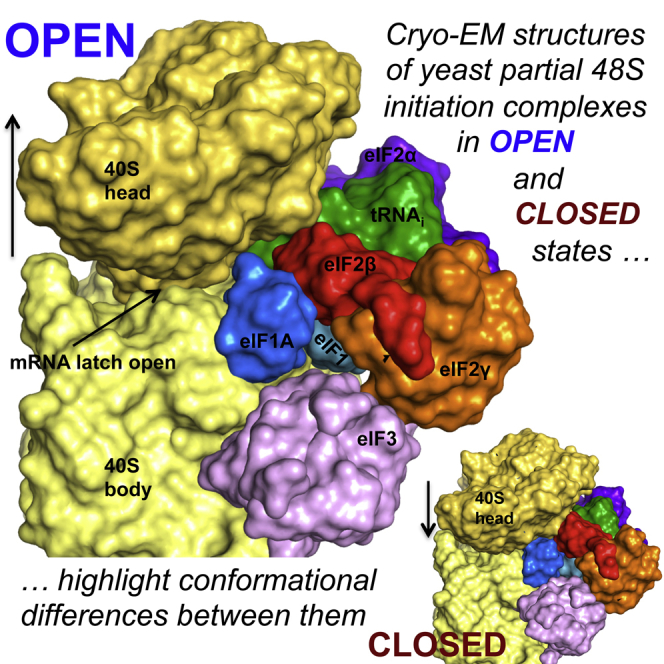

## Teaser

The small ribosomal subunit latch has to open to allow mRNA loading. Llacer et al. report cryo-EM reconstructions of yeast translation pre-initiation complexes in both open and closed conformations, highlighting structural differences and elucidating key events in start codon recognition.

## Introduction

In the first stage of eukaryotic translation initiation, the 40S ribosomal subunit and translation initiation factors eIF1, eIF1A, and eIF3 form a complex that facilitates loading of methionyl initiator tRNA (tRNA_i_) onto the 40S subunit as a ternary complex (TC) with eIF2-GTP. eIF5, a GTPase activating protein (GAP) for eIF2, is thought to bind to this complex along with TC. The 43S preinitiation complex (PIC) thus formed binds to the capped 5′ end of mRNA in collaboration with the eIF4F complex, which consists of the cap-binding protein eIF4E, scaffolding protein eIF4G, and RNA helicase eIF4A. This 48S PIC, in an open conformation with tRNA_i_ not fully engaged in the P site (P_OUT_), then scans along mRNA. During the scanning process, GTP bound to eIF2 is hydrolyzed but the dissociated phosphate (P_i_) is not released. Recognition of the start codon in the P site precipitates transition to a scanning-arrested, closed PIC with tRNA_i_ accommodated in the P site (P_IN_). This rearrangement triggers release of eIF1 and resultant dissociation of P_i_ (Hinnebusch and Lorsch, 2012; Jackson et al., 2010; Voigts-Hoffmann et al., 2012; Hinnebusch, 2014).

One consequence of this process is that the mRNA cannot be threaded into the 40S subunit, because eIF4F is bound to the 5′ end, and hence must be loaded laterally into the mRNA channel. In the empty 40S subunit, this channel is closed because interactions between helix h34 in the head and h18 in the body form a latch (Passmore et al., 2007) that must open to allow initial loading of mRNA on the 40S subunit. It is thought that eIF1 and eIF1A promote the formation of an open conformation of the 40S subunit conducive to scanning (Pestova et al., 1998; Pestova and Kolupaeva, 2002). Moreover, a low-resolution cryo-EM reconstruction (22 Å) of a 40S•eIF1•eIF1A complex suggested that eIF1 and eIF1A unlock the latch, but the factors themselves could not be seen in this structure (Passmore et al., 2007). In contrast, all recent structures, including those with eIF1 and eIF1A, show the latch in a closed conformation (Ben-Shem et al., 2011; Rabl et al., 2011; Weisser et al., 2013; Hashem et al., 2013; Lomakin and Steitz, 2013; Hussain et al., 2014). A complete structure of the open conformation, in which the positions of the various factors are visible, would shed light on the mechanism of initial mRNA loading, scanning, and start codon recognition, as well as the roles played by each factor in these events.

Apart from capturing only the closed-latch conformation, previous structures are also incomplete in other ways. A recent 48S complex (py48S) from our groups shows details of the interaction of eIF1 and eIF1A with the ribosome and tRNA_i_ during start-codon recognition, but it lacks density for most of the β subunit of eIF2 and all of eIF3 (Hussain et al., 2014). eIF3 is the largest and most complex of the eIFs, and is involved in nearly every aspect of initiation (Hinnebusch, 2006; Valásek, 2012). Mammalian eIF3 consists of 13 subunits and has a molecular weight of ∼800 kDa, whereas eIF3 in *Saccharomyces cerevisiae* and closely related yeasts is a ∼395 kDa complex of five essential subunits (eIF3a, eIF3b, eIF3c, eIF3g, and eIF3i) and a sixth non-essential and substoichiometric subunit, eIF3j (Valásek et al., 2002; Hinnebusch, 2006). Crystal structures of parts of the various subunits of eIF3 from yeast have recently been solved (Hinnebusch, 2014; Erzberger et al., 2014), and a cryo-EM structure of 40S•eIF1•eIF1A•eIF3 is now available (Aylett et al., 2015). Domains of eIF3 were also modeled in a moderate resolution cryo-EM reconstruction of a partial mammalian 43S complex (hereafter pm43S) that lacked eIF1, eIF1A, eIF5, and mRNA (Hashem et al., 2013). All known structures of eIF3 bound to the 40S, as well as crosslinking studies, indicate binding on the solvent surface of the subunit (Siridechadilok et al., 2005; Hashem et al., 2013; Erzberger et al., 2014; Aylett et al., 2015). And yet eIF3 has been shown to interact with eIF1, TC, and eIF5, all of which bind to the intersubunit surface of the 40S (Hinnebusch, 2014). How eIF3 interacts with components bound near the P site while itself binding to the opposite face of the 40S subunit remains a key question in understanding the mechanism of translation initiation.

Here we have addressed these questions by determining a cryo-EM reconstruction at 6.0 Å resolution of a partial yeast 48S complex (with mRNA containing a near-cognate AUC in lieu of an AUG codon) that reveals an open, scanning-competent state with tRNA_i_ not fully engaged in the P site, hereafter referred to as “py48S-open.” A second complex elucidated at 4.9 Å resolution contains mRNA with an AUG codon that presents a closed conformation with the latch closed, entry channel constricted, and tRNA_i_ locked into the P_IN_ state, hereafter referred to as “py48S-closed.” These structures show clear density for eIF1, eIF1A, mRNA, the entire TC, including previously unseen eIF2β, as well as segments of eIF3. The py48S-open complex shows an open latch conformation, expanded entry channel and widened P site, suggesting mechanisms for loading and scanning of mRNA and is markedly different from a pm48S reconstructed at 11.6 Å (Hashem et al., 2013). Comparison with py48S-closed illuminates the structural changes that occur within the 40S subunit, TC, and other eIFs during the transition from the open to closed state of the PIC that should arrest scanning and lock tRNA_i_ into the P site and highlights the importance of the 40S head conformation and roles of eIFs in stabilizing the two states. We also observe portions of eIF3 on the subunit-joining interface of the 40S subunit in both complexes, showing how eIF3 can contact TC and eIF1 near the P site while remaining bound to the solvent face of the 40S subunit.

## Results

### Formation and Overview of Structures

Yeast py48S-closed was assembled as described previously (Hussain et al., 2014) using an unstructured, uncapped mRNA with an AUG codon; 40S subunits from yeast *Kluyveromyces lactis*; and factors eIF1, eIF1A, eIF3, eIF5, and TC from *S. cerevisiae*. We used the U31:A39 variant of tRNA_i_ that stabilizes the P_IN_ state (Dong et al., 2014) to promote formation of the 48S in the P_IN_ state, but wild-type eIF2 rather than the Sui3-2 variant of eIF2 (Donahue et al., 1988) used previously (Hussain et al., 2014). We similarly combined 40S with eIF1, eIF1A, eIF3, TC, and mRNA to generate the py48S-open complex. However, to shift the equilibrium from the P_IN_ state, wild-type tRNA_i_ was used, the AUG codon was replaced with near-cognate AUC, eIF5 was omitted as it shifts the equilibrium toward P_IN_ state (Maag et al., 2006; Nanda et al., 2013), and recombinant eIF3 expressed in *E. coli* (and thus free of eIF5) was used instead of native eIF3 (see Supplemental Experimental Procedures).

**Figure 1:**
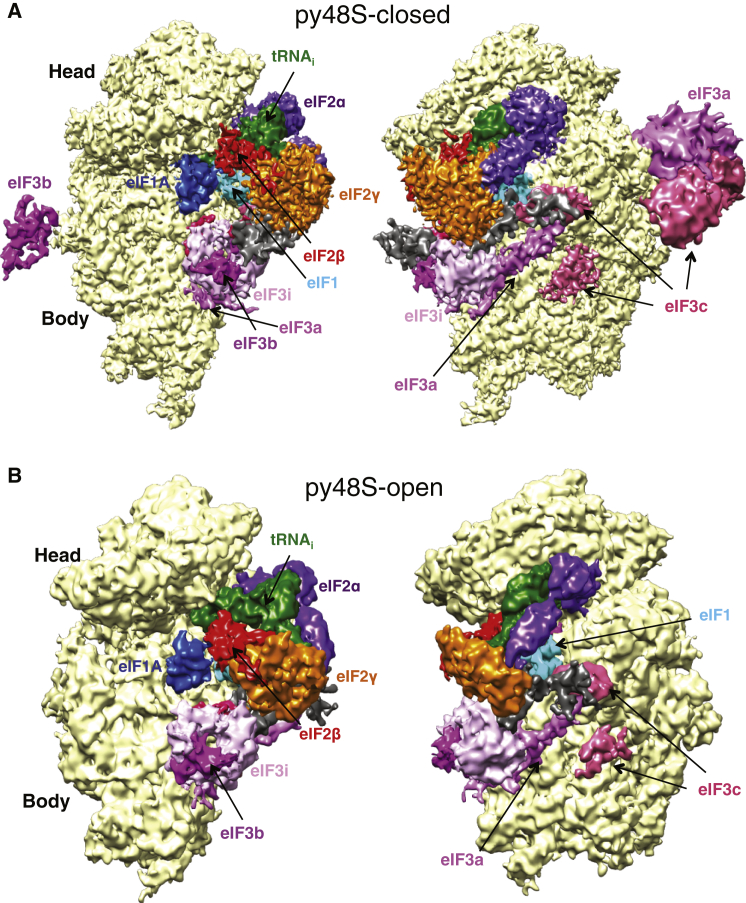
Cryo-EM Maps of Eukaryotic 48S PICs (A) Two views of py48S-closed. (B) Two views of py48S-open. Density for eIF3 is Gaussian-filtered. Unassigned density is in dark gray. See also Figures S1–S4; Table S1; and Movies S1 and S2.

The structures for py48S-closed and py48S-open were determined to overall resolutions of 4.9 Å and 6.0 Å, respectively (Figures 1, S1, and S2; Table 1; Movies S1 and S2). Like py48S (Hussain et al., 2014) the local resolution and density is best for the core of 40S and ligands directly attached to it (Figure S3 and Table S1). Large data sets and extensive 3D classification were required to obtain PIC classes with eIF1, eIF1A, eIF3, TC (including eIF2β), and mRNA all bound (Figure S1; see Supplemental Experimental Procedures). These classes comprised 1.8% of the total for py48S-closed and 1.0% for py48S-open. The majority of particles were deficient in one or more factors as a result of the characteristic dissociation of factors on the EM grid (Figure S1; See Supplemental Experimental Procedures).

We observe density in both complexes for subunits of eIF3, as well as for eIF1, eIF1A, mRNA, and all three subunits of TC. Interestingly, density for components of eIF3 appears in the subunit-joining interface, where it interacts with other eIFs. In the solvent interface, the density for eIF3 density is weaker, especially for py48S-open (discussed later). We observe density for eIF2β in both complexes (Figures 1 and S2). The py48S-closed (4.9 Å) is globally similar to our previously reported py48S (4.0 Å) (Hussain et al., 2014) (RMSD of 0.86 Å for 33,178 atoms of 18S) except for the additional densities for eIF2β and eIF3. The use of large data sets may have allowed us to isolate a class that includes eIF2β and eIF3, which would have been missed earlier in py48S. However, it is also possible that the use of WT eIF2 instead of Sui3-2 variant may have resulted in observation of these eIFs.

The large data set and extensive classification also enabled us to determine structures of a 40S•eIF1•eIF1A complex at 3.5 Å resolution (Figures S1 and S4A) and a 40S•eIF1•eIF1A•TC complex, representing a partial 43S PIC (py43S), at 15 Å resolution (Figures S1 and S4B), which has a conformation similar to pm43S (Hashem et al., 2013).

### Altered Conformation of the 40S Head in the Open Conformation of py48S

**Figure 2:**
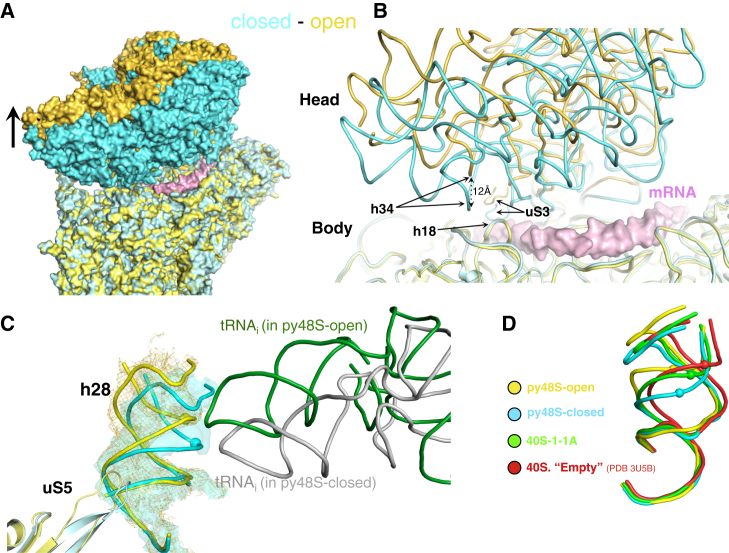
Distinct Position of the 40S Head Widens the mRNA Entry Channel and Opens the Latch in the Open Complex (A) Front view of superposition of py48S-open (yellow) and py48S-closed (cyan), showing mRNA (pink) from py48S (Hussain et al., 2014) to highlight the complete mRNA channel. (B) Superposition of refined models of py48S-open (yellow) and py48S-closed (cyan), indicating elements forming the latch. (C) h28 in py48S-open (yellow) and py48S-closed (cyan) based on superposition of the two complexes, viewed from the A site. tRNA_i_ and uS5 for the two complexes are also shown. (D) h28 in empty 40S (red, PDB: 3U5B), 40S•eIF1•eIF1A (green), py48-open (yellow), and py48S-closed (cyan) complexes. Equivalent atoms in h28 are shown as spheres. See also Figures S4 and S2 and Movies S3 and S4.

Whereas the orientations of the 40S body are similar in both py48S-open and py48S-closed, the two structures differ in the conformation of the 40S head (Figure 2; Movie S3). While the orientation of the head in py48S-closed is similar to that in py48S reported earlier (Hussain et al., 2014), in py48S-open, there is a remarkable upward movement of the head away from the body (Figures 2A and 2B), in a different direction from that distinguishing the 40S•eIF1•eIF1A complex from empty 40S or py48S (Movie S4). This head movement from py48S-closed to py48S-open is accompanied by a 7–8 Å change in the pitch of h28 (Figures 2C and 2D) and a repositioning of the β-hairpin of uS5 that contacts h28 (Figure 2C). This helix constitutes the “neck” of the 40S that connects the head to the body, and is compressed in py48S-closed but relaxed in py48S-open. Interestingly, mutations in this region of h28 (A1151, A1152, and U1627; *S. cerevisiae* numbering throughout the manuscript) lead to a Gcd^−^ phenotype, indicating less stable TC binding to the PIC (Dong et al., 2008).

This conformation of the head in py48S-open throws open the mRNA channel latch and widens the channel, particularly at the entry channel side near the A site (Figure 2B). Helix 34 and associated elements move away from h18 to open the latch. The py48S-open structure reveals both the upward shift of the 40S head and open-latch conformation, thus providing insights into changes involved during key steps of initiation.

The 40S head is also moved upward in py48S-open compared to pm43S (Hashem et al., 2013) (Figure S4C). In contrast, the head conformations of the pm43S and py48S-closed are more similar (Figure S4D). Note that pm43S lacks mRNA, and densities for eIF1, eIF1A, and eIF2β were missing in the reconstruction. In py43S (which most closely mimics pm43S), the latch is again closed and the head orientation is almost identical to that of pm43S (Figure S4E). The positions of TC in pm43S and py43S are also similar (Figure S4E); however, eIF1 and eIF1A densities are clearly seen in our py43S map (Figure S4B). Thus, the presence of TC with eIF3 in pm43S, or TC with eIF1 and eIF1A in py43S here, both lead to a similar orientation of the head with a closed latch and may thus represent a state prior to the binding of mRNA.

### A Widened P Site and Altered Orientation of tRNA_i_ in py48S-Open

**Figure 3:**
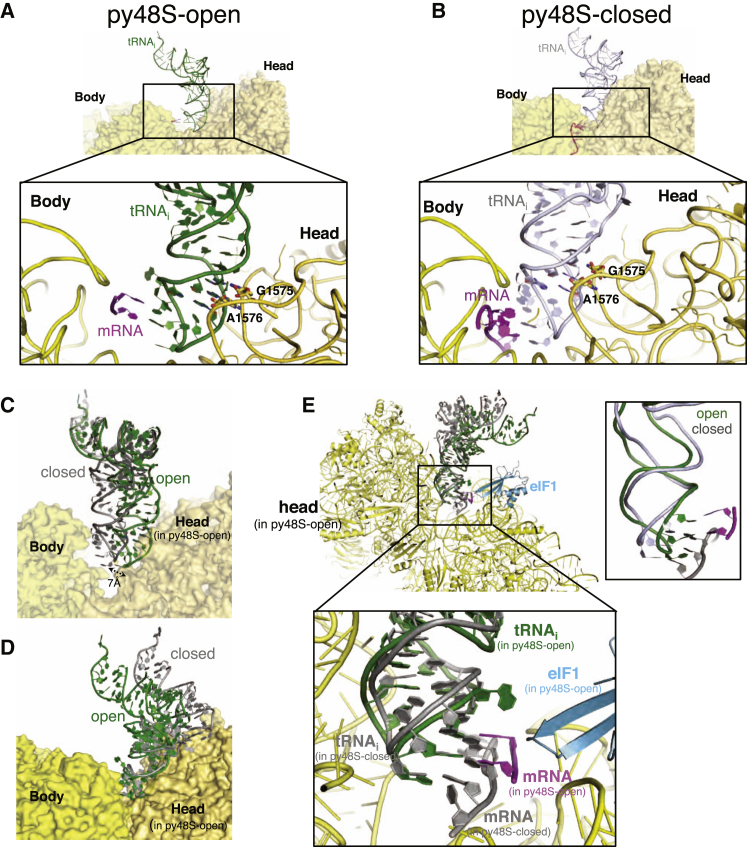
tRNA_i_ Is Not Engaged with P-Site Elements of the 40S Body in the Open Complex (A) tRNA_i_ in py48S-open, viewed from E site. The body and head of 40S are shown in lighter and darker shades of yellow. The zoomed view shows mRNA at the P site and recognition of conserved GC base pairs in ASL by rRNA bases. For clarity, 40S proteins and other factors are not shown. (B) The tRNA_i_ in py48S-closed viewed as in (A). (C) Superposition of the 40S body reveals distinct locations of tRNA_i_ in the P site of py48S-open (green) and py48S-closed (gray). The body and head of py48S-open complex are shown. The two ASLs are separated by about 7 Å in the P site. (D) Superposition of the 40S head of py48S-open (green) and py48S-closed (gray). (E) Superposition of two complexes as in (D), viewed from the A site. The mRNA of py48S-closed is in gray. Inset shows the superposition of the two positions of tRNA_i_ and interacting mRNA codon. See also Figure S5.

Interestingly, the P site in py48S-open is widened compared to that of py48S-closed (Figures 3A and 3B), lacking interactions between tRNA_i_ and the 40S body that occur in the closed complex (Figures 3A and 3B). As a result of the altered head position in py48S-open, the tRNA_i_ adopts a previously unobserved modified P/I orientation, which we call sP/I for “scanning P/I” conformation (Figure S5A; Movie S3). Nevertheless, the tRNA_i_ maintains the same contacts with the head in both complexes, which ensures that the conserved GC base pairs in the anticodon stem-loop (ASL) of tRNA_i_ are recognized by rRNA residues G1575 and A1576 in both py48S-closed and py48S-open (Figures 3A and 3B).

While the tip of the ASL of tRNA_i_ is deep within the P site in both the open and closed complexes, it is displaced laterally from the body by ∼7 Å in the py48S-open (Figure 3C), owing to both the widened P site and altered head orientation. Superimposing the head in the open and closed structures shows that the positions of the ASL relative to the head are very similar in both (Figures 3D and 3E); the ASL essentially moves with the head during the open-to-closed transition. In contrast, in pm43S (Hashem et al., 2013), the tip of the ASL is not deep in the P site (Hussain et al., 2014) and thus does not track with the head movement.

Although the ASL tracks with the head as it moves from the open to closed conformation, the positions of the tRNA_i_ acceptor arm in both structures are superimposable relative to the body (Figure 3C). As the ASL remains in contact with the head, it must bend in going from the open to closed state, which allows the tRNA_i_ to maintain codon-anticodon interaction during the transition that would otherwise clash with the 40S body (Figure S5B and Movie S3). The bent ASL in py48S-close is similar to that observed earlier in py48S (Hussain et al., 2014) and pm48S (Lomakin and Steitz, 2013), and allows base pairing with the codon in P_IN_ state.

### Path of mRNA in the Two Structures

In our previous py48S complex (Hussain et al., 2014), mRNA was observed throughout the mRNA channel. In contrast, the py48S-closed here shows density for mRNA mainly in the exit channel (Figure S2A). Strikingly, in py48S-open, the mRNA entry channel is widened, which, along with the open latch, produces a conformation that should allow single-stranded mRNA to be slotted directly into the mRNA-binding channel (Figures 2A and 2B). We observe discontinuous densities for mRNA, mainly in the P and E sites of py48S-open, including density consistent with base pairing between the A and U of the AUC codon and the U and A of the anticodon (Figure S2B, right). Incidentally, the mRNA also has another AUC codon, but because it is only 3 nt away from 5′ end, it appears not to be involved in recognition. The P-site codon has moved in concert with the ASL and 40S head such that base pairing is maintained in py48S-open, despite the tRNA_i_ not yet having been fully accommodated in the eP/I′ configuration (Figures 3E and S5B). The minimal density for mRNA in py48S-open suggests that, probably as a result of the widened mRNA channel, the mRNA has minimal contact with the ribosome apart from base pairing with the anticodon, which should facilitate scanning. Upon AUG recognition, head repositioning stabilized by interaction of the N-terminal tail (NTT) of eIF1A with the codon:anticodon duplex (discussed below), and latch closure, will narrow the entry channel to fix the mRNA and arrest scanning.

### Changes in eIF1and eIF1A between the Closed and Open States of py48S

**Figure 4:**
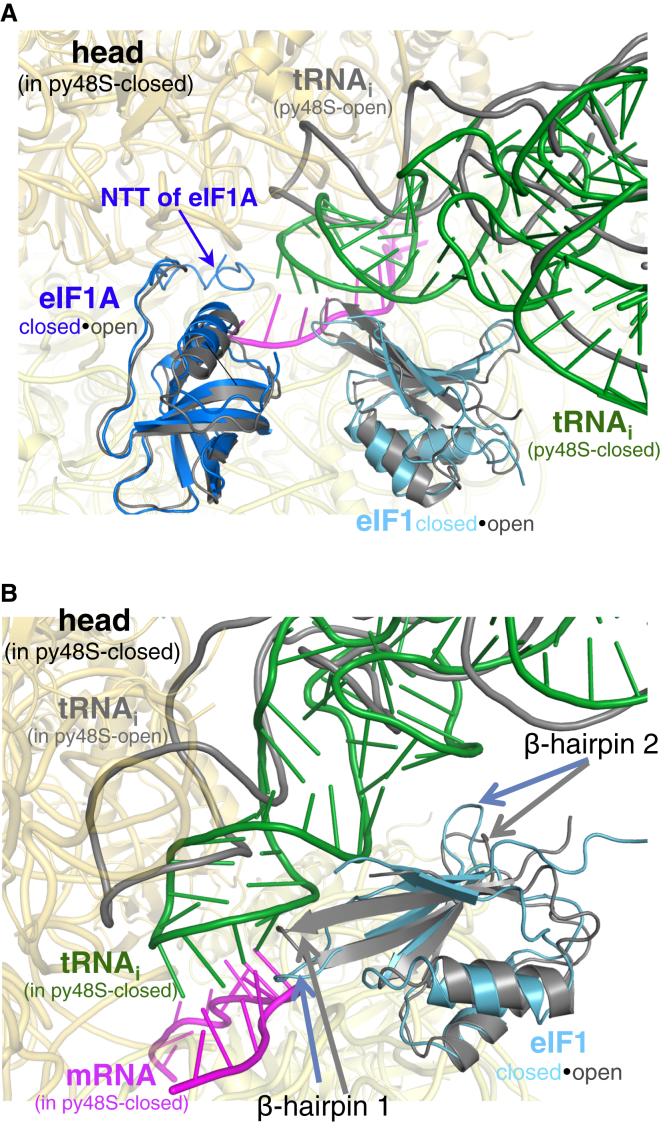
Contacts of eIF1A-NTT and eIF1 with tRNA_i_ Restricted to the Closed Complex (A) Superposition of the open and closed complexes with the ligands of py48S-closed shown in color while those of py48S-open are in gray. Only the 40S of py48S-closed is shown (yellow). The zoomed view shows the NTT of eIF1A in the two complexes. (B) Superposition of the open and closed complexes as in (A).

We observe eIF1and eIF1A clearly in both the open and closed PICs (Figures 1, S2A, and S2B), but with marked changes in their conformations between the two states. The overall conformation of eIF1A is similar in the two complexes, but the NTT of eIF1A interacts with the anticodon-codon duplex only in py48S-closed, as in our previous py48S without eIF3 (Hussain et al., 2014)—consistent with its role in promoting recognition of a cognate start codon (Figure 4A). In contrast, the NTT is disordered in py48S-open (Figure 4A), and in the 40S•eIF1•eIF1A complex (Figure S4A), as in all other reported PICs (Weisser et al., 2013; Lomakin and Steitz, 2013; Hussain et al., 2014). There is no distinct density for the C-terminal tail (CTT) of eIF1A in any of the closed complexes as expected from previous hydroxyl radical cleavage studies that show that eIF1A-CTT interferes with the P-site tRNA (Yu et al., 2009; Nanda et al., 2013). The CTT of eIF1A is also not modeled in py48S-open because of lack of clear unambiguous density.

The overall position of eIF1 in the two complexes is also similar (Figure 4B), but β-hairpins 1 and 2 of eIF1 are positioned differently. Their orientations in py48S-open resemble those observed in the 40S•eIF1•eIF1A complex, with no steric clash with tRNA_i_. In py48S-closed, however, the two β-hairpins are displaced to avoid a clash with the now-accommodated tRNA_i_ (Figure 4B), as seen earlier in py48S (Hussain et al., 2014). An interesting feature in py48S-open is the interaction of β-hairpin 1 with the AUC codon in the P site (Figure 3E). As we observe a similar interaction with the AUG codon in py48S-closed (Figure S5B), the conformation of β-hairpin 1 changes between the two states to follow the tRNA_i_ ASL and P-site codon as they are adjusted during the open-to-closed transition, preserving this interaction (Figure 4B).

### eIF2β Links TC to the 40S Head and Body in the py48S-Open Complex

**Figure 5:**
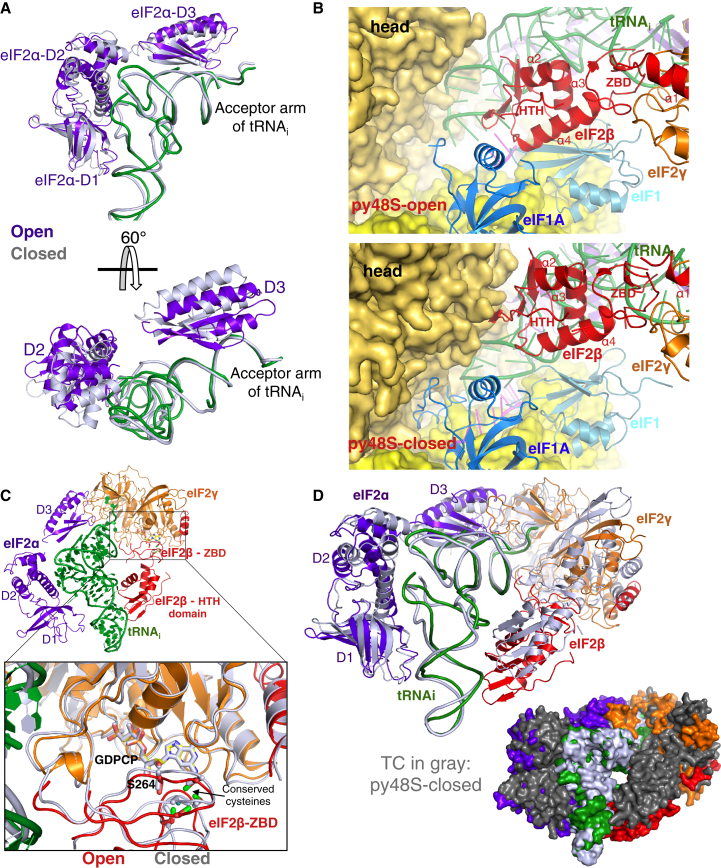
Distinctive Interactions of eIF2β with eIF1, eIF1A, and tRNA_i_ Occlude the mRNA Channel in py48S-Open (A) Conformational changes in eIF2α based on superposition of the TC coordinates using tRNA_i_ as the reference. The eIF2α and tRNA_i_ of py48S-open are shown in color and those of py48S-closed are in gray. (B) Position of eIF2β with respect to tRNA_i_, eIF1, eIF1A, and 40S head in py48S-open and py48S-closed. (C) Similar position for the ZBD of eIF2β in both complexes with respect to eIF2γ. The eIF2β, eIF2γ and tRNA_i_ of py48S-open are shown in color while those of py48S-closed are in gray. Ser264 is shown as sticks near conserved cysteines. (D) Cartoon and surface representations of the superimposition of TC coordinates in py48S-open (color) and closed (gray) complexes based on tRNA_i_ as reference. It shows the internal conformational change within TC during transition from the open to the closed conformation. While D2 and the helix connecting the D1 and the D2 domains of eIF2α experience an internal rearrangement, eIF2α-D3, eIF2γ and eIF2β rotate together around the acceptor arm of the tRNA. See also Figures S6 and S7 and Movie S5

We observe density for all three subunits of eIF2 in the PIC, including eIF2β (Figures S2A and S2B). eIF2 is bound primarily to the 40S head in py48S-closed (Figure 1A) and, as in py48S (Hussain et al., 2014), eIF2α-D1 (domain 1) and the tRNA_i_ ASL together attach TC to the head (Movie S3). During the transition from py48S-open to py48S-closed, eIF2α-D1 rotates slightly, thus avoiding a clash with the 40S body (Movie S3). This positions the loop containing Arg55 and Arg57 to enable their interactions with mRNA nucleotides −2 to −3 in the E site (Figures S6A and S6B), as observed in the py48S. Another consequence of this rotation is that eIF2α-D2 and the D-and T-arms of the tRNA_i_ are positioned closer to the head in py48S-closed compared to py48S-open (Figures 3A and 3B). Moreover, eIF2α-D2 moves in relation to eIF2α-D1 and interacts closely with the D- and T-loops of tRNA_i_ (Figure 5A; Movie S3). The third domain eIF2α-D3 moves with respect to the acceptor arm of the tRNA_i_ (Figures 5A; Movie S5).

We could model most of eIF2β in both complexes (Figures 1 and S2), except for the disordered N-terminal residues 1–125 and the last 20 C-terminal residues of the protein. As in previous archeal βγ complexes (Stolboushkina et al., 2008; Sokabe et al., 2006), eIF2β is tightly attached to eIF2γ by its N-terminal helix α1 (Figures S2A and S2B). Notably, in py48S-open, the helix-turn-helix (HTH) domain of eIF2β binds to eIF1 and eIF1A on the 40S body and to tRNA_i_ bound to the 40S head, bridging the 40S head and 40S body, without direct interactions with the 40S itself (Figure 5B, upper). These interactions likely stabilize the open conformation during scanning in the absence of a complete codon-anticodon duplex.

During rearrangement to the closed complex, the eIF2β HTH domain is positioned away from eIF1 and eIF1A (Figure 5B, lower) and binds to elements of the 40S head. Because of its altered position, the HTH domain also makes contacts with the tRNA_i_ that are distinct from those occurring in py48S-open (Figure 5B, upper). The position of eIF2β in py48S-open would result in a clash with both eIF1 and eIF1A in py48S-closed due to inward movement of the 40S head and body (Figure S6C).

The zinc-binding domain (ZBD) of eIF2β is positioned close to the GTP binding pocket of eIF2γ in both complexes (Figure 5C), similar to its position in some archaeal βγ complexes (Stolboushkina et al., 2008), although the ZBD itself was disordered in that structure. Because eIF1 is present in both complexes and eIF1 dissociation from the PIC is a prerequisite for P_i_ release from eIF2•GDP•P_i_ on AUG recognition (Algire et al., 2005), it remains unclear whether changes in the interaction of the eIF2β-ZBD with the eIF2γ GTPase center on eIF1 release are involved in P_i_ release.

In our previously reported py48S structure (Hussain et al., 2014), density for eIF2β was largely absent. This may be because we used the Sui3-2 variant of eIF2 harboring the S264Y substitution in eIF2β to stabilize the closed PIC conformation. Interestingly, S264Y maps to the interface between the eIF2β-ZBD and the eIF2γ GTPase center (Figure 5C). As such, it might destabilize this interface and increase mobility of the eIF2β-ZBD, which could disrupt the interactions of eIF2β with eIF1 and eIF1A that are unique to py48S-open (Figure 5B). This might explain how the Sui3-2 mutant stabilizes TC binding in the closed conformation (Martin-Marcos et al., 2014). Sui3-2 may specifically destabilize the open conformation and shift the equilibrium toward the closed conformation, thus accounting for its increased utilization of near-cognate start codons in vivo (Donahue et al., 1988).

Interestingly, a superposition of eIF2βγ using eIF2γ as a reference shows that the relative orientation of these two subunits is the same in both py48S-open and py48S-closed (Figure S6D). Hence, eIF2βγ, along with eIF2α-D3, alters its position relative to tRNA_i_ and domains 1 and 2 of eIF2α in a concerted manner between the two complexes (Figures 5D and S6E; Movie S5).

### Initiation Accuracy In Vivo Is Reduced by Disrupting eIF2β Contacts with tRNA_i_ or eIF1 that Occur Only in py48S-Open

**Figure 6:**
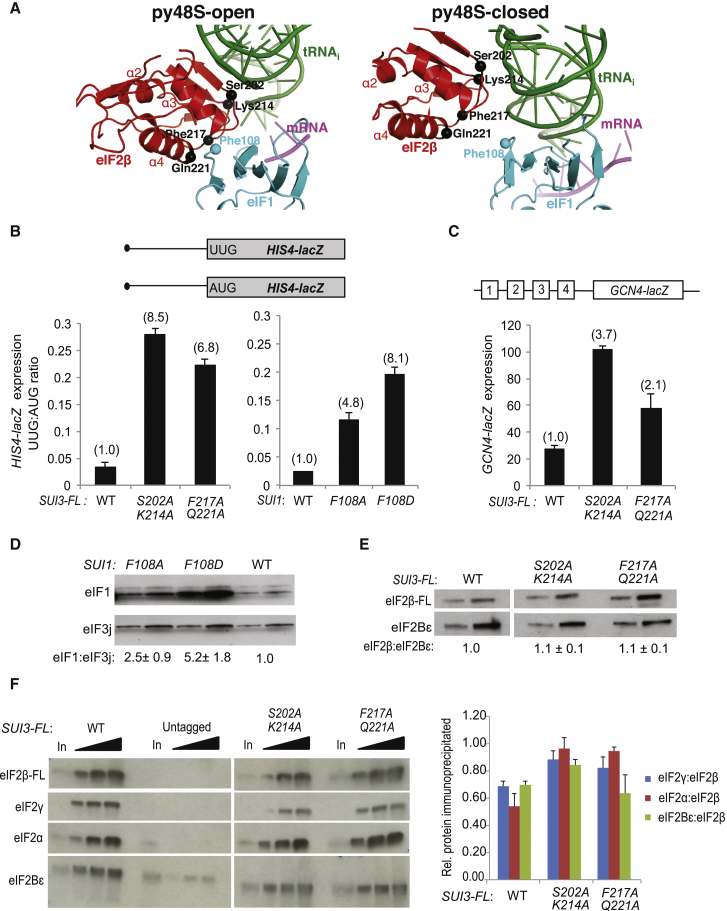
Genetic Evidence that eIF2β Interactions with the tRNA_i_ ASL and eIF1 Preferentially Stabilize py48S-Open to Impede Initiation at Near-Cognate UUG Codons In Vivo (A) Positions of eIF1, eIF2β, and tRNA_i_ in the py48S-open and py48S-closed, with residues substituted in genetic studies shown as spheres. (B) Expression of *HIS4-lacZ* reporters with AUG or UUG start codons in strains of the indicated *SUI3* or *SUI1* genotypes, expressed as mean (± SEM) ratios of UUG- to AUG-reporter expression with fold-changes relative to WT in parentheses. (C) Expression of the *GCN4-lacZ* reporter expressed as mean (± SEM) units of β-galactosidase. (D and E) Western analysis of eIF1 (D) or eIF2β (E) proteins in whole-cell extracts (WCEs), with eIF3j or eIF2Bε analyzed as loading controls, reported as mean (± SEM) eIF1:eIF3j ratios or eIF2β/eIF2Bε ratios, normalized to the WT ratios, determined from biological replicates. Lanes have been cropped from the same gels. (F) WCEs were immunoprecipitated with FLAG affinity resin and immune complexes subjected to western analysis to detect Flag-eIF2β and co-immunoprecipitated eIF2γ, eIF2α, and eIF2Bε, resolving 1×, 2×, or 3× amounts in successive lanes. In, 20% input WCEs. Western signals were quantified to yield mean (± SEM) recoveries of eIF2γ, eIF2α, or eIF2Bε normalized to Flag-eIF2β. Lanes have been cropped from the same gels. Error bars represent the SEM from three biological replicates.

The fact that eIF2β makes interactions with the tRNA_i_ ASL and eIF1 in py48S-open that are missing or altered in py48S-closed (Figure 6A) suggests that these contacts specifically stabilize the open, scanning conformation of the PIC. If so, then substituting residues at these contacts should increase the frequency of initiation at UUG codons by facilitating rearrangement from the open to closed conformations in the absence of a perfect start codon:anticodon duplex in the P site. Supporting this prediction, substitutions of eIF2β Phe217/Gln221 and eIF1 Phe108, residues juxtaposed at the eIF2β/eIF1 interface in py48S-open (Figure 6A), substantially increase the UUG:AUG expression ratio for matched *HIS4-lacZ* fusions differing only in the start codon (Figure 6B). The eIF1-F108A/F108D substitutions also increase eIF1 abundance (Figure 6D), an established indicator of relaxed discrimination against the suboptimal context of the AUG start codon of the (*SUI1*) mRNA encoding eIF1 (Martin-Marcos et al., 2011). Thus, eIF1-F108A/F108D facilitate initiation for both a near-cognate start codon and an AUG in poor context. eIF2β substitutions S202A/K214A also increase UUG initiation (Figure 6B) and, consistent with impaired eIF2β interaction with tRNA_i_, derepress expression of a *GCN4-lacZ* reporter (Figure 6C), an in vivo indicator of reduced TC assembly or binding to the scanning PIC (Hinnebusch, 2005). None of the eIF2β substitutions significantly affect eIF2β abundance (Figure 6E) or its assembly with eIF2α/eIF2γ in the eIF2 complex (Figure 6F).

### Placement of eIF3 subunits on both faces of the PIC

**Figure 7:**
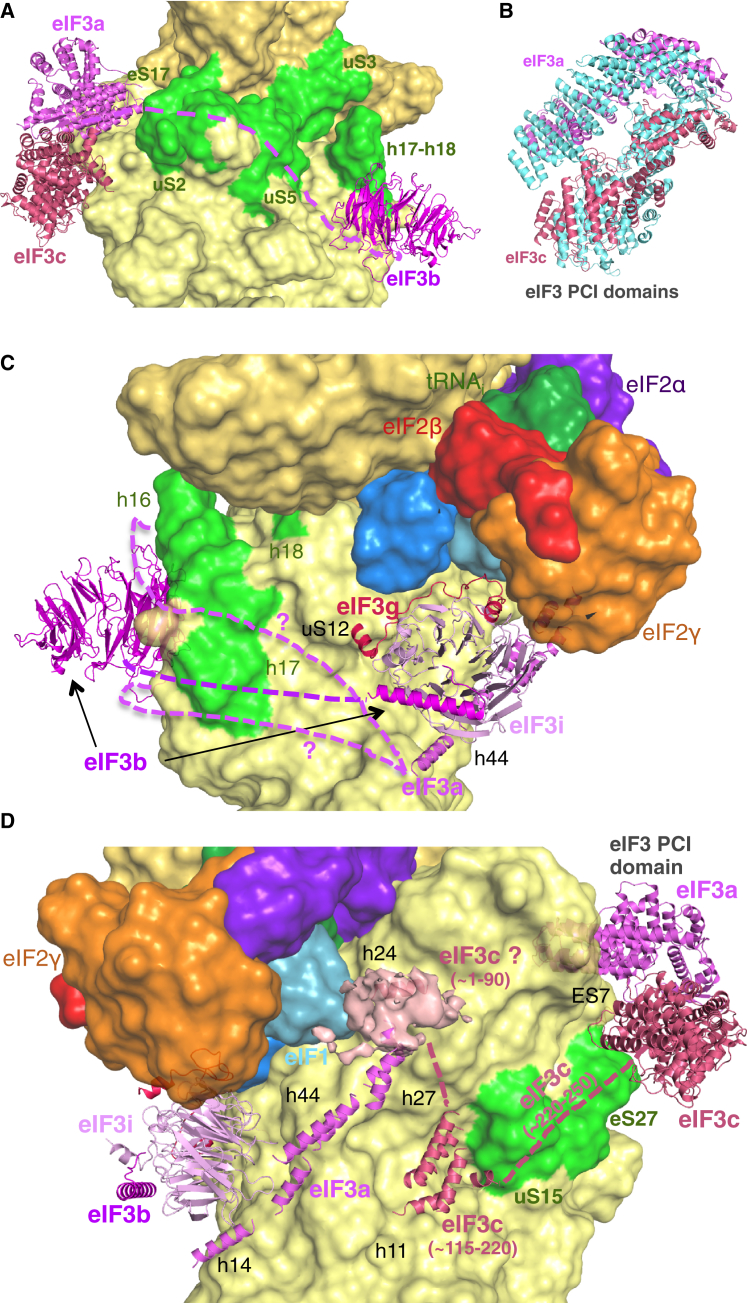
Structural Arrangement of eIF3 Components in 48S PICs (A) Locations of the eIF3a/eIF3c PCI domains and β-propeller of eIF3b at different positions on the solvent-exposed surface of the 40S, highlighting rRNA helices and ribosomal proteins (green) predicted to bind to eIF3a. The proposed path of the unassigned central portion of the eIF3a-CTD connecting the PCI domain to the subunit interface is shown as a dashed purple line. (B) Lateral displacement of eIF3a/eIF3c PCI domains in py48S-closed versus their positions in yeast 40S•eIF1•eIF1A•eIF3 (PDB: 4UER). (C) Trimeric eIF3b-CTD/eIF3i/eIF3g-NTD subcomplex is shown near h44 and interacting with eIF2γ and the 40S interface surface. The β-propeller of eIF3b is also shown. Two alternative proposed paths of the eIF3a-CTD connecting the PCI domain to the bundle of helices below the eIF3i β-propeller are shown as dashed purple lines. (D) A cluster of helices tentatively assigned to eIF3c is located near h11 and uS15 (green). A globular density with a single modeled helix is tentatively assigned to the eIF3c-NTD in proximity to eIF1 and h24. The proposed path of a linker connecting the cluster of helices to the eIF3c PCI domain is shown as a dashed magenta line. Long helices tentatively assigned to eIF3a bridge the eIF3i β-propeller and h44 with the putative eIF3c-NTD and h24.

We observe density for eIF3 in both py48S-open and py48S-closed (Figures 1A and 1B). At this resolution, we can identify helices, place domains of known structure into the density, and make tentative assignments of previously unobserved segments of eIF3, based on secondary structure predictions (Figures S2C, S2D, and S2E; Supplemental Experimental Procedures). Because the densities attributable to eIF3 on the subunit interface are similar in both complexes, we describe the appearance of eIF3 only in the higher resolution py48S-closed (Figure 7).

The two PCI domains of the eIF3a/eIF3c heterodimeric core bind near the left shoulder of the 40S solvent face (Figure 7A; Movie S1), as in the yeast 40S•eIF1•eIF1A•eIF3 structure (Aylett et al., 2015). However, in py48S-closed, the PCI domains are displaced laterally, which may reflect a conformational change in eIF3 during different steps of initiation (Figure 7B). We modeled the eIF3b β-propeller domain with the help of the 40S•eIF1•eIF1A•eIF3 structure (Figures 7A and 7C) as we detect only a part of it in the density (Figures 1A and 1B). In py48S-open, weak densities of the PCI domains and eIF3b β-propeller appear only in low-resolution filtered map contoured at lower threshold (not shown) and were not modeled. It is not clear whether the much weaker density for these regions of eIF3 in py48S-open is due to the lower quality of the data (fewer particles and lower resolution) or inherently greater flexibility or lower occupancy of these domains in py48S-open.

Remarkably, we see additional density for eIF3 in both complexes, at the subunit interface near h44, uS12, and eIF2γ (Figures 1A and 1B). Based on its characteristic shape and dimensions, we assigned this density to the trimeric subcomplex composed of the β-propeller domain of eIF3i, ∼30 residues from the eIF3b C-terminal domain (CTD) and ∼50 residues from the N-terminal domain (NTD) of eIF3g (Erzberger et al., 2014) (Figure S2D; Movie S1; See Supplemental Experimental Procedures). eIF3i is positioned in the vicinity of eIF2γ, and the NTD of eIF3g may directly contact eIF2γ in the py48S complexes (Figures 7C and 7D). eIF3i was earlier predicted to bind in two possible positions at the solvent-exposed surface of the 40S subunit, either above or below the β-propeller domain of eIF3b (Erzberger et al., 2014). Neither of these configurations is consistent with the density we observe, suggesting that eIF3 undergoes a significant rearrangement undetected by prior models, perhaps on binding mRNA. This position, in which eIF3i holds eIF3g against eIF2γ and by consequence promotes the intricate TC/eIF1A/eIF1 interaction network, might explain the suppression of eIF3i and eIF3g mutant phenotypes by overexpression of eIF1 or eIF1A and the formation of aberrant 43S complexes observed in the absence of these subunits (Cuchalová et al., 2010; Herrmannová et al., 2012). This configuration also places eIF3i and eIF3g along the path of mRNA through the decoding center, consistent with the scanning defects observed for mutants of these subunits (Cuchalová et al., 2010).

We also observe density in both complexes for a cluster of five α helices in a pocket formed by h11, h24, h27, h44, and uS15 that has been putatively assigned to a predicted helix-rich segment in the eIF3c-NTD (Figure 7D; See Supplemental Experimental Procedures), which we connect to the eIF3c PCI domain by a ∼30-residue flexible linker. The remaining N-terminal residues of eIF3c likely emanate from the five-helix cluster and mediate the known interaction of eIF3c-NTD with eIF1 (Fletcher et al., 1999; Asano et al., 2000), which appears to enhance the stability of eIF1 within the PIC (Valásek et al., 2004; Karásková et al., 2012). We therefore tentatively assigned the globular density in contact with eIF1 as the N-terminal region of the eIF3c-NTD (1–90 residues), with a single α-helix near h24 modeled in the density (Figure 7D; see Supplemental Experimental Procedures). This moiety approaches the surface of eIF1 identified as an eIF3c-binding surface (Reibarkh et al., 2008).

Closer to the subunit interface, we detect density in both complexes for an extended helical region spanning h14, h44, and h27 (Figures 1, S2E, and 7D). These helices have been provisionally assigned to a region in the CTD of eIF3a (See Supplemental Experimental Procedures) predicted to have long helices (Dong et al., 2013), and they bridge the β-propeller domain of eIF3i and the putative eIF3c-NTD moiety near eIF1. This assignment places the extreme C-terminal ∼100 residues of eIF3a not modeled here in the vicinity of the TC, consistent with a known eIF3a-CTD interaction with eIF2 (Valásek et al., 2002). We suggest that the unassigned central portion of eIF3a projects away from the eIF3a PCI domain near the exit channel on the 40S solvent face subunit and passes through the mRNA entry channel and across the intersubunit face, connecting with the extended helices assigned to the eIF3a-CTD (Figures 7A and 7C). As these extended helices approach the eIF3c-NTD (Figure 7D), it appears that eIF3a and eIF3c together encircle the PIC. This proposal is consistent with previous observations that regions of the eIF3a CTD interact with 40S components at the mRNA entry channel (Valásek et al., 2003; Chiu et al., 2010) as well as structural models for the yeast 40S•eIF1•eIF3 complex (Erzberger et al., 2014) and the pm43S (Hashem et al., 2013), and chemical and enzymatic footprinting data (Pisarev et al., 2008), all of which place eIF3 components at both the exit and entry channels on the solvent side of the 40S subunit.

## Discussion

Our py48S-open and py48S-closed structures contain density for eIF1, eIF1A, all three subunits of eIF2 bound to Met-tRNA_i_, mRNA, and various components of eIF3. Although both structures are at lower resolution than the previously reported py48S (Hussain et al., 2014), this is likely to be the result of the small fraction of the particles in these classes. Despite this, the presence of additional factors here results in an overall stabilization and better local resolution for eIF1, eIF2α, eIF2γ, and tRNA_i_ in py48S-closed (Table S1).

We observe eIF2β in py48S-open complex, where it connects eIF1 and eIF1A on the body with tRNA_i_ on the 40S head. These bridging interactions should stabilize both TC and eIF1 binding in the scanning PIC prior to achieving a perfect AUG:anticodon duplex in the P site. Being unique to py48S-open, the eIF2β contacts with eIF1 and tRNA_i_ should specifically stabilize the scanning complex. Consistent with this prediction, substitutions at both interfaces decreased the probability of continued scanning at near-cognate UUG start codons in yeast cells—presumably enabling rearrangement to the closed complex without a perfect start codon:anticodon duplex in the P site—thus establishing that eIF2β/eIF1 and eIF2β/tRNA_i_ contacts in py48S-open promote initiation accuracy in vivo. The network of eIF2β interactions with eIF1/eIF1A/tRNA_i_ should also impede mRNA insertion into the mRNA channel at the P site: eIF2β is likely repositioned to allow mRNA recruitment. Modeling either the conformation of eIF2β in py48S-closed, where it no longer contacts eIF1 and eIF1A (Figure S7A), or the distinct conformations observed in archaeal βγ complexes (Stolboushkina et al., 2008; Yatime et al., 2007) (Figure S6D), into py48S-open reveals unfettered access to the mRNA channel, supporting the notion that transient repositioning of eIF2β would allow mRNA recruitment and that eIF2β serves as a barrier to mRNA release during scanning.

These structures show how eIF3 can interact with TC and eIF1 close to the P site at the inter-subunit interface even while the majority of its contacts map to the remaining solvent-exposed surfaces of the 40S subunit. Based on our modeling, eIF3 appears to connect the entry and exit channels on the solvent face of the 40S subunit to the center of action at the P site. None of the core subunits of eIF3 has previously been observed at the subunit interface, except for eIF3j (Erzberger et al., 2014; Aylett et al., 2015), which was excluded from our study. We note however that our complexes contain mRNA, whereas all previous PICs with eIF3 lacked mRNA (Hashem et al., 2013; Erzberger et al., 2014; Aylett et al., 2015). It is likely that the position of eIF3i observed earlier (Erzberger et al., 2014; Aylett et al., 2015) may represent its position prior to mRNA binding and that the presence of mRNA in our complexes may have led to the previously unobserved conformation of eIF3 at the inter-subunit interface. Interestingly, the positioning of the eIF3b-CTD/eIF3i/eIF3g-NTD module observed here, in proximity to eIF2γ, might hinder the insertion of mRNA into the mRNA-binding channel of the ribosome, making it likely that this conformation exists only after mRNA loading and suggesting that it might lock mRNA into the scanning complex. We propose that eIF3 undergoes a substantial conformational change upon mRNA binding, relocating both the eIF3b-CTD/eIF3i/eIF3g-NTD module and portions of eIF3a and eIF3c to enable their interactions with eIF2 and eIF1 in the decoding center and thereby facilitate key steps in scanning and start codon recognition. This rearrangement may signal the presence of mRNA within the PIC to other eIFs, notably eIF2.

Integrating the complete array of structures described in this report allows us to propose a detailed scheme for assembly of the 43S PIC, mRNA recruitment to this complex, and subsequent steps of scanning and start codon recognition in the 48S PIC. In the empty 40S subunit, the position of the head with respect to the body ensures that the latch is closed (Passmore et al., 2007). Binding of eIF1 and eIF1A to assemble the 40S•eIF1•eIF1A complex (Figure S4A) leads to an 8° rotation of the head compared to the empty 40S subunit in 80S ribosomes (Ben-Shem et al., 2011) that likely facilitates binding of TC in the P_OUT_ state to form the 43S PIC (Figures S4B and S7B). Notably, the latch remains closed in both the 40S•eIF1•eIF1A and py43S complexes observed here. The 40S head is further rotated 5°–6° in the structures of py43S (without eIF3) or pm43S (without eIF1/eIF1A) (Hashem et al., 2013) relative to 40S•eIF1•eIF1A (Figure S7B), which may facilitate mRNA recruitment.

The py48S-open and py48S-closed structures (Figures 1A and 1B) illuminate a series of rearrangements that enable the PIC to first bind and scan the mRNA and then halt upon recognition of the start codon. In py48S-open, the presence of eIF1, eIF1A, TC, and eIF3 provokes an upward movement of the head away from the body, opening the latch and widening the mRNA channel between the body and head, and opening the P site, which leads to diminished contacts with tRNA_i_ relative to the closed state (Figure S7B). We propose that this open conformation enables lateral insertion of the 5′ end of the mRNA—facilitated by the eIF4F complex bound at the cap—onto the 40S subunit (not shown). Once loaded onto the mRNA, py48S-open would be poised for scanning: the mRNA is held loosely in the channel; tRNA_i_ is not fully engaged with the P site; the eIF1A-NTT is disordered. eIF2β interacts with tRNA_i_, eIF1, and eIF1A to both stabilize TC binding and help hold the mRNA in the channel to promote processive scanning. The relocation of the eIF3b-CTD/eIF3i/eIF3g-NTD module near h44 on the intersubunit face, where it interacts with eIF2γ, would promote the same ends. As the open complex scans the mRNA, eIF5-mediated GTP hydrolysis by eIF2 occurs, but P_i_ release is blocked by the presence of eIF1, itself stabilized in the complex by its interaction with the NTD of eIF3c.

In py48S-closed, recognition of the start codon results in downward movement of the head, driven by a change in the pitch of h28 and changes in the orientation of eIF2β, closing the latch and fixing the mRNA in the channel to arrest scanning. Head closure also brings P-site elements in the 40S body into contact with the ASL, locking Met-tRNA_i_ into the P site. Both this constriction of the entry channel and the enclosure of the P site around tRNA_i_ (Figure S7B) are supported by recent hydroxyl radical probing of yeast 48S complexes reconstituted with AUG versus AUC start codons (Zhang et al., 2015). These and other rearrangements stabilize the P_IN_ state of the closed complex (Figure S7B): interactions between eIF2β and the tRNA_i_ are remodeled; eIF2β exchanges its contacts with eIF1 and eIF1A for those with the 40S head; the eIF1A-NTT interacts with the AUG:anticodon duplex. Other rearrangements deform eIF1; different portions of tRNA_i_ are brought into contact with eIF1, adjusting its position and promoting its eviction from the 40S subunit, which provokes P_i_ release and commits the complex to subunit joining. P_i_ release may also trigger detachment of eIF3b-CTD/eIF3i/eIF3g-NTD from the subunit interface, paving the way for release of eIF2, binding of eIF5B, and joining of the 60S subunit. Conformational changes within the TC in py48S-closed also bring the eIF2α-D1 loop in contact with the key −3 nt upstream of the start codon to regulate AUG selection (Hussain et al., 2014).

In summary, the py48S-open and py48S-closed structures described here address long-standing questions about various aspects of initiation. Comparison of these structures reveal how the PIC in the open state may facilitate both loading of the mRNA and subsequent scanning, all while holding both TC and mRNA in place for processive inspection of codons within the P site. Upon recognition of the start codon, the PIC closes, both locking mRNA and tRNA_i_ within the P site and preparing eIF1 for its departure from the complex. Our structures also reveal how eIF3, bound at the 40S solvent face, may encircle the PIC, linking the mRNA entry and exit channels with the locus of action near the P site.

## Experimental Procedures

### Electron Microscopy

Data were collected on an FEI Titan Krios microscope operated at 300 kV under low-dose conditions (27 e^−^/Å^2^) using a defocus range of 1.8–3.2 μm. Images were recorded on a Falcon II detector at a calibrated magnification of 104,478 (yielding a pixel size of 1.34 Å). An in-house system was used to intercept the videos from the detector at a speed of 16 frames/s exposures. Micrographs that showed noticeable signs of astigmatism or drift were discarded.

### Analysis and Structure Determination

Particles were picked using RELION (Scheres, 2012). Contrast transfer function parameters for the micrographs were estimated using CTFFIND3 (Mindell and Grigorieff, 2003). 2D class averaging, 3D classification, and refinements were done using RELION (Scheres, 2012).

Statistical movie processing was done (Bai et al., 2013) to improve resolution of all reconstructions. Resolutions reported are based on the gold-standard FSC = 0.143 criterion (Scheres and Chen, 2012). Local resolution was estimated using RESMAP (Kucukelbir et al., 2014). All maps were further processed for the modulation transfer function of the detector and sharpened by applying negative B factors (−20 Å^2^ for py48S-open and −119 Å^2^ py48S-closed; estimated as in Rosenthal and Henderson, 2003).

### Model Building and Refinement

The atomic model of py48S (PDB: 3J81) was placed into density by rigid-body fitting using Chimera (Pettersen et al., 2004). Further model building was done in Coot (Emsley et al., 2010). For py48S-open, the body and head of the 40S were independently placed. For eIF2β, models from its archaeal counterpart were employed (PDB: 3CW2, 2D74). Wild-type tRNA_i_ was used from PDB: 1YFG for initial rigid-body fitting into its corresponding density in the py48S-open complex. Model building and refinement were carried out using Coot and Refmac (Brown et al., 2015) (see Supplemental Experimental Procedures). All figures were generated using PyMOL (DeLano, 2006) or Chimera.

## Author Contributions

J.L.L. and T.H. made the samples, collected and analyzed the data, determined the structures and wrote a first draft of the manuscript. L.M. and A.T. performed the genetic experiments. C.E.A. provided advice on eIF3 purification, helped characterize recombinant eIF3, and helped write the manuscript. J.R.L., A.G.H., and V.R. supervised the work and helped to write the manuscript.

## Figures and Tables

**Table 1:** Refinement and Model Statistics

	py48S-Open	py48S-Closed	40S•eIF1•eIF1A
**Data Collection**			

Particles	4,547	21,401	86,055
Pixel size (Å)	1.34	1.34	1.34
Defocus range (μm)	1.6–4.0	1.6–4.0	1.6–4.0
Voltage (kV)	300	300	300
Electron dose (e^−^ Å^−2^)	28	28	28

**Model Composition**			

Non-hydrogen atoms	89,774	98,371	77,850
Protein residues	6,413	7,446	5,056
RNA bases	1,855	1,869	1,780

**Refinement**			

Resolution used for refinement (Å)	6.20	5.00	3.50
Map sharpening B-factor (Å)	−20	−119	−81
Average B-factor (Å)	NA	NA	89
R factor #	34.6	38.4	27.7
Fourier Shell Correlation (FSC)^∗^	0.69	0.70	0.85

**Rms deviations**			

Bonds (Å)	0.006	0.007	0.006
Angles (°)	1.00	1.20	1.16

**Validation (proteins)**			

Molprobity score	2.43 (99^th^ percentile)	2.94 (90^th^ percentile)	2.87 (92^th^ percentile)
Clashscore, all atoms	4.09 (100^th^ percentile)	9.79 (97^th^ percentile)	7.63 (97^th^ percentile)
Good rotamers (%)	93.4	88.4	88.2

**Ramachandran plot**			

Favored (%)	87.3	86.8	86.0
Outliers (%)	3.3	3.3	3.8

**Validation (RNA)**			

Correct sugar puckers(%)	96.5	95.5	96.8
Good backbone conformations(%)	55.6	50	62.5

## Accession Numbers

Maps have been deposited in the EMDB with accession codes EMD: EMD-3047, EMD-3048, EMD-3049, EMD-3050 for the 40S•eIF1•eIF1A, py48S-closed, py48S-open, and py43S, respectively. Atomic coordinates have been deposited in the PDB with accession codes PDB: 3JAM, 3JAP, 3JAQ for 40S•eIF1•eIF1A, py48S-closed, and py48S-open, respectively.

## Supplemental Information

Document S1. Figures S1–S7, Table S1, and Supplemental Experimental Procedures

Movie S1. py48S-Closed Complex This movie shows a 360° rotation of the map of the py48S-closed complex, followed by the fitting of refined coordinates in the map. The fitting of ligands can also be observed with the maps shown as a transparent surface. The β-propeller of eIF3b shown at the solvent interface is modeled and not present in the py48S-closed complex.

Movie S2. py48S-Open Complex This movie shows a 360° rotation of the map of py48S-open complex, followed by the fitting of refined coordinates in the map. The fitting of ligands can also be observed with maps shown as a transparent surface.

Movie S3. Morphing of PICs: py48S-Open to py48S-Closed Complex This movie shows the morphing of 18S rRNA in the py48S-open to the py48S-closed complex (colored cyan in the first frame). A short region (1,148–1,163; 1,615–1,627) in helix h28 is shown in red. Most ligands (except tRNAi and eIF2α) and all ribosomal proteins have been removed for clarity. The front view shows the upward movement of the head while no major conformational change is observed in the body. The change in position of tRNAi and eIF2α with the head movement can be clearly seen.

Movie S4. Morphing of PICs: 40S•eIF1•eIF1A PIC to py48S-Closed Complex This movie shows the morphing of 18S rRNA in the 40S•eIF1•eIF1A PIC to the py48S-closed complex (colored blue in the first frame). A short region (1,148–1,163; 1,615–1,627) in helix h28 is shown in red. All ligands and ribosomal proteins have been removed for clarity. The front view shows the rotation of the head while no major conformational change is observed in the body.

Movie S5. Morphing of Ligands: py48S-Open to py48S-Closed Complex This movie shows the morphing of the 18S rRNA and ligands in the py48S-open to the py48S-closed complex. The ligands are shown in color, as in Figure 1. Only the eIF3i subunit is shown for eIF3. All ribosomal proteins have been removed for clarity. This movie shows the conformational change that TC undergoes during the transition from the open to the closed state.

Document S2. Article plus Supplemental Information
