# Supplementary material for: Conformational Differences between Open and Closed States of the Eukaryotic Translation Initiation Complex
Source: Mol Cell. 2015 Aug 6;59(3):399–412. doi: 10.1016/j.molcel.2015.06.033 (PMC4534855; doi:10.1016/j.molcel.2015.06.033)
Supplement: Document S1. Figures S1–S7, Table S1, and Supplemental Experimental Procedures [file mmc1.pdf]

Molecular Cell, Volume *59*

Supplemental Information

Conformational Differences

between Open and Closed States

# of the Eukaryotic Translation Initiation Complex

Jose L. Llácer, Tanweer Hussain, Laura Marler, Colin Echeverría Aitken, Anil Thakur, Jon R. Lorsch, Alan G. Hinnebusch, and V. Ramakrishnan

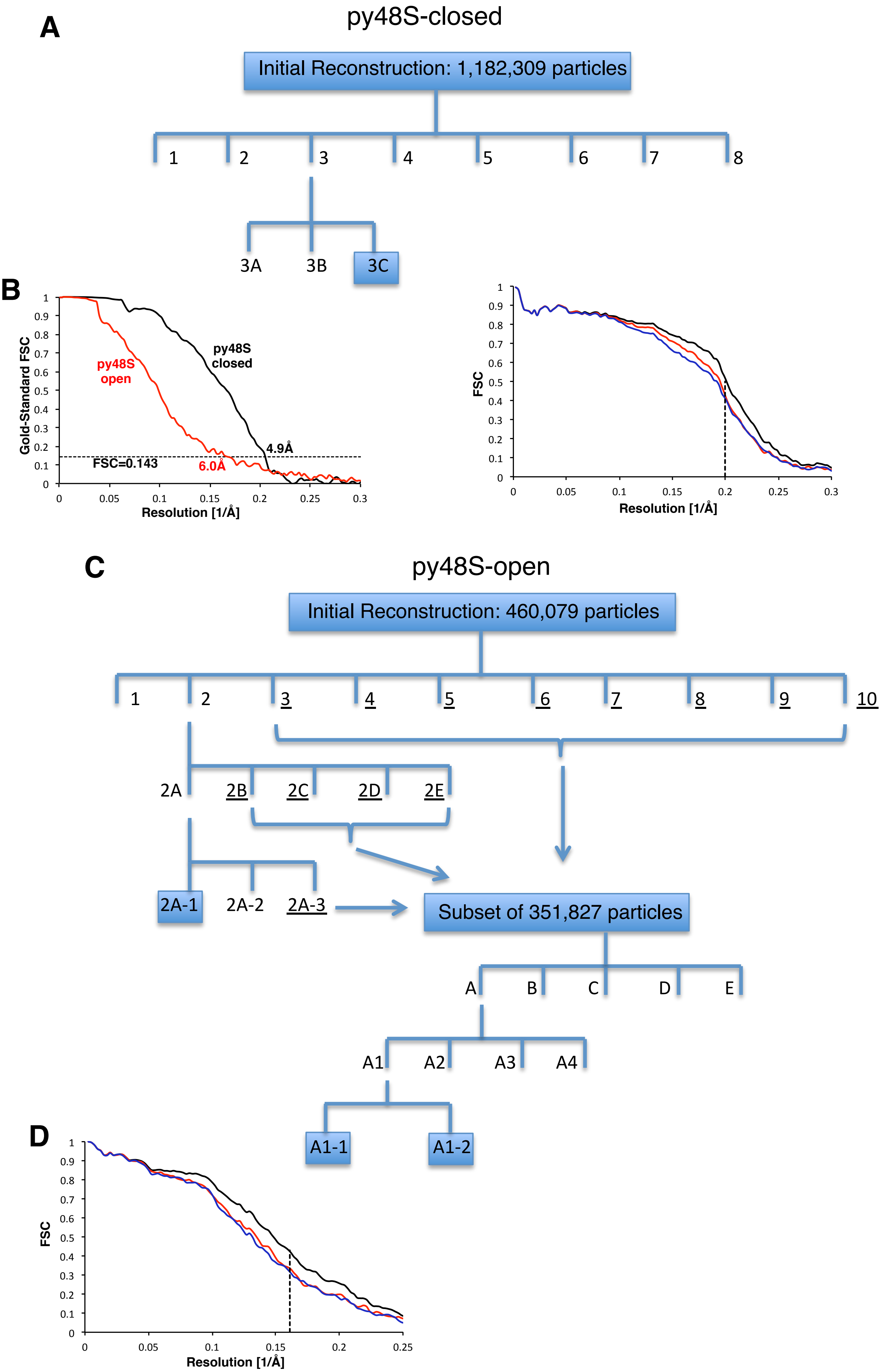
A
py48S-closed
Initial Reconstruction: 1,182,309 particles
1
2
3
4
5
6
7
8
3A
3B
3C
B
Gold-Standard FSC
py48S open
py48S closed
4.9Å
6.0Å
FSC=0.143
Resolution [1/Å]
FSC
Resolution [1/Å]
C
py48S-open
Initial Reconstruction: 460,079 particles
1
2
3
4
5
6
7
8
9
10
2A
2B
2C
2D
2E
2A-1
2A-2
2A-3
Subset of 351,827 particles
A
B
C
D
E
A1
A2
A3
A4
A1-1
A1-2
D
FSC
Resolution [1/Å]

Figure S1. Maximum-likelihood 3D classification scheme and data processing quality of density maps, related to Figure 1

Maximum-likelihood 3D classification schemes (See Supplemental Experimental Procedures):

(A) py48S-closed complex: Class 3C (21,401 particles; 4.9 Å) corresponding to py48S-closed complex is highlighted in a box.

(B) At the left, Gold-standard Fourier Shell Correlation (FSC) curves for the py48S-closed (black) and py48S-open (red) complexes. At the right, analysis of overfitting by cross-validation of the py48S-closed model. FSC_{work} curves (red) corresponding to the refined model versus the half-map it was refined against, and FSC_{test} curves (blue), i.e. those calculated between the refined atomic model and the other half-map. The black curve shows the FSC curve between a reconstruction from all particles and the model refined against the map. The dashed line represents the highest resolution (5.0Å) used in these refinements.

(C) py48S-open complex: Class 2A-1 (86,055 particles; 3.5 Å) corresponding to 40S•eIF1•eIF1A complex, Class A1-1 (4,547 particles; 6.0 Å) corresponding to py48S-open complex and Class A1-2 (1,580 particles; 14.9 Å) corresponding to py43S are highlighted in a box.

(D) Analysis of overfitting by cross-validation of the py48S-open model. FSC_{work} curves (red) corresponding to the refined model versus the half-map it was refined against, and FSC_{test} curves (blue), i.e. those calculated between the refined atomic model and the other half-map. The black curve shows the FSC curve between a reconstruction from all particles and the model refined against the map. The dashed line represents the highest resolution (6.2Å) used in these refinements.

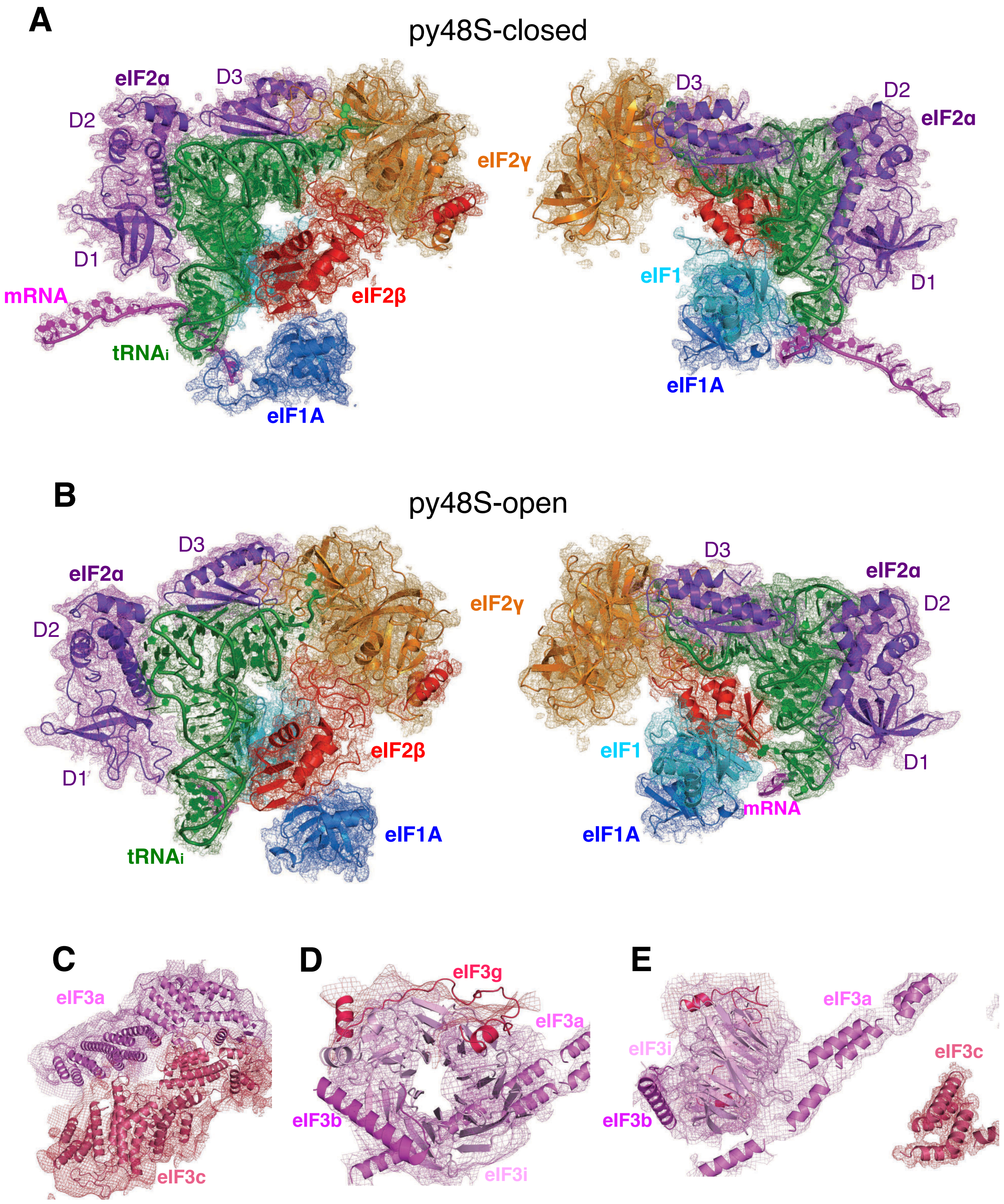

Figure S2. Fitting of ligands in density maps, related to Figure 1

(A) Fitting of eIF1, eIF1A, eIF2, tRNAi and mRNA in py48S-closed map at 4.9 Å shown in two orientations.

(B) Fitting of eIF1, eIF1A, eIF2, tRNAi and mRNA in py48S-open map at 6.0 Å shown in two orientations.

(C) eIF3a/eIF3c PCI heterodimer in py48S-closed map.

(D) eIF3b-CTD/eIF3i/eIF3g-NTD in py48S-closed map.

(E) eIF3b-CTD/eIF3i/eIF3g-NTD trimer, cluster of eIF3c helices and bundle of eIF3a long helices in py48S-closed map.

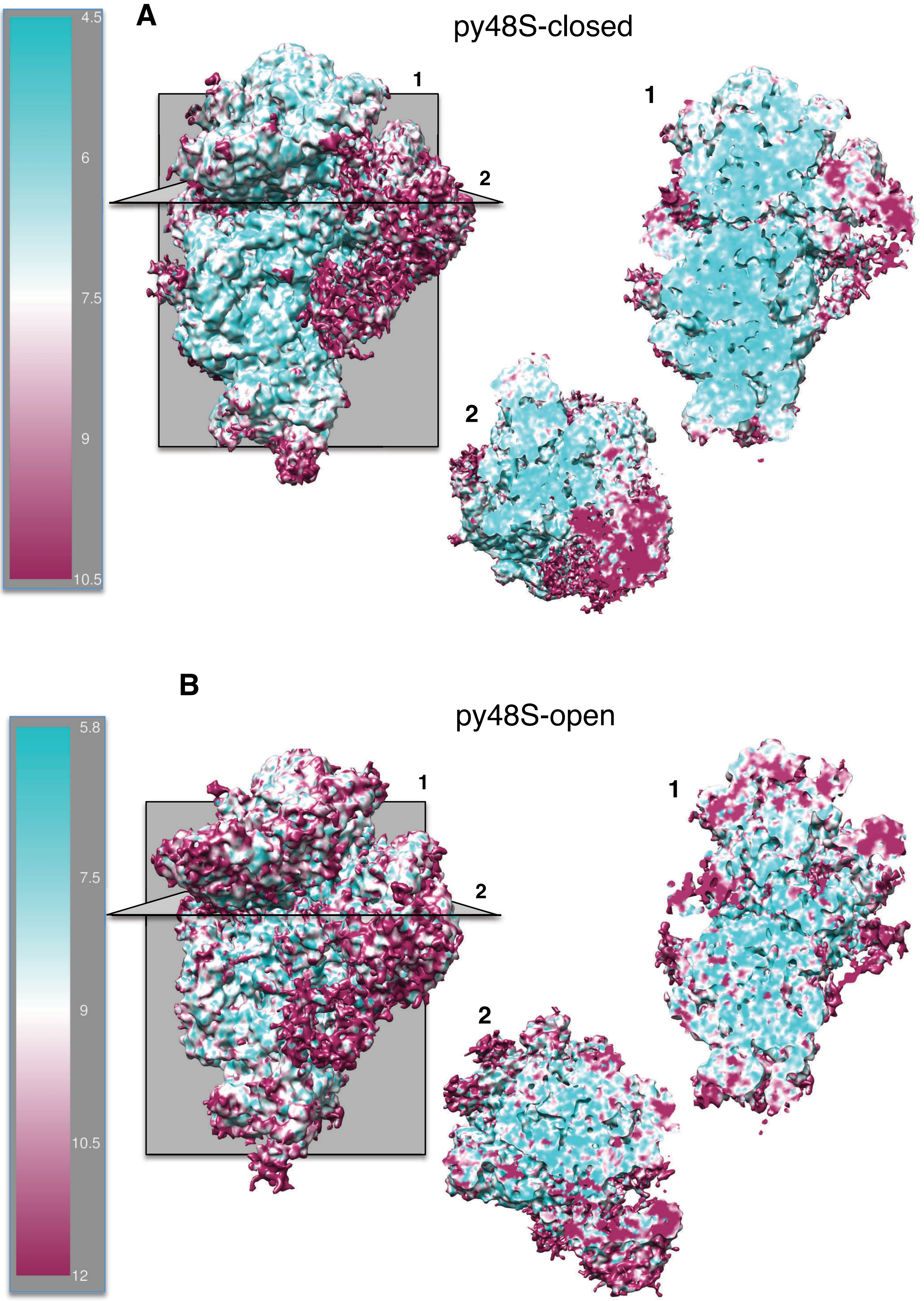
A
py48S-closed
4.5
6
7.5
9
10.5
1
2
1
2
B
py48S-open
5.8
7.5
9
10.5
12
1
2
1
2

Figure S3. Local resolution features, related to Figure 1

(A) Surface (left) and cross-sections along the mRNA channel in two different planes of an 4.9 Å map, colored according to local resolution (See Experimental Procedures) of py48S-closed complex.

(B) Surface (left) and cross-sections along the mRNA channel in two different planes of an 6.0 Å map, colored according to local resolution (See Experimental Procedures) of py48S-open complex.

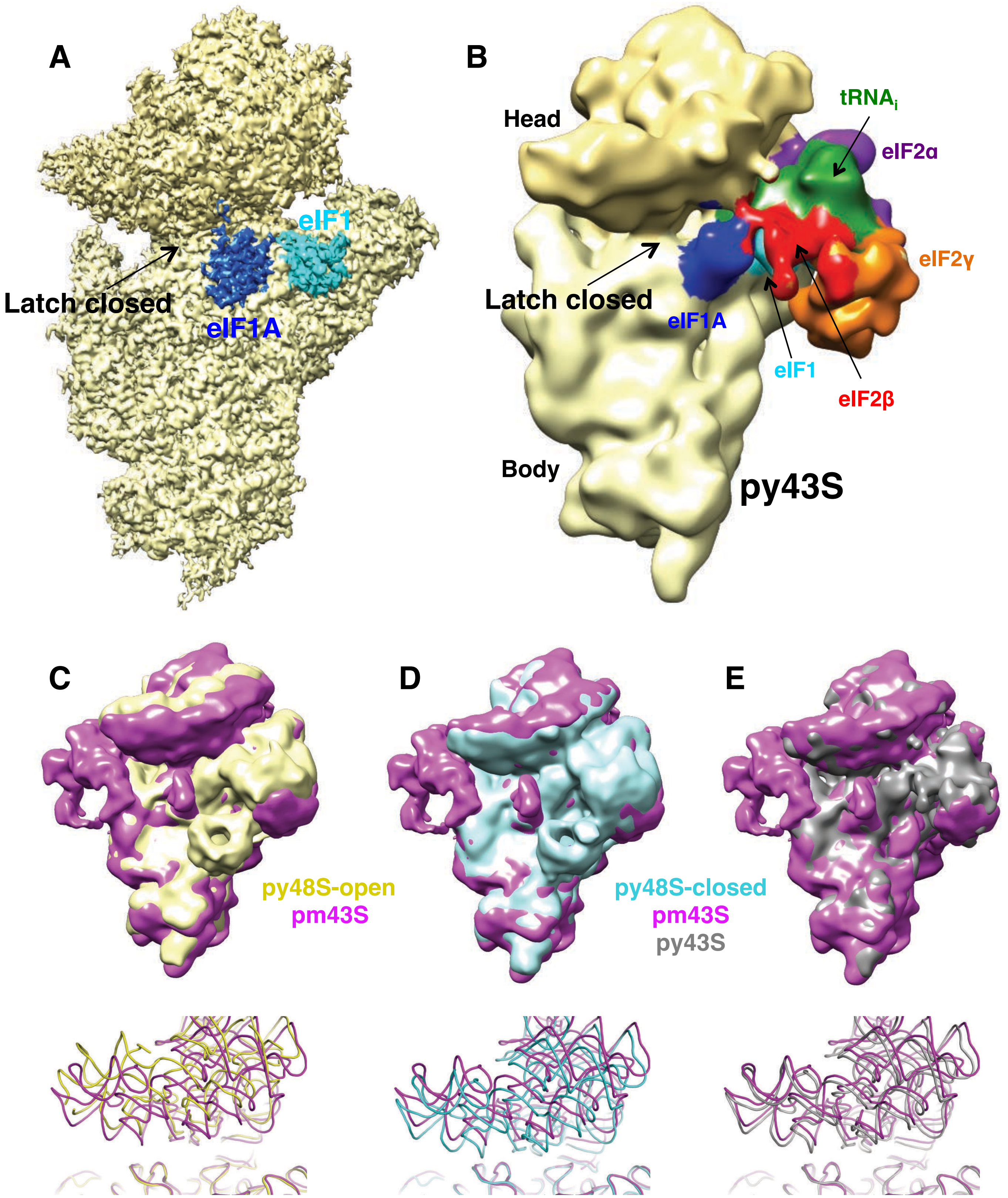
A
Latch closed
eIF1A
eIF1
B
Head
Body
Latch closed
tRNA$_i$
eIF2α
eIF2γ
eIF1A
eIF1
eIF2β
py43S
C
D
E
py48S-open
pm43S
py48S-closed
pm43S
py43S

Figure S4. Cryo-EM maps of py43S and 40S•eIF1•eIF1A complexes, related to Figures 1 and 2
(A) Cryo-EM map of 40S•eIF1•eIF1A PIC at 3.5 Å. Density for eIF1 (cyan) and eIF1A (blue) can be clearly seen. The structure of the 40S•eIF1•eIF1A complex is similar to the PIC-2 complex reported earlier (Hussain et al., 2014) with an r.m.s.d. of 0.99 Å for 35,235 atoms of 18S rRNA.
(B) Cryo-EM map of py43S PIC at 15.0 Å. Density for eIF1, eIF1A and TC is observed.
(C) Superimposition of py48S-open (yellow) and pm43S (magenta) (Hashem et al., 2013) maps. *Below:* Cartoon representation of rRNAs of the two structures. The head is clearly moved up in py48S-open. The py48S-open map is low-pass filtered to 12 Å.
(D) Superimposition of py48S-closed (cyan) and pm43S (magenta) (Hashem et al., 2013) maps. *Below:* Cartoon representation of rRNAs of the two structures. The head is in a similar but not identical position in the two complexes. The py48S-closed map is low-pass filtered to 12 Å.
(E) Superimposition of py43S (grey) and pm43S (magenta) (Hashem et al., 2013) maps. *Below:* Cartoon representation of rRNAs of the two structures. The head is almost identical in both complexes.

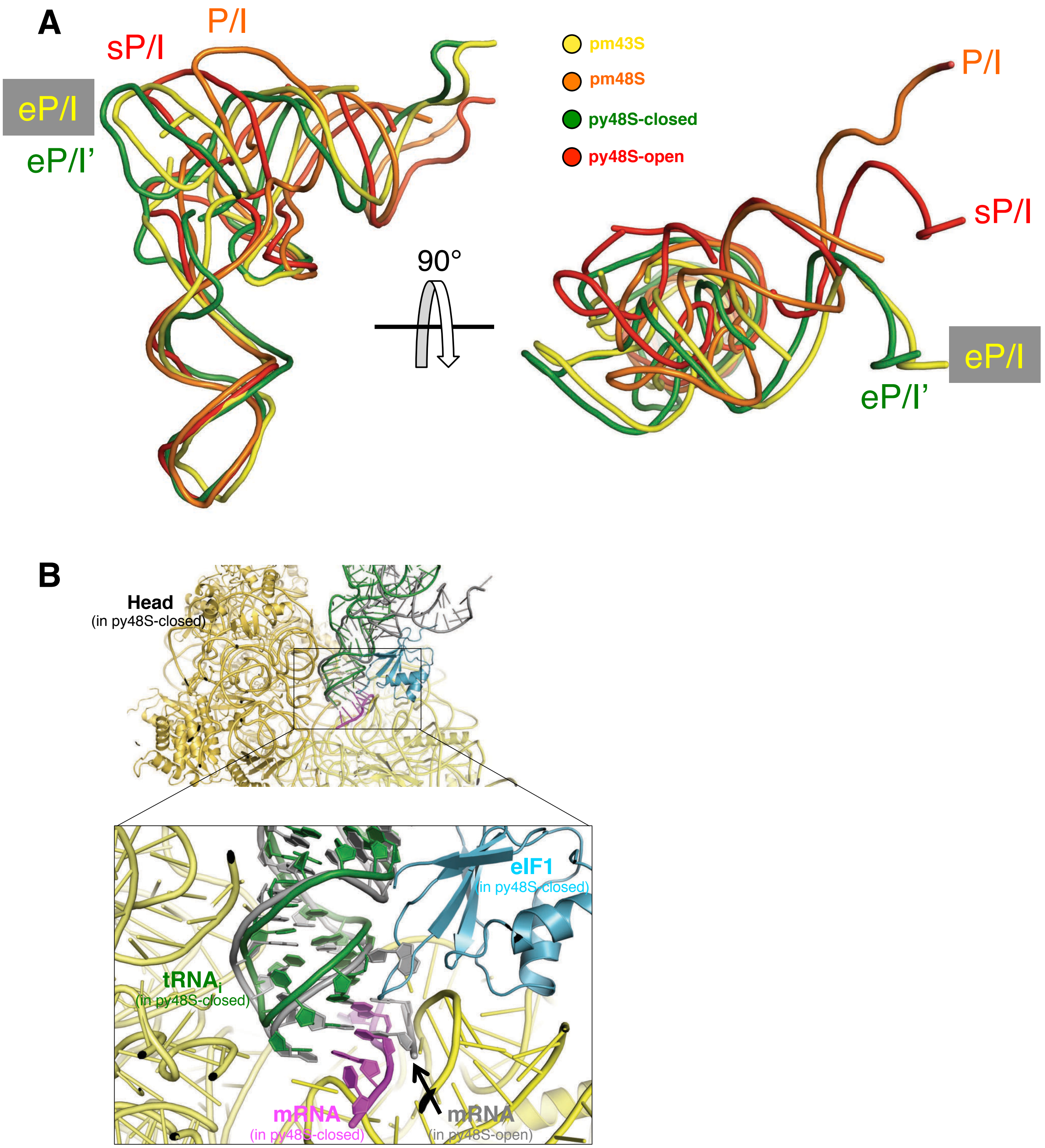

Figure S5. Distinct tRNA$_i$ conformations and the mRNA path, related to Figure 3

(A) Two different views of the superimposition of tRNA$_i$ from different complexes aligned to the head of 40S. The conformation of tRNA$_i$ from py48S-closed (eP/I’, green) is similar to that described for pm43S complex [eP/I**,** yellow; (Hashem et al., 2013)], but different from the P/I conformation (from pm48S PIC, in orange, from 4KZZ). The tRNA$_i$ from py48S-open (red) complex is in an orientation that appears closer to P/I than the eP/I. We have termed this orientation sP/I (scanning P/I).

(B) Superimposition of py48S- open and closed complexes aligned to the 40S head shows the relative position of tRNA$_i$ in the P site. The body and head of py48S-closed complex is shown in yellow with its tRNA$_i$ in green. The mRNA (magenta) and β-hairpin 1 of eIF1 (cyan) of py48S-closed complex interact at the P site. The mRNA and tRNA$_i$ of py48S-open complex is shown in grey. The mRNA in the py48S-open complex would clash with the body of 40S in the py48S-closed complex.

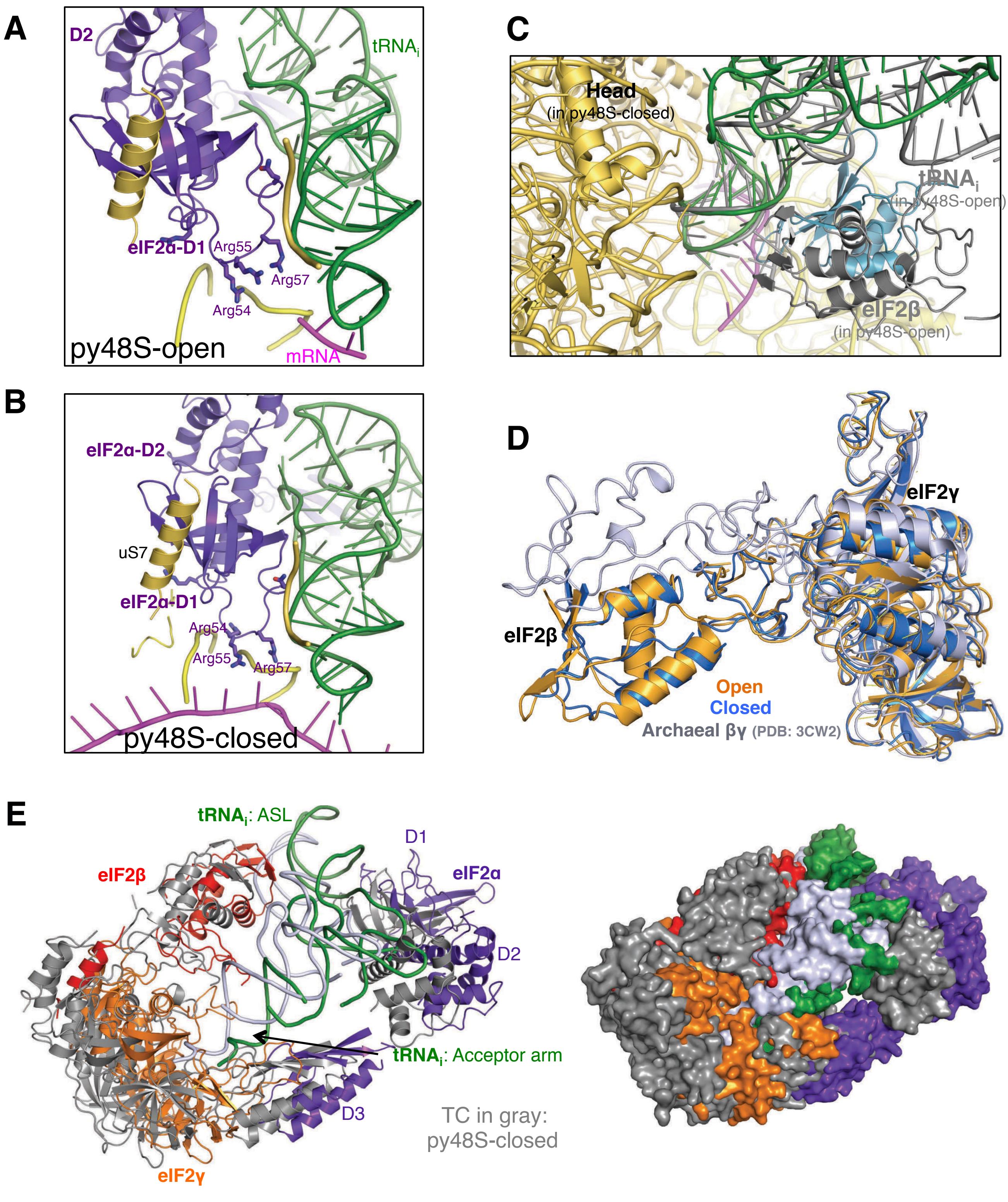

A
D2
tRNA$_i$
eIF2α-D1
Arg55
Arg57
Arg54
py48S-open
mRNA
B
eIF2α-D2
uS7
eIF2α-D1
Arg54
Arg55
Arg57
py48S-closed
C
Head
(in py48S-closed)
tRNA$_i$
(in py48S-open)
eIF2β
(in py48S-open)
D
eIF2γ
eIF2β
Open
Closed
Archaeal βγ (PDB: 3CW2)
E
tRNA$_i$: ASL
D1
eIF2β
eIF2α
D2
tRNA$_i$: Acceptor arm
D3
TC in gray:
py48S-closed
eIF2γ

Figure S6. Relative orientations of eIF2 in py48S- open and closed PICs, related to Figure 5
(A) eIF2α-D1 and ASL of $tRNA_i$ in py48S-open complex. The neighbouring rRNA residues are shown in yellow lines and a helix of uS7 is also shown in yellow. Conserved arginines are shown. Arg55 and Arg57 project away from the modeled mRNA (grey; from py48S-closed complex), and Arg54, which in the closed complex interacts with the body of the 40S, comes closer to the mRNA.
(B) eIF2α-D1 and the ASL of $tRNA_i$ in the py48S-closed complex. The neighbouring rRNA residues are shown in yellow lines and a helix of uS7 is also shown in yellow. Conserved arginines are shown. Arg55 and Arg57 interact with the mRNA (magenta).
(C) Superimposition of py48S- closed and open complexes shows that eIF2β of py48S-open complex (grey) would clash with eIF1 (cyan) of py48S-closed complex, highlighting the need for the conformational change within the TC during the open to closed PIC transition. The 40S, $tRNA_i$, mRNA and eIF1 of the py48S-closed complex are shown in color while only $tRNA_i$ and eIF2β of py48S-open complex are shown in grey.
(D) Superimposition of eIF2-βγ dimer of py48S- open (yellow) and closed (blue) complexes with the most similar archaeal βγ dimer (grey, from 3CW2) using γ as a reference shows different position of the β subunit with respect to the γ subunit.
(E) Relative position of TC in py48S-open (color) and py48S-closed (grey) based on superposition of the two complexes. The cartoon and surface representation show the relative position of each component of the TC.

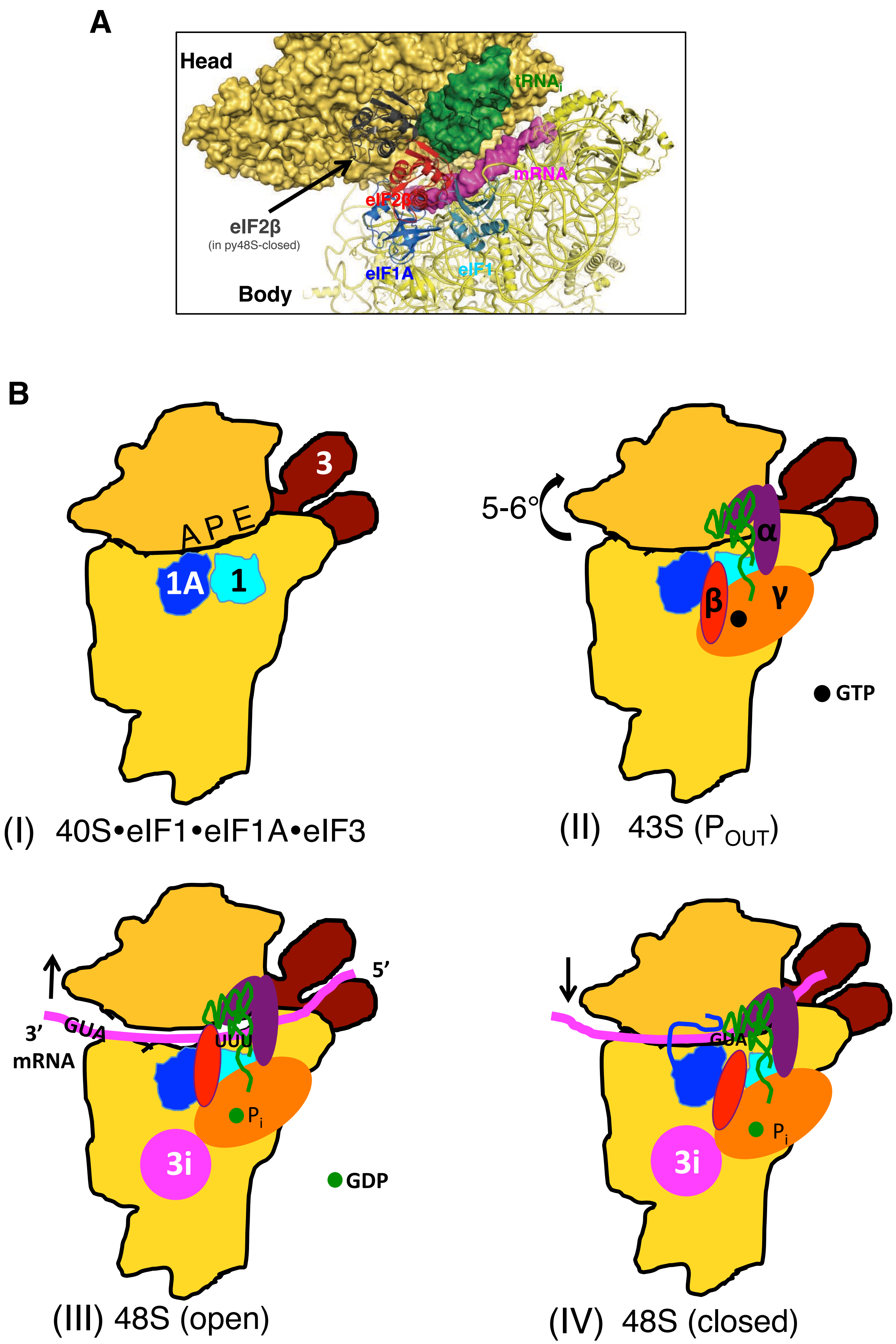
A
Head
tRNAi
mRNA
eIF2β
eIF2β
(in py48S-closed)
eIF1A
eIF1
Body
B
3
A P E
1A
1
(I) 40S•eIF1•eIF1A•eIF3
5-6°
α
β
γ
GTP
(II) 43S (POUT)
5'
3'
mRNA
GUA
UUU
Pi
3i
GDP
(III) 48S (open)
GUA
Pi
3i
(IV) 48S (closed)

Figure S7: Conformational changes from open to closed state, related to Figure 5

(A) Opening of the mRNA channel. Modeling of the position of eIF2β (grey) (with respect to 40S head and $tRNA_i$) observed in the py48S-closed complex into the py48S-open complex shows how in this conformation the mRNA channel would be opened up. In the py48S-open complex, eIF2β (red) blocks mRNA access by forming interactions with $tRNA_i$ (green) attached to head and eIF1 (cyan) and eIF1A (blue) attached to the body. The acceptor arm of $tRNA_i$ is not shown for clarity.

(B) Major structural changes during eukaryotic translation initiation. Binding of eIF1, eIF1A and eIF3 to the 40S subunit (I) facilitates TC binding in the P_{OUT} conformation to form the 43S PIC (II). Upward movement of the head expands the mRNA entry channel, allowing mRNA recruitment, and widens the P site to form the scanning-conducive py48S-open (III). eIF2β contacts eIF1 and probably stabilizes this open conformation while eIF3 undergoes major conformational change and eIF3i is repositioned on the subunit-interface. On AUG recognition, the head moves downward to clamp in the mRNA and enclose the $tRNA_i$ in the P_{IN} state of py48S-closed (IV). eIF2β loses contact with eIF1 and moves away. (See text for further details.)

Supplementary Table

Table S1. Local resolution, Related to Figure 1.

**Local resolution**	**py48S-open**	**py48S-closed**	**40S• eIF1• eIF1A**	**py48S calculated from EMD-2763** (Hussain et al., 2014)
Overall	6.00	4.90	3.45	4.00
Body of 40S	6.00	4.90	3.40	4.05
Head of 40S	6.30	5.05	3.55	4.10
eIF1	7.90	5.30	3.65	6.80
eIF1A	7.20	5.10	3.70	4.60
$tRNA_i$	7.90	5.50	-	5.90
eIF2α	8.75	6.25	-	6.85
eIF2β	9.15	7.15	-	-
eIF2γ	9.35	10.30	-	14.90
ASL of $tRNA_i$ and mRNA (-4 to +4)	7.30	4.80	-	4.70
eIF3-bgi subcomplex	10.30	8.30	-	-
eIF3c 5-helix bundle	7.05	5.70	-	-
eIF3 PCI domains	-	13.80	-	-

Supplementary Movie Legends

Movie S1: py48S-closed complex, related to Figure 1
This movie shows a 360° rotation of the map of the py48S-closed complex, followed by the fitting of refined coordinates in the map. The fitting of ligands can also be observed with the maps shown as a transparent surface. The β-propeller of eIF3b shown at the solvent interface is modeled based on previously reported structure (Aylett et al., 2015).

Movie S2: py48S-open complex, related to Figure 1
This movie shows a 360° rotation of the map of py48S-open complex, followed by the fitting of refined coordinates in the map. The fitting of ligands can also be observed with maps shown as a transparent surface.

Movie S3: Morphing of PICs: py48S-open to py48S-closed complex, related to Figure 2
This movie shows the morphing of 18S rRNA in the py48S-open to the py48S-closed complex (colored cyan in the first frame). A short region (1148-1163; 1615-1627) in helix h28 is shown in red. Most ligands (except $tRNA_i$ and eIF2α) and all ribosomal proteins have been removed for clarity. The front view shows the upward movement of the head while no major conformational change is observed in the body. The change in position of $tRNA_i$ and eIF2α with the head movement can be clearly seen.

Movie S4: Morphing of PICs: 40S•eIF1•eIF1A PIC to py48S-closed complex, related to Figure 2
This movie shows the morphing of 18S rRNA in the 40S•eIF1•eIF1A PIC to the py48S-closed complex (colored blue in the first frame). A short region (1148-1163; 1615-1627) in helix h28 is shown in red. All ligands and ribosomal proteins have been removed for clarity. The front view shows the rotation of the head while no major conformational change is observed in the body.

Movie S5: Morphing of ligands: py48S-open to py48S-closed complex, related to Figure 5

This movie shows the morphing of the 18S rRNA and ligands in the py48S-open to the py48S-closed complex. The ligands are shown in color, as in Figure 1. Only the eIF3i subunit is shown for eIF3. All ribosomal proteins have been removed for clarity. This movie shows the conformational change that TC undergoes during the transition from the open to the closed state.

Supplemental Experimental Procedures

Recombinant eIF3 production

In order to obtain an initiation complex in an open scanning-competent state, eIF5 was omitted in the preparation. Since eIF5 often copurifies with eIF3 in *S. cerevisiae* (Acker et al., 2007; Hussain et al., 2014), we overexpressed eIF3 in *Escherichia coli* as follows. The genes for subunits of eIF3 were cloned into two different but compatible polycistronic vectors: eIF3a and eIF3c were cloned into a pCDF Duet vector including an N-terminal his-tag for subunit a, and eIF3b, eIF3g and eIF3i in a pQlink vector. These two plasmids were used to transform *E. coli* Rosetta cells and the expression was carried out at 30 °C after induction with 0.5 mM IPTG. The protein was purified using the same steps used for the protein expressed in yeast (Mitchell et al., 2010). The protein obtained is soluble, pure and seems to be expressed in stoichiometric amounts (judged by SDS-PAGE). However visualization of the purified protein by SDS-PAGE shows that the eIF3c and the eIF3g subunits are slightly smaller than expected. Mass spectrometry analysis of these bands suggests that they may be missing part of their N-terminal region due to possible proteolysis (up to 42 N-terminal residues of its 812 amino acids for eIF3c and up to 65 N-terminal residues of its 274 amino acids for eIF3g). Nonetheless, the protein seems to be functional: it is able to interact with the other eIF3 subunit (subunit eIF3j), with the 40S ribosomal subunit, and it promotes mRNA recruitment using the assay described by (Mitchell et al., 2010), with a K_{extent} (defined as the concentration of eIF3 necessary for half maximal extent of mRNA recruitment) similar to that of eIF3 purified from yeast.

Reconstitution of 48S complexes

K. lactis 40S subunits were prepared as described earlier (Fernandez et al., 2014). *S. cerevisiae* eIF3 and eIF2 were expressed in yeast while eIF1, eIF1A and eIF5 were expressed in *E. coli* as recombinant proteins and purified as described (Acker et al., 2007). Recombinant eIF3 used for preparation of py48S-open complex was expressed in *E. coli.* Wild type $tRNA_i$ was expressed and purified from yeast and mutant $tRNA_i$ was transcribed and aminoacylated as described (Acker et al., 2007). Unstructured mRNAs with AUG (5′ GGAA$[UC]_4$U<u>AUG</u>$[CU]_4$C 3′) and AUC (5′ GGAA$[UC]_4$U<u>AUC</u>$[CU]_4$C 3′) codons were commercially synthesized by Integrated DNA Technologies and used for the py48S-closed and py48S-open complexes, respectively. Both complexes were reconstituted by incubating 120 nM 40S with eIF1, eIF1A, TC (consisting of eIF2, GDPCP and Met-$tRNA_i$), eIF3, eIF5 and mRNA in 40S:eIF1:eIF1A:TC:eIF3:eIF5:mRNA molar ratios of 1:2.5:2.5:1.5:1.2:2.5:2, with the exception that eIF5 was excluded in py48S-open, in 20 mM MES, pH 6.5, 40 mM potassium acetate, 10 mM ammonium acetate, 8mM magnesium acetate, 2mM dithiothreitol. The sample was used directly to make cryo-EM grids without further purification. Grids with sample for electron microscopy were prepared as described (Fernandez et al., 2014).

Analysis, structure determination, model building and refinement

Details of 3D classification

For py48S-closed complex data set

From about 5500 micrographs, a total of approximately 1,200,000 particles were picked. 2D class averaging was performed and aberrant particles were discarded. An initial reconstruction was made from all selected particles (1,182,309) after 2D class averaging using the yeast 40S crystal structure low pass filtered to 40 Å as an initial model. Next, a 3D classification into 8 classes with fine angular sampling was performed. Upon refinement only two classes were refined to high resolution: class 3 [12.6 %; 149,369 particles; 4.6 Å; PIC with TC] and class 4 [31.6 %; 374,737 particles; similar to PIC-2 (Hussain et al., 2014)]. The class 3, which showed a PIC with TC, was further classified into 3 classes: 3A [15,044 particles; 7.4 Å], 3B [112,924 particles; 4.3 Å; similar to py48S (Hussain et al., 2014)] and 3C [21,401 particles; 4.9 Å; py48S-closed complex].

For py48S-open complex data set

A data set of more than 2000 images was collected and about 500,000 particles were picked. An initial reconstruction was made from all selected particles (460,079) after 2D class averaging using the yeast 40S crystal structure low pass filtered to 40 Å as an initial model. Next, a 3D classification into 10 classes with fine angular sampling was performed. Class 1 showed the presence of a 40S dimer and was discarded. Class 2 showed the presence of TC. Classes 3-10 were not homogenous enough and showed the presence of at least eIF1A. Class 2 was subsequently divided into 5 classes: 2A, 2B, 2C, 2D and 2E. Class 2A was comprised of 97,864 particles and showed the presence of TC. It was again further divided into 3 classes: 2A-1, 2A-2 and 2A-3. Class 2A-1, comprised of 86,055 particles, consisted of the 40S•eIF1•eIF1A complex and was refined to a resolution of 3.5 Å. Class 2A-2 was comprised of 5,174 particles and shows a PIC containing TC but without eIF3, similar to py48S (Hussain et al., 2014), at about 7 Å resolution. Surprisingly this class does not seem to have density for eIF1. Class 2A-3 was not homogenous enough to be refined to moderate resolution. Hence we made a subset of 351,827 particles by combining Classes 3-8 from the first round of classification, Classes 2B-2E from the second round and Class 2A-3 from the third round. In other words, we left out Class 1 (which contained 40S dimers); Class 2A-1 (40S•eIF1•eIF1A) and Class 2A-2 (PIC with eIF1A and TC but without eIF3 and eIF1). This subset was then divided into 5 classes: A, B, C, D and E. Class A contained TC (70,365 particles) and it was then subsequently classified into 4 classes: A1, A2, A3 and A4. Only class A1 (6,127 particles; 6.1 Å) showed the presence of TC with eIF3. Class A1 was then further classified into 2 classes: A1-1 and A1-2. Class A1-1 (4,547 particles; 6.0 Å) represented the most complete class in this data set and contained 40S with eIF1, eIF1A, TC and eIF3, described here as the py48S-open complex, while Class A1-2 (1,580 particles; 14.9 Å) contains a PIC with eIF1, eIF1A and TC (without eIF3) corresponding to the previously reported pm43S complex (Hashem et al., 2013).

Detailed model building

Initially, the atomic coordinates of py48S (PDB: 3J81) were placed into the EM density of py48S-closed complex by rigid-body fitting using Chimera (Pettersen et al., 2004). For py48S-open, the body and head of the 40S of this same model were independently placed. Previously, 40S model had been further improved using the 40S•eIF1•eIF1A structure at 3.5 Å presented here. Then, each chain of the model (including ribosomal proteins, rRNA segments, protein factors and $tRNA_i$ and mRNA) was rigid-body fitted in Coot (Emsley et al., 2010) to overcome local differences in its positions. When necessary, also each separate domain of proteins was also subject to independent rigid body fitting, as was the case of factor eIF2α.

Most of eIF2β was not present in py48S (PDB: 3J81). In both open and closed complexes the relative orientation of eIF2β and eIF2γ is the same. There are three different published archaeal IF2 βγ (aIF2βγ) dimer crystal structures(Stolboushkina et al., 2008; Sokabe et al., 2006; Yatime et al., 2007), which substantially differ in the relative orientation of the two subunits. The most similar to ours correspond to PDB: 3CW2 (Stolboushkina et al., 2008), hence we superimposed aIF2βγ using our eIF2γ as a reference, and then we rigid-body fit the β-subunit independently. However, in this crystal structure used as a model, the ZBD is disordered. Therefore we used the ZBD in PDB: 2D74 to model it.

In py48S-open, wild type $tRNA_i$ was used from PDB: 1YFG for initial rigid-body fitting into its corresponding density and further improvement of the fitting was done with the morphing tool in Coot. Also in py48S-open NTT of eIF1A was removed from the model and eIF1 model was substituted by its counterpart in the 40S•eIF1•eIF1A structure. Finally, we observed a density close to the bases U and A from the anticodon of the $tRNA_i$. We reason this density most likely belongs to bases A and U from the mRNA and in consequence this fragment of mRNA was included in the final py48S-open model.

Model Building of eIF3

Fitting of eIF3a/eIF3c PCI dimer

Although the overall densities for eIF3 at the subunit interface were similar in the two structures, the higher-resolution py48S-closed map at 4.9 Å was used to generate a model for eIF3 bound in these initiation complexes. First the dimer of complete PCI domains of eIF3a and eIF3c was generated using the crystal structures of the full PCI domain of eIF3a (PDB: 4U1D) and the eIF3a/eIF3c PCI dimer (PDB: 4U1C) from *S. cerevisiae*. (Erzberger et al., 2014). This complete eIF3a/eIF3c PCI dimer was docked as a rigid body into the density on the solvent face of the 40S in py48S-close complex. Because of variation in the resolution of the eIF3 domains, we cannot resolve individual helices for the eIF3a/eIF3c PCI domains, however, it is possible to discern the overall shape and dimensions of PCI domains and do a rigid-body fit of the eIF3a/eIF3c PCI dimer. The fitting is similar to that of the eIF3a/eIF3c PCI heterodimers in the recent yeast 40S•eIF1•eIF1A•eIF3 structure (Aylett et al., 2015) and also to the PCI•MPN core model (PDB: 3J7K) docked into the pm43S EM map (Hashem et al., 2013).

Fitting of eIF3i and associated eIF3b-CTD and eIF3g-NTD

Density for a β-propeller domain was observed near h44 in the vicinity of eIF2γ. There are β-propeller domains in two subunits of eIF3: eIF3b and eIF3i. Crystal structures of both domains (PDB: 4U1E, 4U1F) from *S. cerevisiae* are now available (Erzberger et al., 2014). The nine-bladed β-propeller domain of eIF3b has been well documented to interact at the solvent (Liu et al., 2014; Erzberger et al., 2014) rather than the intersubunit face of the 40S as also observed in the yeast 40S•eIF1•eIF1A•eIF3 structure (Aylett et al., 2015). Moreover, the nine-bladed β-propeller domain of eIF3b is larger than the observed density near h44. In fact, rigid body fitting of the β-propeller domain of eIF3b shows a steric clash with 40S.

In contrast, the seven-bladed β-propeller domain of eIF3i fits well into the density. At the local resolution, it is not possible to discern its individual β-strands but the overall shape and dimensions guides the fitting of eIF3i. In one of the two crystal structures of eIF3i (PDB: 3ZWL; (Herrmannova et al., 2012)), there is a loop (residues 258-273) emanating from the β-propeller. A single mutation of a residue of this eIF3i loop confers a severe decrease of translation initiation without affecting the integrity of eIF3 (Cuchalova et al., 2010). We observe density for this loop in a slightly different conformation and interacting with h44. eIF3i also makes an interaction with a long helix at the C-terminus of eIF3b (PDB: 4U1E, 3ZWL). We observe density for this helix, interacting with the β-propeller domain further supporting that this density belongs to eIF3i and not eIF3b. Extra density was also observed for a portion of eIF3g-NTD in direct contact with this β-propeller domain that is consistent with the eIF3b-CTD/eIF3i/eIF3g-NTD trimeric crystal structure (PDB: 4U1E). These observations strongly suggest that the β-propeller domain is part of eIF3i. Moreover, its contacts with eIF3b and eIF3g make it possible to orient the β-propeller domain despite the fact that the individual blades cannot be resolved.

The C-terminal helix of this stretch of eIF3g-NTD points towards the entry channel, where the remainder of the protein (not resolved here) likely binds. Similarly, the N-terminal end of the eIF3b-CTD segment in the trimeric subcomplex points towards the likely position of the eIF3b β-propeller domain and these further supports the positioning of eIF3b-CTD/eIF3i/eIF3g-NTD trimer at the subunit interface near h44 in the vicinity of eIF2γ. As we observe density for only a portion of the eIF3bβ-propeller domain in py48S-close complex, the exact length of the connector between the two eIF3b domains (eIF3b β-propeller domain and eIF3b-CTD segment) resolved here cannot be specified. However, 39 residues in this connector are adequate to span the distance as an unstructured linker.

Fitting of helices of eIF3a and eIF3c at the intersubunit interface

The eIF3a/eIF3c PCI dimer and eIF3b-CTD/eIF3i/eIF3g-NTD trimer make up more than half of the eIF3 complex in *S. cerevisiae*. Apart from the density corresponding to the eIF3b-CTD/eIF3i/eIF3g-NTD trimer, we observe three additional and differentiated regions of densities at the intersubunit interface of the 40S. One of these corresponds to a group of 5 helices that is clearly recognizable at this resolution near

h21/h24/h27. More density is located in contact eIF1 on the platform and seems to correspond to a globular domain of around 70-100 residues. Finally, there is density for two very long helices (clearly recognizable at this resolution) arranged as a coiled coil spanning the beta propeller of eIF3i and the density on the platform near eIF1.

Having assigned both the eIF3a/eIF3c PCI dimer and the eIF3b-CTD/eIF3i/eIF3g-NTD trimer, the C-terminus of eIF3a (496-964), NTD and β-propeller domain of eIF3b, N-terminus of eIF3c (1-250) and eIF3g-CTD are not accounted for. eIF3b can be ruled out because both the NTD and β-propeller domains of eIF3b are expected to be at the solvent interface (Liu et al., 2014; Erzberger et al., 2014). In fact, although we do not observe a distinct density for the whole β-propeller domain of eIF3b, we observe extra density at low resolution for part of the eIF2β-propeller domain in py48S-close complex at its expected position (see Movie S1). The eIF3g-CTD is known to bind near the entry channel (Cuchalova et al., 2010) and a solution structure of human eIF3g-CTD is available (PDB: 2CQ0). Based on this available structural data and its known location on the 40S, the eIF3g-CTD was also ruled out. Therefore, these unassigned densities should mainly correspond to segments of eIF3a and eIF3c.

Secondary structure prediction for the N-terminus of eIF3c (1-250) suggests a region of about 100 residues containing 5-6 helices of various lengths and at least another isolated helix close to the N-terminus, whereas the C-terminus of eIF3a (496-964) is predicted to consist of very long helices. Thus for the remaining unassigned density, we reason that the 5 helices near h21/h24/h27 belong to the 120-220 region of eIF3c based on this secondary structure prediction. This region (residues 120-220 of eIF3c) is predicted to have a group of 5 helices and the observed density clearly corresponds to a group of helices. The length of helices observed in the density also corresponds to what is expected according to the secondary structure prediction for 120-220 region of eIF3c. We modeled individual helices into the density but were unable to determine its topology, as the connecting loops are not clear and there is no side-chain information for an unambiguously sequence assignment. However, we reason that these densities correspond to the helices in the region of residues120-220 of eIF3c. Crosslinking data indicate eIF3c interacts with uS15 (Erzberger et al., 2014) and these helices can be easily linked to the PCI domain present on the solvent interface by a linker (~30 residues), which may interact with uS15 (Figure 6D) further supporting this assignment.

Based on the volume of density (equivalent to a globular domain of around 70-100 amino acids) in contact with eIF1 on the platform and its relative proximity to group of helices near h21/h24/h27 (tentatively assigned to region of residues120-220 of eIF3c), we suggest that it belongs to the N-terminal end of eIF3c (residues ~1-90), where it would form a direct contact with eIF1, in agreement with previous studies describing the most extreme N-terminal part of eIF3c as an interacting partner of eIF1 (Reibarkh et al., 2008; Erzberger et al., 2014). However, the density we observe is not sufficiently detailed so as to enable any model building. So, the assignment of this density in contact with eIF1 to N-terminal end of eIF3c (residues ~1-90) is primarily based on biochemical studies indicating N-terminal end of eIF3c interacts with eIF1. Secondly, its proximity to region of residues120-220 of eIF3c further supports it. Thirdly, we have ruled out more or less the rest of eIF3.

The remaining density for two long kinked helices spanning the β-propeller domain of eIF3i and the proposed eIF3c-NTD density near eIF1 can therefore only belong to eIF3a, in agreement with its secondary prediction of long stretches of helices, most likely to its CTD (from residue 760). The density for the long helices is reasonably clear and thus we assign it to the CTD of eIF3a. In fact previous studies have suggested the existence of a spectrin domain (bundle of three long helices) at the CTD of eIF3a functioning as the docking site for the formation of the a:b:i:g subcomplex (Dong et al., 2013). A recent study suggested direct interaction of the CTD of eIF3a with eIF3i and with the NTD of eIF3c (Politis et al., 2015), in agreement with the model proposed here. These helices account for more than 100 residues, and therefore our assignment (from residue 760) places the extreme C-terminal ~100 residues of eIF3a not modeled here in the vicinity of the TC, consistent with a known eIF3a-CTD interaction with eIF2 (Valasek et al., 2002).

Model Refinement and Validation

For an optimal fitting of the models into the EM density maps we used REFMAC v5.8, which has been modified to work with EM maps in a wide range of resolutions (Amunts et al., 2014; Brown et al., 2015). For all ribosomal and protein factors, ProSMART (Nicholls et al., 2012) was used to generate idealized helical restraints and hydrogen bond restraints for β-sheets. Base pair and stacking restraints for rRNA, $tRNA_i$ and mRNAs were generated using the program LIBG (Brown et al., 2015). All restraints were maintained throughout refinement. Refinement with restraints helps to preserve the correct geometry of previously known structures as well as reduce overfitting. Therefore, in this work at the present resolutions, REFMAC essentially fixes the small clashes and geometry that occur after separate rigid body fitting of individual domains.
Average Fourier shell correlation (FSC) was monitored during refinement. The result of the refinements was checked visually in Coot (Emsley et al., 2010). Final model was validated using MolProbity (Chen et al., 2010). To prevent overfitting, the global refinement and external restraints weights were carefully adjusted by cross-validation, as previously described (Brown et al., 2015; Amunts et al., 2014). Refinement statistics are given in Table 1.

Yeast strain constructions

To generate strains LMY61, LMY74, and LMY76, strain KAY18 (*MATα leu2-3 leu2-112 ura3-53 ino1 sui3Δ gcn2Δ* p921 (*SUI3$^+$, URA3*)) (Asano et al., 1999) was transformed to Leu$^+$ with low-copy (lc) *LEU2* plasmids harboring FLAG-tagged alleles *SUI3-FL* (plasmid YCpSUI3), *sui3-FL-S202A,K214A* (pLEM13), or *sui3-FL-F217A,Q221A* (pLEM15), respectively, and the resident *SUI3$^+$,URA3* plasmid p921 was evicted by selection on 5-fluoorotic acid (5-FOA) medium. To generate strains ATY49, ATY53, and ATY54, strain JCY03 (*MATa ura3-52 leu2-3 leu2-112 trp1Δ-63 his4-301(ACG) sui1Δ::hisG* p1200 (*SUI1$^+$*, *URA3*)) (Cheung et al., 2007) was transformed to Leu$^+$ with single-copy *LEU2* plasmids harboring *SUI1$^+$* (pJCB101), *sui1-F108A* (pAT117), or *sui1-F108D* (pAT118), respectively, and the resident *SUI1$^+$ URA3* plasmid (p1200) was evicted by selecting for growth on 5-FOA medium. Plasmids pLEM13 and pLEM15 were constructed from YCpSUI3 (Asano et al.,

1999) using the QuikChange site-directed mutagenesis system (Stratagene) according to the manufacturer's directions and the appropriate primers. Plasmids pAT117 and pAT118 were similarly constructed from pJCB101 (Martin-Marcos et al., 2011).

Yeast biochemical methods

Assays of β-galactosidase activities in whole-cell extracts (WCEs) were performed as described previously (Moehle and Hinnebusch, 1991) using transformants harboring the appropriate reporter plasmids, $HIS4_{AUG}$-*lacZ* (p367, $HIS4_{UUG}$-*lacZ* (p391) (Donahue and Cigan, 1988), or *GCN4-lacZ* (p180) (Hinnebusch, 1985). Transformants were cultured in appropriately supplemented synthetic dextrose minimal media (SD) at 30°C to an A_{600} of ~0.8. The same culture conditions were used for Western or coimmunoprecipitation analyses. WCEs for Western analysis were prepared by trichloroacetic acid extraction as previously described (Reid and Schatz, 1982), and immunoblot analysis was conducted as described (Nanda et al., 2009) using antibodies against Flag epitope (Sigma), eIF2Bε (Bushman et al., 1993), eIF1 (Valasek et al., 2004), or eIF3j (Valasek et al., 2001). Coimmunoprecipitations were conducted as previously described (Asano et al., 1999) and immunoblot analysis of immune complexes was conducted as above using antibodies against Flag epitope, eIF2γ (Hannig et al., 1993), eIF2α (Dever et al., 1995), and eIF2Bε.

SUPPLEMENTAL REFERENCES

Acker, M. G., Kolitz, S. E., Mitchell, S. F., Nanda, J. S., and Lorsch, J. R. (2007). Reconstitution of yeast translation initiation. Methods Enzymol *430*, 111-145.

Amunts, A., Brown, A., Bai, X. C., Llacer, J. L., Hussain, T., Emsley, P., Long, F., Murshudov, G., Scheres, S. H., and Ramakrishnan, V. (2014). Structure of the yeast mitochondrial large ribosomal subunit. Science *343*, 1485-1489.

Asano, K., Krishnamoorthy, T., Phan, L., Pavitt, G. D., and Hinnebusch, A. G. (1999). Conserved bipartite motifs in yeast eIF5 and eIF2Bepsilon, GTPase-activating and GDP-GTP exchange factors in translation initiation, mediate binding to their common substrate eIF2. EMBO J *18*, 1673-1688.

Aylett, C. H., Boehringer, D., Erzberger, J. P., Schaefer, T., and Ban, N. (2015). Structure of a Yeast 40S-eIF1-eIF1A-eIF3-eIF3j initiation complex. Nat Struct Mol Biol

Brown, A., Long, F., Nicholls, R. A., Toots, J., Emsley, P., and Murshudov, G. (2015). Tools for macromolecular model building and refinement into electron cryo-microscopy reconstructions. Acta Crystallogr D Biol Crystallogr *71*, 136-153.

Bushman, J. L., Foiani, M., Cigan, A. M., Paddon, C. J., and Hinnebusch, A. G. (1993). Guanine nucleotide exchange factor for eukaryotic translation initiation factor 2 in *Saccharomyces cerevisiae*: interactions between the essential subunits GCD2, GCD6, and GCD7 and the regulatory subunit GCN3. Mol Cell Biol *13*, 4618-4631.

Chen, V. B., Arendall, W. B., Headd, J. J., Keedy, D. A., Immormino, R. M., Kapral, G. J., Murray, L. W., Richardson, J. S., and Richardson, D. C. (2010). MolProbity:

all-atom structure validation for macromolecular crystallography. Acta Crystallogr D Biol Crystallogr *66*, 12-21.
Cheung, Y. N., Maag, D., Mitchell, S. F., Fekete, C. A., Algire, M. A., Takacs, J. E., Shirokikh, N., Pestova, T., Lorsch, J. R., and Hinnebusch, A. G. (2007). Dissociation of eIF1 from the 40S ribosomal subunit is a key step in start codon selection in vivo. Genes Dev *21*, 1217-1230.
Cuchalova, L., Kouba, T., Herrmannova, A., Danyi, I., Chiu, W. L., and Valasek, L. (2010). The RNA recognition motif of eukaryotic translation initiation factor 3g (eIF3g) is required for resumption of scanning of posttermination ribosomes for reinitiation on GCN4 and together with eIF3i stimulates linear scanning. Mol Cell Biol *30*, 4671-4686.
Dever, T. E., Yang, W., Astrom, S., Bystrom, A. S., and Hinnebusch, A. G. (1995). Modulation of tRNA(iMet), eIF-2, and eIF-2B expression shows that GCN4 translation is inversely coupled to the level of eIF-2.GTP.Met-tRNA(iMet) ternary complexes. Mol Cell Biol *15*, 6351-6363.
Donahue, T. F., and Cigan, A. M. (1988). Genetic selection for mutations that reduce or abolish ribosomal recognition of the HIS4 translational initiator region. Mol Cell Biol *8*, 2955-2963.
Dong, Z., Qi, J., Peng, H., Liu, J., and Zhang, J. T. (2013). Spectrin domain of eukaryotic initiation factor 3a is the docking site for formation of the a:b:i:g subcomplex. J Biol Chem *288*, 27951-27959.
Emsley, P., Lohkamp, B., Scott, W. G., and Cowtan, K. (2010). Features and development of Coot. Acta Crystallogr D Biol Crystallogr *66*, 486-501.
Erzberger, J. P., Stengel, F., Pellarin, R., Zhang, S., Schaefer, T., Aylett, C. H., Cimermancic, P., Boehringer, D., Sali, A., Aebersold, R., and Ban, N. (2014). Molecular architecture of the 40SeIF1eIF3 translation initiation complex. Cell *158*, 1123-1135.
Fernandez, I. S., Bai, X. C., Murshudov, G., Scheres, S. H., and Ramakrishnan, V. (2014). Initiation of translation by cricket paralysis virus IRES requires its translocation in the ribosome. Cell *157*, 823-831.
Hannig, E. M., Cigan, A. M., Freeman, B. A., and Kinzy, T. G. (1993). GCD11, a negative regulator of GCN4 expression, encodes the gamma subunit of eIF-2 in *Saccharomyces cerevisiae*. Mol Cell Biol *13*, 506-520.
Hashem, Y., des Georges, A., Dhote, V., Langlois, R., Liao, H. Y., Grassucci, R. A., Hellen, C. U., Pestova, T. V., and Frank, J. (2013). Structure of the mammalian ribosomal 43S preinitiation complex bound to the scanning factor DHX29. Cell *153*, 1108-1119.
Herrmannova, A., Daujotyte, D., Yang, J. C., Cuchalova, L., Gorrec, F., Wagner, S., Danyi, I., Lukavsky, P. J., and Valasek, L. S. (2012). Structural analysis of an eIF3 subcomplex reveals conserved interactions required for a stable and proper translation pre-initiation complex assembly. Nucleic Acids Res *40*, 2294-2311.
Hinnebusch, A. G. (1985). A hierarchy of trans-acting factors modulates translation of an activator of amino acid biosynthetic genes in *Saccharomyces cerevisiae*. Mol Cell Biol *5*, 2349-2360.
Hussain, T., Llacer, J. L., Fernandez, I. S., Munoz, A., Martin-Marcos, P., Savva, C. G., Lorsch, J. R., Hinnebusch, A. G., and Ramakrishnan, V. (2014). Structural changes enable start codon recognition by the eukaryotic translation initiation complex. Cell *159*, 597-607.

Liu, Y., Neumann, P., Kuhle, B., Monecke, T., Schell, S., Chari, A., and Ficner, R. (2014). Translation initiation factor eIF3b contains a nine-bladed beta-propeller and interacts with the 40S ribosomal subunit. Structure *22*, 923-930.
Martin-Marcos, P., Cheung, Y. N., and Hinnebusch, A. G. (2011). Functional elements in initiation factors 1, 1A, and 2beta discriminate against poor AUG context and non-AUG start codons. Mol Cell Biol *31*, 4814-4831.
Mitchell, S. F., Walker, S. E., Algire, M. A., Park, E. H., Hinnebusch, A. G., and Lorsch, J. R. (2010). The 5’-7-methylguanosine cap on eukaryotic mRNAs serves both to stimulate canonical translation initiation and to block an alternative pathway. Mol Cell *39*, 950-962.
Moehle, C. M., and Hinnebusch, A. G. (1991). Association of RAP1 binding sites with stringent control of ribosomal protein gene transcription in *Saccharomyces cerevisiae*. Mol Cell Biol *11*, 2723-2735.
Nanda, J. S., Cheung, Y. N., Takacs, J. E., Martin-Marcos, P., Saini, A. K., Hinnebusch, A. G., and Lorsch, J. R. (2009). eIF1 controls multiple steps in start codon recognition during eukaryotic translation initiation. J Mol Biol *394*, 268-285.
Nicholls, R. A., Long, F., and Murshudov, G. N. (2012). Low-resolution refinement tools in REFMAC5. Acta Crystallogr D Biol Crystallogr *68*, 404-417.
Pettersen, E. F., Goddard, T. D., Huang, C. C., Couch, G. S., Greenblatt, D. M., Meng, E. C., and Ferrin, T. E. (2004). UCSF Chimera--a visualization system for exploratory research and analysis. J Comput Chem *25*, 1605-1612.
Politis, A., Schmidt, C., Tjioe, E., Sandercock, A. M., Lasker, K., Gordiyenko, Y., Russel, D., Sali, A., and Robinson, C. V. (2015). Topological Models of Heteromeric Protein Assemblies from Mass Spectrometry: Application to the Yeast eIF3:eIF5 Complex. Chem Biol *22*, 117-128.
Reibarkh, M., Yamamoto, Y., Singh, C. R., del Rio, F., Fahmy, A., Lee, B., Luna, R. E., Ii, M., Wagner, G., and Asano, K. (2008). Eukaryotic initiation factor (eIF) 1 carries two distinct eIF5-binding faces important for multifactor assembly and AUG selection. J Biol Chem *283*, 1094-1103.
Reid, G. A., and Schatz, G. (1982). Import of proteins into mitochondria. Extramitochondrial pools and post-translational import of mitochondrial protein precursors in vivo. J Biol Chem *257*, 13062-13067.
Sokabe, M., Yao, M., Sakai, N., Toya, S., and Tanaka, I. (2006). Structure of archaeal translational initiation factor 2 betagamma-GDP reveals significant conformational change of the beta-subunit and switch 1 region. Proc Natl Acad Sci U S A *103*, 13016-13021.
Stolboushkina, E., Nikonov, S., Nikulin, A., Blasi, U., Manstein, D. J., Fedorov, R., Garber, M., and Nikonov, O. (2008). Crystal structure of the intact archaeal translation initiation factor 2 demonstrates very high conformational flexibility in the alpha- and beta-subunits. J Mol Biol *382*, 680-691.
Valasek, L., Nielsen, K. H., and Hinnebusch, A. G. (2002). Direct eIF2-eIF3 contact in the multifactor complex is important for translation initiation in vivo. EMBO J *21*, 5886-5898.
Valasek, L., Nielsen, K. H., Zhang, F., Fekete, C. A., and Hinnebusch, A. G. (2004). Interactions of eukaryotic translation initiation factor 3 (eIF3) subunit NIP1/c with eIF1 and eIF5 promote preinitiation complex assembly and regulate start codon selection. Mol Cell Biol *24*, 9437-9455.
Valasek, L., Phan, L., Schoenfeld, L. W., Valaskova, V., and Hinnebusch, A. G. (2001). Related eIF3 subunits TIF32 and HCR1 interact with an RNA recognition

motif in PRT1 required for eIF3 integrity and ribosome binding. EMBO J *20*, 891-904.
Yatime, L., Mechulam, Y., Blanquet, S., and Schmitt, E. (2007). Structure of an archaeal heterotrimeric initiation factor 2 reveals a nucleotide state between the GTP and the GDP states. Proc Natl Acad Sci U S A *104*, 18445-18450.
