# Supplementary figures and images for: Conformational Differences between Open and Closed States of the Eukaryotic Translation Initiation Complex
Source: Mol Cell. 2015 Aug 6;59(3):399–412. doi: 10.1016/j.molcel.2015.06.033 (PMC4534855; doi:10.1016/j.molcel.2015.06.033)

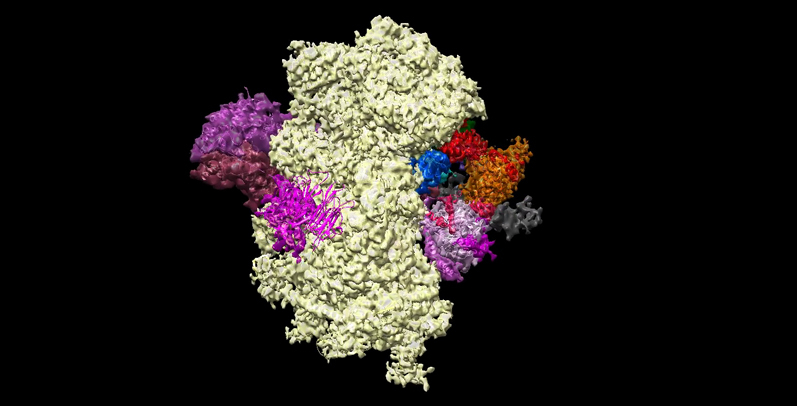

Supplement: Movie S1. py48S-Closed Complex — This movie shows a 360° rotation of the map of the py48S-closed complex, followed by the fitting of refined coordinates in the map. The fitting of ligands can also be observed with the maps shown as a transparent surface. The β-propeller of eIF3b shown at the solvent interface is modeled and not present in the py48S-closed complex. [file mmc2.jpg]

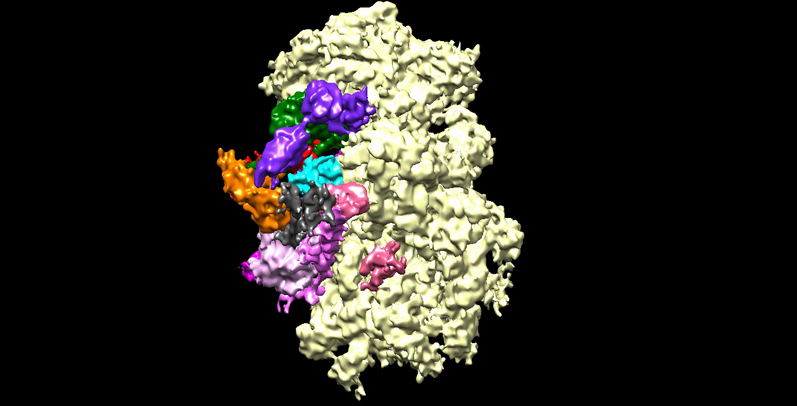

Supplement: Movie S2. py48S-Open Complex — This movie shows a 360° rotation of the map of py48S-open complex, followed by the fitting of refined coordinates in the map. The fitting of ligands can also be observed with maps shown as a transparent surface. [file mmc3.jpg]

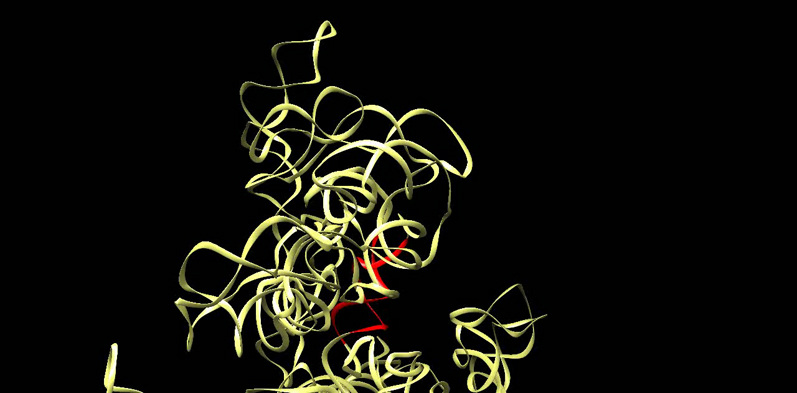

Supplement: Movie S3. Morphing of PICs: py48S-Open to py48S-Closed Complex — This movie shows the morphing of 18S rRNA in the py48S-open to the py48S-closed complex (colored cyan in the first frame). A short region (1,148–1,163; 1,615–1,627) in helix h28 is shown in red. Most ligands (except tRNAi and eIF2α) and all ribosomal proteins have been removed for clarity. The front view shows the upward movement of the head while no major conformational change is observed in the body. The change in position of tRNAi and eIF2α with the head movement can be clearly seen. [file mmc4.jpg]

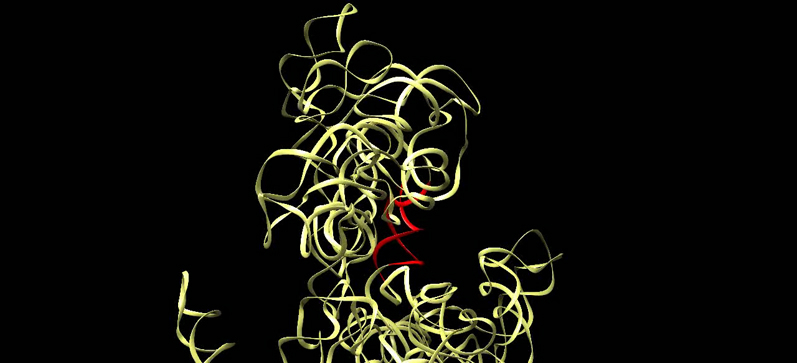

Supplement: Movie S4. Morphing of PICs: 40S•eIF1•eIF1A PIC to py48S-Closed Complex — This movie shows the morphing of 18S rRNA in the 40S•eIF1•eIF1A PIC to the py48S-closed complex (colored blue in the first frame). A short region (1,148–1,163; 1,615–1,627) in helix h28 is shown in red. All ligands and ribosomal proteins have been removed for clarity. The front view shows the rotation of the head while no major conformational change is observed in the body. [file mmc5.jpg]

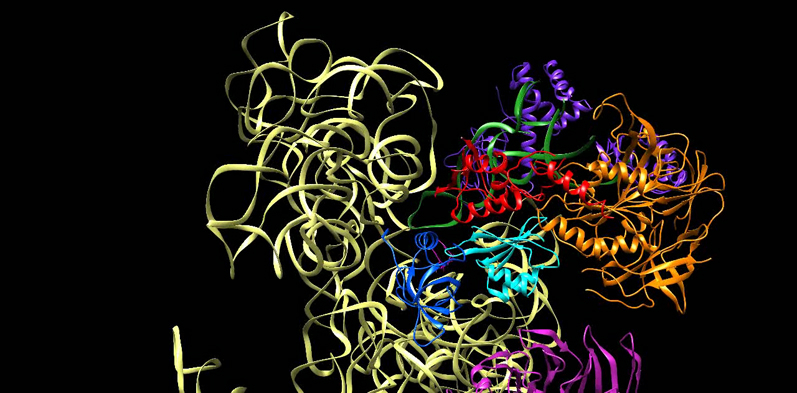

Supplement: Movie S5. Morphing of Ligands: py48S-Open to py48S-Closed Complex — This movie shows the morphing of the 18S rRNA and ligands in the py48S-open to the py48S-closed complex. The ligands are shown in color, as in Figure 1. Only the eIF3i subunit is shown for eIF3. All ribosomal proteins have been removed for clarity. This movie shows the conformational change that TC undergoes during the transition from the open to the closed state. [file mmc6.jpg]
