# Supplementary material for: Conformational Differences between Open and Closed States of the Eukaryotic Translation Initiation Complex
Source: Mol Cell. 2015 Aug 6;59(3):399–412. doi: 10.1016/j.molcel.2015.06.033 (PMC4534855; doi:10.1016/j.molcel.2015.06.033)
Supplement: Document S2. Article plus Supplemental Information [file mmc7.pdf]

Article

Molecular Cell

Conformational Differences between Open and Closed States of the Eukaryotic Translation Initiation Complex

Graphical Abstract

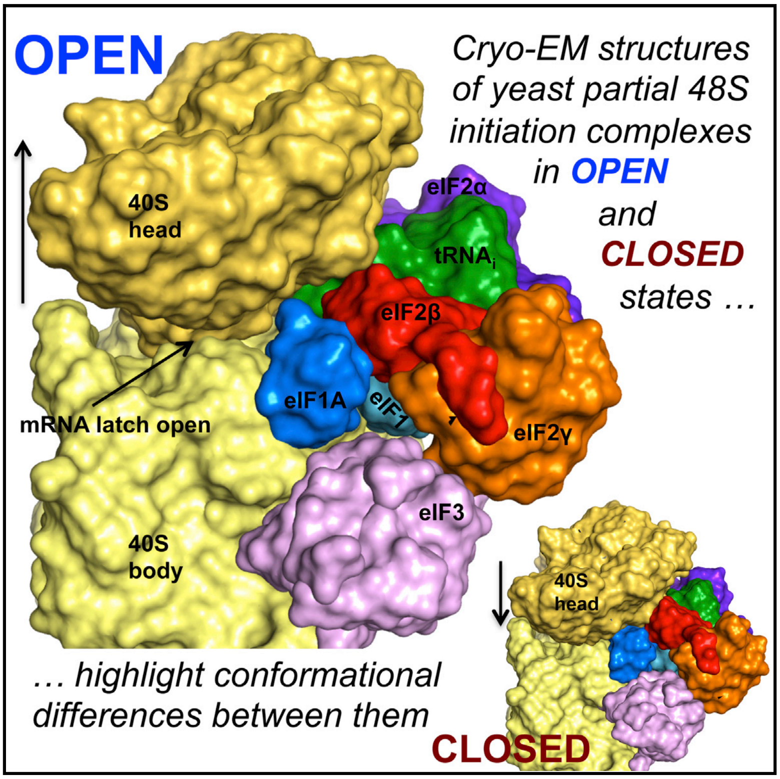

Authors

Jose L. Llácer, Tanweer Hussain, Laura Marler, ..., Jon R. Lorsch, Alan G. Hinnebusch, V. Ramakrishnan

Correspondence

ramak@mrc-lmb.cam.ac.uk

In Brief

The small ribosomal subunit latch has to open to allow mRNA loading. Llacer et al. report cryo-EM reconstructions of yeast translation pre-initiation complexes in both open and closed conformations, highlighting structural differences and elucidating key events in start codon recognition.

Highlights

- Structures of eukaryotic translation initiation complexes in open and closed states
- In the open complex the 40S head moves upward to open the mRNA entry channel latch
- Transition to closed state locks initiator tRNA in the P site base-paired with AUG
- The structures show how eIF3 contacts eIF2 and eIF1 on the 40S subunit interface

Accession Numbers

EMD-3047
EMD-3048
EMD-3049
EMD-3050
3JAM
3JAP
3JAQ

Llácer et al., 2015, Molecular Cell *59*, 399–412
August 6, 2015 ©2015 The Authors
http://dx.doi.org/10.1016/j.molcel.2015.06.033

Molecular Cell
Article

CellPress

Conformational Differences between Open and Closed States of the Eukaryotic Translation Initiation Complex

Jose L. Llácer,[1,4] Tanweer Hussain,[1,4] Laura Marler,[2] Colin Echeverría Aitken,[3] Anil Thakur,[2] Jon R. Lorsch,[3,5] Alan G. Hinnebusch,[2,5] and V. Ramakrishnan[1,*]
[1]MRC Laboratory of Molecular Biology, Cambridge CB2 0QH, UK
[2]Laboratory of Gene Regulation and Development, Eunice K. Shriver National Institute of Child Health and Human Development
[3]Laboratory on the Mechanism and Regulation of Protein Synthesis, Eunice K. Shriver National Institute of Child Health and Human Development
National Institutes of Health, Bethesda, MD 20892, USA
[4]Co-first author
[5]Co-senior author
*Correspondence: ramak@mrc-lmb.cam.ac.uk
http://dx.doi.org/10.1016/j.molcel.2015.06.033
This is an open access article under the CC BY license (http://creativecommons.org/licenses/by/4.0/).

SUMMARY

Translation initiation in eukaryotes begins with the formation of a pre-initiation complex (PIC) containing the 40S ribosomal subunit, eIF1, eIF1A, eIF3, ternary complex (eIF2-GTP-Met-tRNA$_i$), and eIF5. The PIC, in an open conformation, attaches to the 5′ end of the mRNA and scans to locate the start codon, whereupon it closes to arrest scanning. We present single particle cryo-electron microscopy (cryo-EM) reconstructions of 48S PICs from yeast in these open and closed states, at 6.0 Å and 4.9 Å, respectively. These reconstructions show eIF2β as well as a configuration of eIF3 that appears to encircle the 40S, occupying part of the subunit interface. Comparison of the complexes reveals a large conformational change in the 40S head from an open mRNA latch conformation to a closed one that constricts the mRNA entry channel and narrows the P site to enclose tRNA$_i$, thus elucidating key events in start codon recognition.

INTRODUCTION

In the first stage of eukaryotic translation initiation, the 40S ribosomal subunit and translation initiation factors eIF1, eIF1A, and eIF3 form a complex that facilitates loading of methionyl initiator tRNA (tRNA$_i$) onto the 40S subunit as a ternary complex (TC) with eIF2-GTP. eIF5, a GTPase activating protein (GAP) for eIF2, is thought to bind to this complex along with TC. The 43S preinitiation complex (PIC) thus formed binds to the capped 5′ end of mRNA in collaboration with the eIF4F complex, which consists of the cap-binding protein eIF4E, scaffolding protein eIF4G, and RNA helicase eIF4A. This 48S PIC, in an open conformation with tRNA$_i$ not fully engaged in the P site (P$_{OUT}$), then scans along mRNA. During the scanning process, GTP bound to eIF2 is hydrolyzed but the dissociated phosphate (P$_i$) is not released. Recognition of the start codon in the P site precipitates transition to a scanning-arrested, closed PIC with tRNA$_i$ accommodated in the P site (P$_{IN}$). This rearrangement triggers release of eIF1 and resultant dissociation of P$_i$ (Hinnebusch and Lorsch, 2012; Jackson et al., 2010; Voigts-Hoffmann et al., 2012; Hinnebusch, 2014).

One consequence of this process is that the mRNA cannot be threaded into the 40S subunit, because eIF4F is bound to the 5′ end, and hence must be loaded laterally into the mRNA channel. In the empty 40S subunit, this channel is closed because interactions between helix h34 in the head and h18 in the body form a latch (Passmore et al., 2007) that must open to allow initial loading of mRNA on the 40S subunit. It is thought that eIF1 and eIF1A promote the formation of an open conformation of the 40S subunit conducive to scanning (Pestova et al., 1998; Pestova and Kolupaeva, 2002). Moreover, a low-resolution cryo-EM reconstruction (22 Å) of a 40S•eIF1•eIF1A complex suggested that eIF1 and eIF1A unlock the latch, but the factors themselves could not be seen in this structure (Passmore et al., 2007). In contrast, all recent structures, including those with eIF1 and eIF1A, show the latch in a closed conformation (Ben-Shem et al., 2011; Rabl et al., 2011; Weisser et al., 2013; Hashem et al., 2013; Lomakin and Steitz, 2013; Hussain et al., 2014). A complete structure of the open conformation, in which the positions of the various factors are visible, would shed light on the mechanism of initial mRNA loading, scanning, and start codon recognition, as well as the roles played by each factor in these events.

Apart from capturing only the closed-latch conformation, previous structures are also incomplete in other ways. A recent 48S complex (py48S) from our groups shows details of the interaction of eIF1 and eIF1A with the ribosome and tRNA$_i$ during start-codon recognition, but it lacks density for most of the β subunit of eIF2 and all of eIF3 (Hussain et al., 2014). eIF3 is the largest and most complex of the eIFs, and is involved in nearly every aspect of initiation (Hinnebusch, 2006; Valásek, 2012).

CrossMark

Mammalian eIF3 consists of 13 subunits and has a molecular weight of ~800 kDa, whereas eIF3 in *Saccharomyces cerevisiae* and closely related yeasts is a ~395 kDa complex of five essential subunits (eIF3a, eIF3b, eIF3c, eIF3g, and eIF3i) and a sixth non-essential and substoichiometric subunit, eIF3j (Valásek et al., 2002; Hinnebusch, 2006). Crystal structures of parts of the various subunits of eIF3 from yeast have recently been solved (Hinnebusch, 2014; Erzberger et al., 2014), and a cryo-EM structure of 40S•eIF1•eIF1A•eIF3 is now available (Aylett et al., 2015). Domains of eIF3 were also modeled in a moderate resolution cryo-EM reconstruction of a partial mammalian 43S complex (hereafter pm43S) that lacked eIF1, eIF1A, eIF5, and mRNA (Hashem et al., 2013). All known structures of eIF3 bound to the 40S, as well as crosslinking studies, indicate binding on the solvent surface of the subunit (Siridechadilok et al., 2005; Hashem et al., 2013; Erzberger et al., 2014; Aylett et al., 2015). And yet eIF3 has been shown to interact with eIF1, TC, and eIF5, all of which bind to the intersubunit surface of the 40S (Hinnebusch, 2014). How eIF3 interacts with components bound near the P site while itself binding to the opposite face of the 40S subunit remains a key question in understanding the mechanism of translation initiation.

Here we have addressed these questions by determining a cryo-EM reconstruction at 6.0 Å resolution of a partial yeast 48S complex (with mRNA containing a near-cognate AUC in lieu of an AUG codon) that reveals an open, scanning-competent state with $tRNA_i$ not fully engaged in the P site, hereafter referred to as "py48S-open." A second complex elucidated at 4.9 Å resolution contains mRNA with an AUG codon that presents a closed conformation with the latch closed, entry channel constricted, and $tRNA_i$ locked into the P_{IN} state, hereafter referred to as "py48S-closed." These structures show clear density for eIF1, eIF1A, mRNA, the entire TC, including previously unseen eIF2β, as well as segments of eIF3. The py48S-open complex shows an open latch conformation, expanded entry channel and widened P site, suggesting mechanisms for loading and scanning of mRNA and is markedly different from a pm48S reconstructed at 11.6 Å (Hashem et al., 2013). Comparison with py48S-closed illuminates the structural changes that occur within the 40S subunit, TC, and other eIFs during the transition from the open to closed state of the PIC that should arrest scanning and lock $tRNA_i$ into the P site and highlights the importance of the 40S head conformation and roles of eIFs in stabilizing the two states. We also observe portions of eIF3 on the subunit-joining interface of the 40S subunit in both complexes, showing how eIF3 can contact TC and eIF1 near the P site while remaining bound to the solvent face of the 40S subunit.

RESULTS

Formation and Overview of Structures

Yeast py48S-closed was assembled as described previously (Hussain et al., 2014) using an unstructured, uncapped mRNA with an AUG codon; 40S subunits from yeast *Kluyveromyces lactis*; and factors eIF1, eIF1A, eIF3, eIF5, and TC from *S. cerevisiae*. We used the U31:A39 variant of $tRNA_i$ that stabilizes the P_{IN} state (Dong et al., 2014) to promote formation of the 48S in the P_{IN} state, but wild-type eIF2 rather than the Sui3-2 variant of eIF2 (Donahue et al., 1988) used previously (Hussain et al., 2014). We similarly combined 40S with eIF1, eIF1A, eIF3, TC, and mRNA to generate the py48S-open complex. However, to shift the equilibrium from the P_{IN} state, wild-type $tRNA_i$ was used, the AUG codon was replaced with near-cognate AUC, eIF5 was omitted as it shifts the equilibrium toward P_{IN} state (Maag et al., 2006; Nanda et al., 2013), and recombinant eIF3 expressed in *E. coli* (and thus free of eIF5) was used instead of native eIF3 (see Supplemental Experimental Procedures).

The structures for py48S-closed and py48S-open were determined to overall resolutions of 4.9 Å and 6.0 Å, respectively (Figures 1, S1, and S2; Table 1; Movies S1 and S2). Like py48S (Hussain et al., 2014) the local resolution and density is best for the core of 40S and ligands directly attached to it (Figure S3 and Table S1). Large data sets and extensive 3D classification were required to obtain PIC classes with eIF1, eIF1A, eIF3, TC (including eIF2β), and mRNA all bound (Figure S1; see Supplemental Experimental Procedures). These classes comprised 1.8% of the total for py48S-closed and 1.0% for py48S-open. The majority of particles were deficient in one or more factors as a result of the characteristic dissociation of factors on the EM grid (Figure S1; See Supplemental Experimental Procedures).

We observe density in both complexes for subunits of eIF3, as well as for eIF1, eIF1A, mRNA, and all three subunits of TC. Interestingly, density for components of eIF3 appears in the subunit-joining interface, where it interacts with other eIFs. In the solvent interface, the density for eIF3 density is weaker, especially for py48S-open (discussed later). We observe density for eIF2β in both complexes (Figures 1 and S2). The py48S-closed (4.9 Å) is globally similar to our previously reported py48S (4.0 Å) (Hussain et al., 2014) (RMSD of 0.86 Å for 33,178 atoms of 18S) except for the additional densities for eIF2β and eIF3. The use of large data sets may have allowed us to isolate a class that includes eIF2β and eIF3, which would have been missed earlier in py48S. However, it is also possible that the use of WT eIF2 instead of Sui3-2 variant may have resulted in observation of these eIFs.

The large data set and extensive classification also enabled us to determine structures of a 40S•eIF1•eIF1A complex at 3.5 Å resolution (Figures S1 and S4A) and a 40S•eIF1•eIF1A•TC complex, representing a partial 43S PIC (py43S), at 15 Å resolution (Figures S1 and S4B), which has a conformation similar to pm43S (Hashem et al., 2013).

Altered Conformation of the 40S Head in the Open Conformation of py48S

Whereas the orientations of the 40S body are similar in both py48S-open and py48S-closed, the two structures differ in the conformation of the 40S head (Figure 2; Movie S3). While the orientation of the head in py48S-closed is similar to that in py48S reported earlier (Hussain et al., 2014), in py48S-open, there is a remarkable upward movement of the head away from the body (Figures 2A and 2B), in a different direction from that distinguishing the 40S•eIF1•eIF1A complex from empty 40S or py48S (Movie S4). This head movement from py48S-closed to py48S-open is accompanied by a 7–8 Å change in the pitch of h28 (Figures 2C and 2D) and a repositioning of the

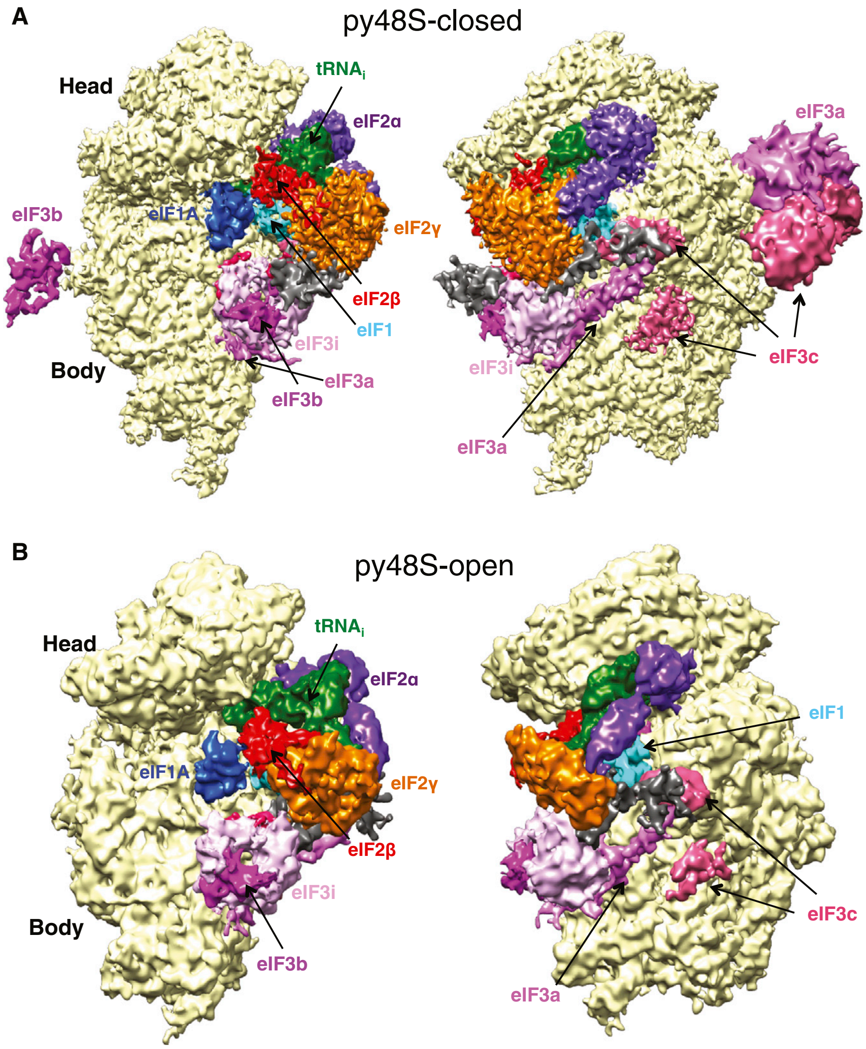

Figure 1. Cryo-EM Maps of Eukaryotic 48S PICs

(A) Two views of py48S-closed.

(B) Two views of py48S-open. Density for eIF3 is Gaussian-filtered. Unassigned density is in dark gray.

See also Figures S1–S4; Table S1; and Movies S1 and S2.

β-hairpin of uS5 that contacts h28 (Figure 2C). This helix constitutes the "neck" of the 40S that connects the head to the body, and is compressed in py48S-closed but relaxed in py48S-open. Interestingly, mutations in this region of h28 (A1151, A1152, and U1627; *S. cerevisiae* numbering throughout the manuscript) lead to a Gcd$^-$ phenotype, indicating less stable TC binding to the PIC (Dong et al., 2008).

This conformation of the head in py48S-open throws open the mRNA channel latch and widens the channel, particularly at the entry channel side near the A site (Figure 2B). Helix 34 and associated elements move away from h18 to open the latch. The py48S-open structure reveals both the upward shift of the 40S head and open-latch conformation, thus providing insights into changes involved during key steps of initiation.

The 40S head is also moved upward in py48S-open compared to pm43S (Hashem et al., 2013) (Figure S4C). In contrast, the head conformations of the pm43S and py48S-closed are more similar (Figure S4D). Note that pm43S lacks mRNA, and densities for eIF1, eIF1A, and eIF2β were missing in the reconstruction. In py43S (which most closely mimics pm43S), the latch is again closed and the head orientation is almost identical to that of pm43S (Figure S4E). The positions of TC in pm43S and py43S are also similar (Figure S4E); however, eIF1 and eIF1A densities are clearly seen in our py43S map (Figure S4B). Thus, the presence of TC with eIF3 in pm43S, or TC with eIF1 and eIF1A in py43S here, both lead to a similar orientation of the head with a closed latch and may thus represent a state prior to the binding of mRNA.

A Widened P Site and Altered Orientation of tRNA$_i$ in py48S-Open

Interestingly, the P site in py48S-open is widened compared to that of py48S-closed (Figures 3A and 3B), lacking interactions between tRNA$_i$ and the 40S body that occur in the closed complex (Figures 3A and 3B). As a result of the altered head position in py48S-open, the tRNA$_i$ adopts a previously unobserved modified P/I orientation, which we call sP/I for "scanning P/I" conformation (Figure S5A; Movie S3). Nevertheless, the tRNA$_i$ maintains the same contacts with the head in both complexes, which ensures that the conserved GC base pairs in the anticodon stem-loop (ASL) of tRNA$_i$ are recognized by rRNA residues G1575 and A1576 in both py48S-closed and py48S-open (Figures 3A and 3B).

While the tip of the ASL of tRNA$_i$ is deep within the P site in both the open and closed complexes, it is displaced laterally from the body by ~7 Å in the py48S-open (Figure 3C), owing to both the widened P site and altered head orientation. Superimposing the head in the open and closed structures shows that the positions of the ASL relative to the head are very similar in both (Figures 3D and 3E); the ASL essentially moves with the head during the open-to-closed transition. In contrast, in pm43S (Hashem et al., 2013), the tip of the ASL is not deep in the P site (Hussain et al., 2014) and thus does not track with the head movement.

Although the ASL tracks with the head as it moves from the open to closed conformation, the positions of the tRNA$_i$ acceptor arm in both structures are superimposable relative to the body (Figure 3C). As the ASL remains in contact with the head, it must bend in going from the open to closed state, which allows the tRNA$_i$ to maintain codon-anticodon interaction during the transition that would otherwise clash with the 40S body

Table 1. Refinement and Model Statistics

	py48S-Open	py48S-Closed	40S•eIF1•eIF1A
Data Collection			
Particles	4,547	21,401	86,055
Pixel size (Å)	1.34	1.34	1.34
Defocus range (μm)	1.6–4.0	1.6–4.0	1.6–4.0
Voltage (kV)	300	300	300
Electron dose (e^{-} $Å^{-2}$)	28	28	28
Model Composition			
Non-hydrogen atoms	89,774	98,371	77,850
Protein residues	6,413	7,446	5,056
RNA bases	1,855	1,869	1,780
Refinement			
Resolution used for refinement (Å)	6.20	5.00	3.50
Map sharpening B-factor (Å)	−20	−119	−81
Average B-factor (Å)	NA	NA	89
R factor #	34.6	38.4	27.7
Fourier Shell Correlation (FSC)*	0.69	0.70	0.85
Rms deviations			
Bonds (Å)	0.006	0.007	0.006
Angles (°)	1.00	1.20	1.16
Validation (proteins)			
Molprobity score	2.43 (99th percentile)	2.94 (90th percentile)	2.87 (92th percentile)
Clashscore, all atoms	4.09 (100th percentile)	9.79 (97th percentile)	7.63 (97th percentile)
Good rotamers (%)	93.4	88.4	88.2
Ramachandran plot			
Favored (%)	87.3	86.8	86.0
Outliers (%)	3.3	3.3	3.8
Validation (RNA)			
Correct sugar puckers(%)	96.5	95.5	96.8
Good backbone conformations(%)	55.6	50	62.5

(Figure S5B and Movie S3). The bent ASL in py48S-close is similar to that observed earlier in py48S (Hussain et al., 2014) and pm48S (Lomakin and Steitz, 2013), and allows base pairing with the codon in P_{IN} state.

Path of mRNA in the Two Structures

In our previous py48S complex (Hussain et al., 2014), mRNA was observed throughout the mRNA channel. In contrast, the py48S-closed here shows density for mRNA mainly in the exit channel (Figure S2A). Strikingly, in py48S-open, the mRNA entry channel is widened, which, along with the open latch, produces a conformation that should allow single-stranded mRNA to be slotted directly into the mRNA-binding channel (Figures 2A and 2B). We observe discontinuous densities for mRNA, mainly in the P and E sites of py48S-open, including density consistent with base pairing between the A and U of the AUC codon and the U and A of the anticodon (Figure S2B, right). Incidentally, the mRNA also has another AUC codon, but because it is only 3 nt away from 5′ end, it appears not to be involved in recognition. The P-site codon has moved in concert with the ASL and 40S head such that base pairing is maintained in py48S-open, despite the $tRNA_i$ not yet having been fully accommodated in the eP/I′ configuration (Figures 3E and S5B). The minimal density for mRNA in py48S-open suggests that, probably as a result of the widened mRNA channel, the mRNA has minimal contact with the ribosome apart from base pairing with the anticodon, which should facilitate scanning. Upon AUG recognition, head repositioning stabilized by interaction of the N-terminal tail (NTT) of eIF1A with the codon:anticodon duplex (discussed below), and latch closure, will narrow the entry channel to fix the mRNA and arrest scanning.

Changes in eIF1and eIF1A between the Closed and Open States of py48S

We observe eIF1and eIF1A clearly in both the open and closed PICs (Figures 1, S2A, and S2B), but with marked changes in their conformations between the two states. The overall conformation of eIF1A is similar in the two complexes, but the NTT of eIF1A interacts with the anticodon-codon duplex only in py48S-closed, as in our previous py48S without eIF3 (Hussain et al., 2014)—consistent with its role in promoting recognition of a cognate start codon (Figure 4A). In contrast, the NTT is disordered in py48S-open (Figure 4A), and in the 40S•eIF1•eIF1A complex (Figure S4A), as in all other reported PICs (Weisser et al., 2013; Lomakin and Steitz, 2013; Hussain et al., 2014). There is no distinct density for the C-terminal tail (CTT) of eIF1A in any of the closed complexes as expected from previous hydroxyl radical cleavage studies that show that eIF1A-CTT interferes with the P-site tRNA (Yu et al., 2009; Nanda et al., 2013). The CTT of eIF1A is also not modeled in py48S-open because of lack of clear unambiguous density.

The overall position of eIF1 in the two complexes is also similar (Figure 4B), but β-hairpins 1 and 2 of eIF1 are positioned differently. Their orientations in py48S-open resemble those observed in the 40S•eIF1•eIF1A complex, with no steric clash with $tRNA_i$. In py48S-closed, however, the two β-hairpins are displaced to avoid a clash with the now-accommodated $tRNA_i$ (Figure 4B), as seen earlier in py48S (Hussain et al., 2014). An interesting feature in py48S-open is the interaction of β-hairpin 1 with the AUC codon in the P site (Figure 3E). As we observe a similar interaction with the AUG codon in py48S-closed (Figure S5B), the conformation of β-hairpin 1 changes between the two states to follow the $tRNA_i$ ASL and P-site codon as they are adjusted during the open-to-closed transition, preserving this interaction (Figure 4B).

eIF2β Links TC to the 40S Head and Body in the py48S-Open Complex

We observe density for all three subunits of eIF2 in the PIC, including eIF2β (Figures S2A and S2B). eIF2 is bound primarily to the 40S head in py48S-closed (Figure 1A) and, as in py48S (Hussain et al., 2014), eIF2α-D1 (domain 1) and the $tRNA_i$ ASL together attach TC to the head (Movie S3). During the transition from py48S-open to py48S-closed, eIF2α-D1 rotates slightly,

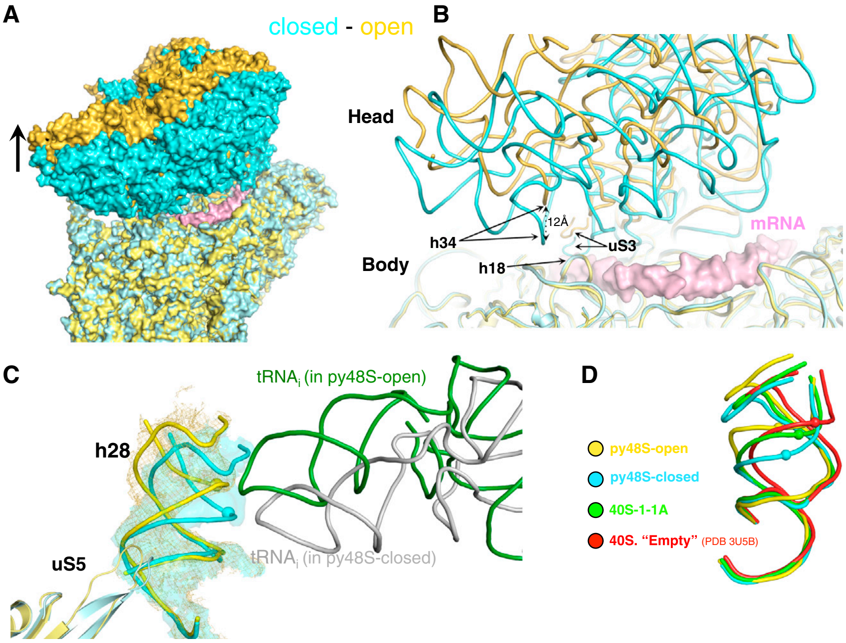

Figure 2. Distinct Position of the 40S Head Widens the mRNA Entry Channel and Opens the Latch in the Open Complex

(A) Front view of superposition of py48S-open (yellow) and py48S-closed (cyan), showing mRNA (pink) from py48S (Hussain et al., 2014) to highlight the complete mRNA channel.

(B) Superposition of refined models of py48S-open (yellow) and py48S-closed (cyan), indicating elements forming the latch.

(C) h28 in py48S-open (yellow) and py48S-closed (cyan) based on superposition of the two complexes, viewed from the A site. $tRNA_i$ and uS5 for the two complexes are also shown.

(D) h28 in empty 40S (red, PDB: 3U5B), 40S•eIF1•eIF1A (green), py48-open (yellow), and py48S-closed (cyan) complexes. Equivalent atoms in h28 are shown as spheres.

See also Figures S4 and S2 and Movies S3 and S4.

thus avoiding a clash with the 40S body (Movie S3). This positions the loop containing Arg55 and Arg57 to enable their interactions with mRNA nucleotides −2 to −3 in the E site (Figures S6A and S6B), as observed in the py48S. Another consequence of this rotation is that eIF2α-D2 and the D-and T-arms of the $tRNA_i$ are positioned closer to the head in py48S-closed compared to py48S-open (Figures 3A and 3B). Moreover, eIF2α-D2 moves in relation to eIF2α-D1 and interacts closely with the D- and T-loops of $tRNA_i$ (Figure 5A; Movie S3). The third domain eIF2α-D3 moves with respect to the acceptor arm of the $tRNA_i$ (Figures 5A; Movie S5).

We could model most of eIF2β in both complexes (Figures 1 and S2), except for the disordered N-terminal residues 1–125 and the last 20 C-terminal residues of the protein. As in previous archeal βγ complexes (Stolboushkina et al., 2008; Sokabe et al., 2006), eIF2β is tightly attached to eIF2γ by its N-terminal helix α1 (Figures S2A and S2B). Notably, in py48S-open, the helix-turn-helix (HTH) domain of eIF2β binds to eIF1 and eIF1A on the 40S body and to $tRNA_i$ bound to the 40S head, bridging the 40S head and 40S body, without direct interactions with the 40S itself (Figure 5B, upper). These interactions likely stabilize the open conformation during scanning in the absence of a complete codon-anticodon duplex.

During rearrangement to the closed complex, the eIF2β HTH domain is positioned away from eIF1 and eIF1A (Figure 5B, lower) and binds to elements of the 40S head. Because of its altered position, the HTH domain also makes contacts with the $tRNA_i$ that are distinct from those occurring in py48S-open (Figure 5B, upper). The position of eIF2β in py48S-open would result in a clash with both eIF1 and eIF1A in py48S-closed due to inward movement of the 40S head and body (Figure S6C).

The zinc-binding domain (ZBD) of eIF2β is positioned close to the GTP binding pocket of eIF2γ in both complexes (Figure 5C), similar to its position in some archaeal βγ complexes (Stolboushkina et al., 2008), although the ZBD itself was disordered in that structure. Because eIF1 is present in both complexes and eIF1 dissociation from the PIC is a prerequisite for P_i release from eIF2•GDP•P_i on AUG recognition (Algire et al., 2005), it remains unclear whether changes in the interaction of the eIF2β-ZBD with the eIF2γ GTPase center on eIF1 release are involved in P_i release.

In our previously reported py48S structure (Hussain et al., 2014), density for eIF2β was largely absent. This may be because we used the Sui3-2 variant of eIF2 harboring the S264Y substitution in eIF2β to stabilize the closed PIC conformation. Interestingly, S264Y maps to the interface between the eIF2β-ZBD and the eIF2γ GTPase center (Figure 5C). As such, it might destabilize this interface and increase mobility of the eIF2β-ZBD, which could disrupt the interactions of eIF2β with eIF1 and eIF1A that are unique to py48S-open (Figure 5B). This might explain how the Sui3-2 mutant stabilizes TC binding in the closed conformation (Martin-Marcos et al., 2014). Sui3-2 may specifically destabilize the open conformation and shift the equilibrium toward the closed conformation, thus accounting for its increased utilization of near-cognate start codons in vivo (Donahue et al., 1988).

Interestingly, a superposition of eIF2βγ using eIF2γ as a reference shows that the relative orientation of these two subunits is the same in both py48S-open and py48S-closed (Figure S6D). Hence, eIF2βγ, along with eIF2α-D3, alters its position relative to $tRNA_i$ and domains 1 and 2 of eIF2α in a concerted manner between the two complexes (Figures 5D and S6E; Movie S5).

Initiation Accuracy In Vivo Is Reduced by Disrupting eIF2β Contacts with $tRNA_i$ or eIF1 that Occur Only in py48S-Open

The fact that eIF2β makes interactions with the $tRNA_i$ ASL and eIF1 in py48S-open that are missing or altered in py48S-closed (Figure 6A) suggests that these contacts specifically stabilize the open, scanning conformation of the PIC. If so, then substituting residues at these contacts should increase the frequency of initiation at UUG codons by facilitating rearrangement from the

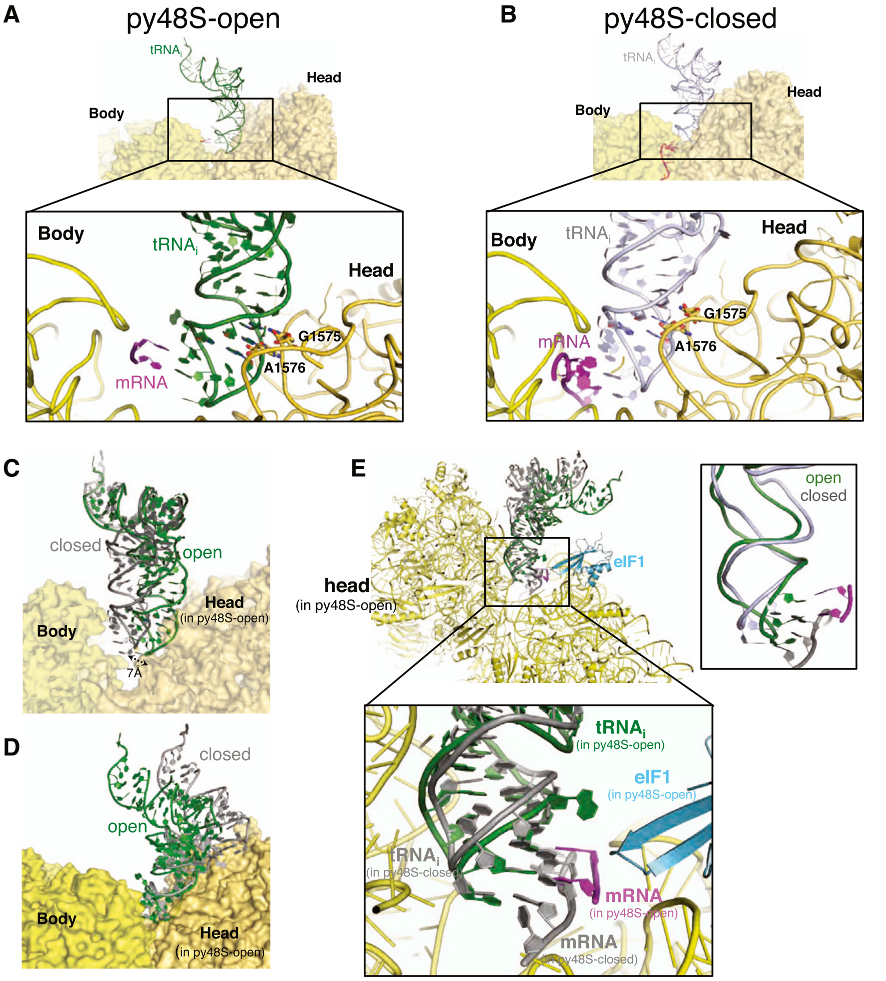

Figure 3. tRNA$_i$ Is Not Engaged with P-Site Elements of the 40S Body in the Open Complex

(A) tRNA$_i$ in py48S-open, viewed from E site. The body and head of 40S are shown in lighter and darker shades of yellow. The zoomed view shows mRNA at the P site and recognition of conserved GC base pairs in ASL by rRNA bases. For clarity, 40S proteins and other factors are not shown.

(B) The tRNA$_i$ in py48S-closed viewed as in (A).

(C) Superposition of the 40S body reveals distinct locations of tRNA$_i$ in the P site of py48S-open (green) and py48S-closed (gray). The body and head of py48S-open complex are shown. The two ASLs are separated by about 7 Å in the P site.

(D) Superposition of the 40S head of py48S-open (green) and py48S-closed (gray).

(E) Superposition of two complexes as in (D), viewed from the A site. The mRNA of py48S-closed is in gray. Inset shows the superposition of the two positions of tRNA$_i$ and interacting mRNA codon.

See also Figure S5.

open to closed conformations in the absence of a perfect start codon:anticodon duplex in the P site. Supporting this prediction, substitutions of eIF2β Phe217/Gln221 and eIF1 Phe108, residues juxtaposed at the eIF2β/eIF1 interface in py48S-open (Figure 6A), substantially increase the UUG:AUG expression ratio for matched *HIS4-lacZ* fusions differing only in the start codon (Figure 6B). The eIF1-F108A/F108D substitutions also increase eIF1 abundance (Figure 6D), an established indicator of relaxed discrimination against the suboptimal context of the AUG start codon of the (*SUI1*) mRNA encoding eIF1 (Martin-Marcos et al., 2011). Thus, eIF1-F108A/F108D facilitate initiation for both a near-cognate start codon and an AUG in poor context. eIF2β substitutions S202A/K214A also increase UUG initiation (Figure 6B) and, consistent with impaired eIF2β interaction with tRNA$_i$, derepress expression of a *GCN4-lacZ* reporter (Figure 6C), an in vivo indicator of reduced TC assembly or binding to the scanning PIC (Hinnebusch, 2005). None of the eIF2β substitutions significantly affect eIF2β abundance (Figure 6E) or its assembly with eIF2α/eIF2γ in the eIF2 complex (Figure 6F).

Placement of eIF3 subunits on both faces of the PIC

We observe density for eIF3 in both py48S-open and py48S-closed (Figures 1A and 1B). At this resolution, we can identify helices, place domains of known structure into the density, and make tentative assignments of previously unobserved segments of eIF3, based on secondary structure predictions (Figures S2C, S2D, and S2E; Supplemental Experimental Procedures). Because the densities attributable to eIF3 on the subunit interface are similar in both complexes, we describe the appearance of eIF3 only in the higher resolution py48S-closed (Figure 7).

The two PCI domains of the eIF3a/eIF3c heterodimeric core bind near the left shoulder of the 40S solvent face (Figure 7A; Movie S1), as in the yeast 40S•eIF1•eIF1A•eIF3 structure (Aylett et al., 2015). However, in py48S-closed, the PCI domains are displaced laterally, which may reflect a conformational change in eIF3 during different steps of initiation (Figure 7B). We modeled the eIF3b β-propeller domain with the help of the 40S•eIF1•eIF1A•eIF3 structure (Figures 7A and 7C) as we detect only a part of it in the density (Figures 1A and 1B). In py48S-open, weak densities of the PCI domains and eIF3b β-propeller appear only in low-resolution filtered map contoured at lower threshold (not shown) and were not modeled. It is not clear whether the much weaker density for these regions of eIF3 in py48S-open is due to the lower quality of the data (fewer particles and lower resolution) or inherently greater flexibility or lower occupancy of these domains in py48S-open.

Remarkably, we see additional density for eIF3 in both complexes, at the subunit interface near h44, uS12, and eIF2γ (Figures 1A and 1B). Based on its characteristic shape and dimensions, we assigned this density to the trimeric subcomplex composed of the β-propeller domain of eIF3i, ~30 residues from the eIF3b C-terminal domain (CTD) and ~50 residues from the N-terminal domain (NTD) of eIF3g (Erzberger et al., 2014)

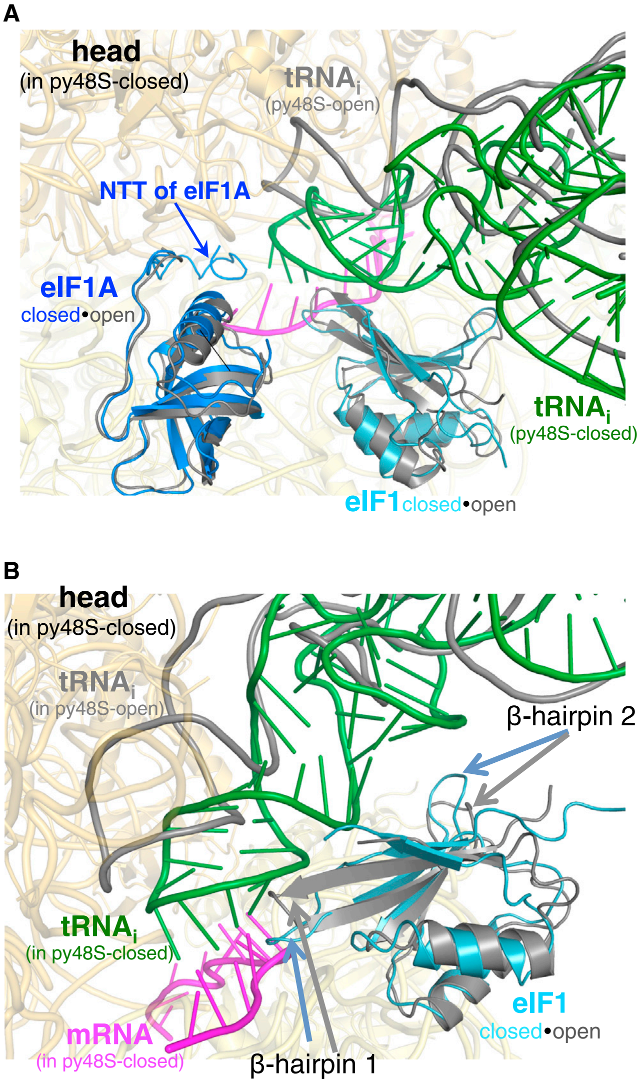

Figure 4. Contacts of eIF1A-NTT and eIF1 with tRNA$_i$ Restricted to the Closed Complex

(A) Superposition of the open and closed complexes with the ligands of py48S-closed shown in color while those of py48S-open are in gray. Only the 40S of py48S-closed is shown (yellow). The zoomed view shows the NTT of eIF1A in the two complexes.

(B) Superposition of the open and closed complexes as in (A).

(Figure S2D; Movie S1; See Supplemental Experimental Procedures). eIF3i is positioned in the vicinity of eIF2γ, and the NTD of eIF3g may directly contact eIF2γ in the py48S complexes (Figures 7C and 7D). eIF3i was earlier predicted to bind in two possible positions at the solvent-exposed surface of the 40S subunit, either above or below the β-propeller domain of eIF3b (Erzberger et al., 2014). Neither of these configurations is consistent with the density we observe, suggesting that eIF3 undergoes a significant rearrangement undetected by prior models, perhaps on binding mRNA. This position, in which eIF3i holds eIF3g against eIF2γ and by consequence promotes the intricate TC/eIF1A/eIF1 interaction network, might explain the suppression of eIF3i and eIF3g mutant phenotypes by over-expression of eIF1 or eIF1A and the formation of aberrant 43S complexes observed in the absence of these subunits (Cuchalová et al., 2010; Herrmannová et al., 2012). This configuration also places eIF3i and eIF3g along the path of mRNA through the decoding center, consistent with the scanning defects observed for mutants of these subunits (Cuchalová et al., 2010).

We also observe density in both complexes for a cluster of five α helices in a pocket formed by h11, h24, h27, h44, and uS15 that has been putatively assigned to a predicted helix-rich segment in the eIF3c-NTD (Figure 7D; See Supplemental Experimental Procedures), which we connect to the eIF3c PCI domain by a ~30-residue flexible linker. The remaining N-terminal residues of eIF3c likely emanate from the five-helix cluster and mediate the known interaction of eIF3c-NTD with eIF1 (Fletcher et al., 1999; Asano et al., 2000), which appears to enhance the stability of eIF1 within the PIC (Valásek et al., 2004; Karásková et al., 2012). We therefore tentatively assigned the globular density in contact with eIF1 as the N-terminal region of the eIF3c-NTD (1–90 residues), with a single α-helix near h24 modeled in the density (Figure 7D; see Supplemental Experimental Procedures). This moiety approaches the surface of eIF1 identified as an eIF3c-binding surface (Reibarkh et al., 2008).

Closer to the subunit interface, we detect density in both complexes for an extended helical region spanning h14, h44, and h27 (Figures 1, S2E, and 7D). These helices have been provisionally assigned to a region in the CTD of eIF3a (See Supplemental Experimental Procedures) predicted to have long helices (Dong et al., 2013), and they bridge the β-propeller domain of eIF3i and the putative eIF3c-NTD moiety near eIF1. This assignment places the extreme C-terminal ~100 residues of eIF3a not modeled here in the vicinity of the TC, consistent with a known eIF3a-CTD interaction with eIF2 (Valásek et al., 2002). We suggest that the unassigned central portion of eIF3a projects away from the eIF3a PCI domain near the exit channel on the 40S solvent face subunit and passes through the mRNA entry channel and across the intersubunit face, connecting with the extended helices assigned to the eIF3a-CTD (Figures 7A and 7C). As these extended helices approach the eIF3c-NTD (Figure 7D), it appears that eIF3a and eIF3c together encircle the PIC. This proposal is consistent with previous observations that regions of the eIF3a CTD interact with 40S components at the mRNA entry channel (Valásek et al., 2003; Chiu et al., 2010) as well as structural models for the yeast 40S•eIF1•eIF3 complex (Erzberger et al., 2014) and the pm43S (Hashem et al., 2013), and chemical and enzymatic footprinting data (Pisarev et al., 2008), all of which place eIF3 components at both the exit and entry channels on the solvent side of the 40S subunit.

DISCUSSION

Our py48S-open and py48S-closed structures contain density for eIF1, eIF1A, all three subunits of eIF2 bound to Met-tRNA$_i$, mRNA, and various components of eIF3. Although both structures are at lower resolution than the previously reported py48S (Hussain et al., 2014), this is likely to be the result of the small fraction of the particles in these classes. Despite this, the

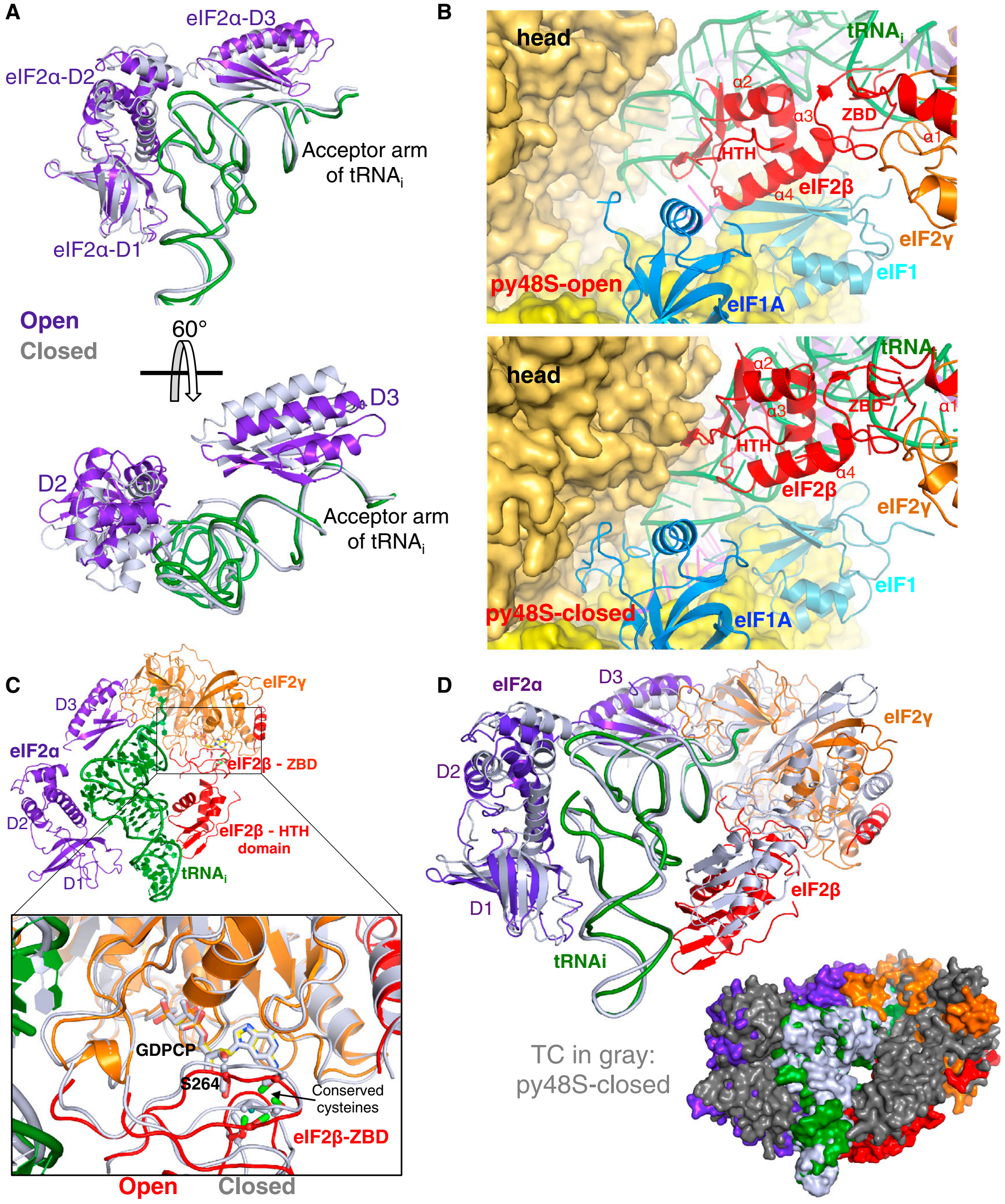

Figure 5. Distinctive Interactions of eIF2β with eIF1, eIF1A, and tRNA$_i$ Occlude the mRNA Channel in py48S-Open

(A) Conformational changes in eIF2α based on superposition of the TC coordinates using tRNA$_i$ as the reference. The eIF2α and tRNA$_i$ of py48S-open are shown in color and those of py48S-closed are in gray.

(B) Position of eIF2β with respect to tRNA$_i$, eIF1, eIF1A, and 40S head in py48S-open and py48S-closed.

(C) Similar position for the ZBD of eIF2β in both complexes with respect to eIF2γ. The eIF2β, eIF2γ and tRNA$_i$ of py48S-open are shown in color while those of py48S-closed are in gray. Ser264 is shown as sticks near conserved cysteines.

(D) Cartoon and surface representations of the superimposition of TC coordinates in py48S-open (color) and closed (gray) complexes based on tRNA$_i$ as reference. It shows the internal conformational change within TC during transition from the open to the closed conformation. While D2 and the helix connecting the D1 and the D2 domains of eIF2α experience an internal rearrangement, eIF2α-D3, eIF2γ and eIF2β rotate together around the acceptor arm of the tRNA.

See also Figures S6 and S7 and Movie S5

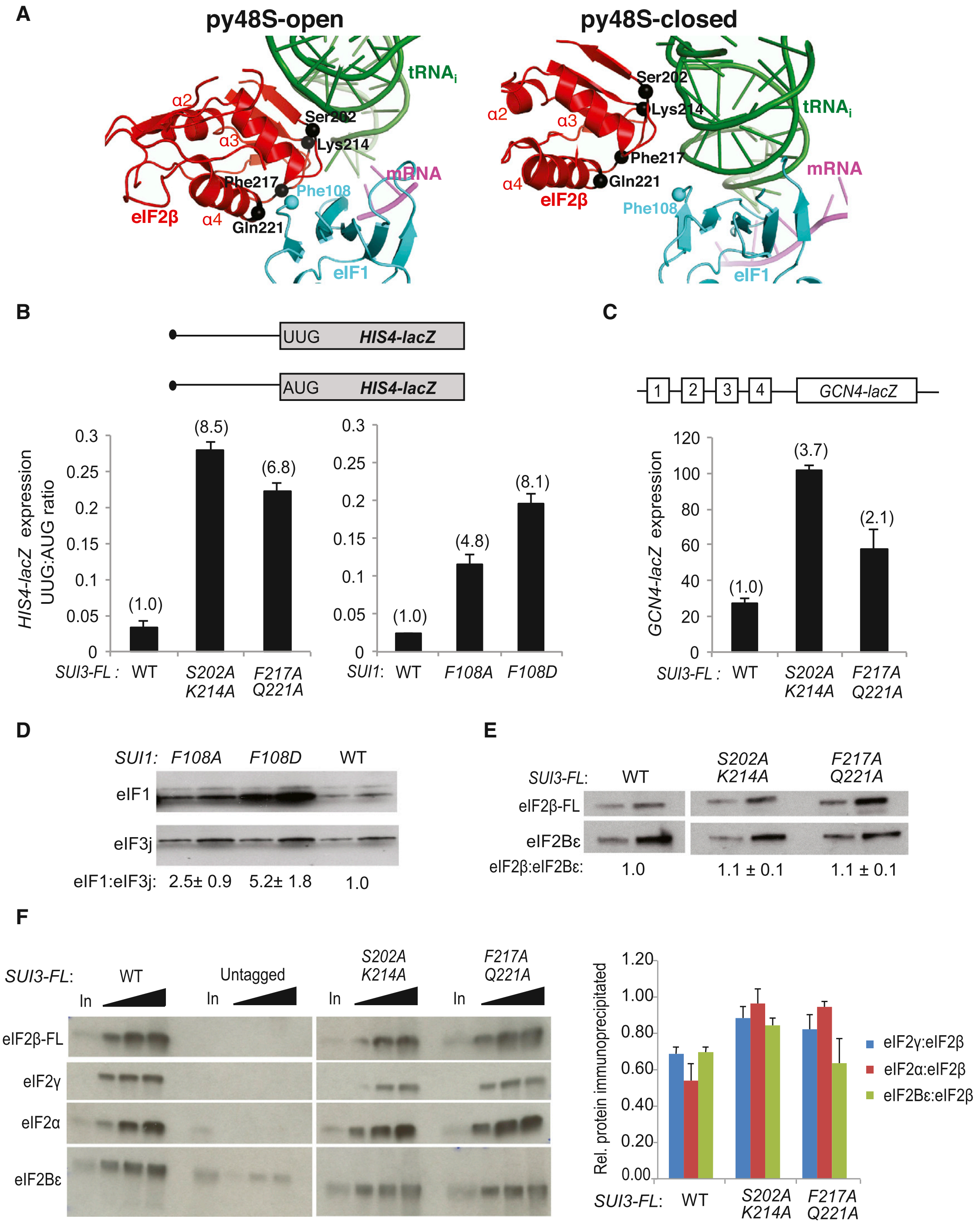

Figure 6. Genetic Evidence that eIF2β Interactions with the tRNA$_i$ ASL and eIF1 Preferentially Stabilize py48S-Open to Impede Initiation at Near-Cognate UUG Codons In Vivo

(A) Positions of eIF1, eIF2β, and tRNA$_i$ in the py48S-open and py48S-closed, with residues substituted in genetic studies shown as spheres.

(B) Expression of *HIS4-lacZ* reporters with AUG or UUG start codons in strains of the indicated *SUI3* or *SUI1* genotypes, expressed as mean (± SEM) ratios of UUG- to AUG-reporter expression with fold-changes relative to WT in parentheses.

(C) Expression of the *GCN4-lacZ* reporter expressed as mean (± SEM) units of β-galactosidase.

(D and E) Western analysis of eIF1 (D) or eIF2β (E) proteins in whole-cell extracts (WCEs), with eIF3j or eIF2Bε analyzed as loading controls, reported as mean (± SEM) eIF1:eIF3j ratios or eIF2β/eIF2Bε ratios, normalized to the WT ratios, determined from biological replicates. Lanes have been cropped from the same gels.

(legend continued on next page)

presence of additional factors here results in an overall stabilization and better local resolution for eIF1, eIF2α, eIF2γ, and $tRNA_i$ in py48S-closed (Table S1).

We observe eIF2β in py48S-open complex, where it connects eIF1 and eIF1A on the body with $tRNA_i$ on the 40S head. These bridging interactions should stabilize both TC and eIF1 binding in the scanning PIC prior to achieving a perfect AUG:anticodon duplex in the P site. Being unique to py48S-open, the eIF2β contacts with eIF1 and $tRNA_i$ should specifically stabilize the scanning complex. Consistent with this prediction, substitutions at both interfaces decreased the probability of continued scanning at near-cognate UUG start codons in yeast cells—presumably enabling rearrangement to the closed complex without a perfect start codon:anticodon duplex in the P site—thus establishing that eIF2β/eIF1 and eIF2β/$tRNA_i$ contacts in py48S-open promote initiation accuracy in vivo. The network of eIF2β interactions with eIF1/eIF1A/$tRNA_i$ should also impede mRNA insertion into the mRNA channel at the P site: eIF2β is likely repositioned to allow mRNA recruitment. Modeling either the conformation of eIF2β in py48S-closed, where it no longer contacts eIF1 and eIF1A (Figure S7A), or the distinct conformations observed in archaeal βγ complexes (Stolboushkina et al., 2008; Yatime et al., 2007) (Figure S6D), into py48S-open reveals unfettered access to the mRNA channel, supporting the notion that transient repositioning of eIF2β would allow mRNA recruitment and that eIF2β serves as a barrier to mRNA release during scanning.

These structures show how eIF3 can interact with TC and eIF1 close to the P site at the inter-subunit interface even while the majority of its contacts map to the remaining solvent-exposed surfaces of the 40S subunit. Based on our modeling, eIF3 appears to connect the entry and exit channels on the solvent face of the 40S subunit to the center of action at the P site. None of the core subunits of eIF3 has previously been observed at the subunit interface, except for eIF3j (Erzberger et al., 2014; Aylett et al., 2015), which was excluded from our study. We note however that our complexes contain mRNA, whereas all previous PICs with eIF3 lacked mRNA (Hashem et al., 2013; Erzberger et al., 2014; Aylett et al., 2015). It is likely that the position of eIF3i observed earlier (Erzberger et al., 2014; Aylett et al., 2015) may represent its position prior to mRNA binding and that the presence of mRNA in our complexes may have led to the previously unobserved conformation of eIF3 at the inter-subunit interface. Interestingly, the positioning of the eIF3b-CTD/eIF3i/eIF3g-NTD module observed here, in proximity to eIF2γ, might hinder the insertion of mRNA into the mRNA-binding channel of the ribosome, making it likely that this conformation exists only after mRNA loading and suggesting that it might lock mRNA into the scanning complex. We propose that eIF3 undergoes a substantial conformational change upon mRNA binding, relocating both the eIF3b-CTD/eIF3i/eIF3g-NTD module and portions of eIF3a and eIF3c to enable their interactions with eIF2 and eIF1 in the decoding center and thereby facilitate key steps in scanning and start codon recognition. This rearrangement may signal the presence of mRNA within the PIC to other eIFs, notably eIF2.

Integrating the complete array of structures described in this report allows us to propose a detailed scheme for assembly of the 43S PIC, mRNA recruitment to this complex, and subsequent steps of scanning and start codon recognition in the 48S PIC. In the empty 40S subunit, the position of the head with respect to the body ensures that the latch is closed (Passmore et al., 2007). Binding of eIF1 and eIF1A to assemble the 40S•eIF1•eIF1A complex (Figure S4A) leads to an 8° rotation of the head compared to the empty 40S subunit in 80S ribosomes (Ben-Shem et al., 2011) that likely facilitates binding of TC in the P_{OUT} state to form the 43S PIC (Figures S4B and S7B). Notably, the latch remains closed in both the 40S•eIF1•eIF1A and py43S complexes observed here. The 40S head is further rotated 5°–6° in the structures of py43S (without eIF3) or pm43S (without eIF1/eIF1A) (Hashem et al., 2013) relative to 40S•eIF1•eIF1A (Figure S7B), which may facilitate mRNA recruitment.

The py48S-open and py48S-closed structures (Figures 1A and 1B) illuminate a series of rearrangements that enable the PIC to first bind and scan the mRNA and then halt upon recognition of the start codon. In py48S-open, the presence of eIF1, eIF1A, TC, and eIF3 provokes an upward movement of the head away from the body, opening the latch and widening the mRNA channel between the body and head, and opening the P site, which leads to diminished contacts with $tRNA_i$ relative to the closed state (Figure S7B). We propose that this open conformation enables lateral insertion of the 5′ end of the mRNA—facilitated by the eIF4F complex bound at the cap—onto the 40S subunit (not shown). Once loaded onto the mRNA, py48S-open would be poised for scanning: the mRNA is held loosely in the channel; $tRNA_i$ is not fully engaged with the P site; the eIF1A-NTT is disordered. eIF2β interacts with $tRNA_i$, eIF1, and eIF1A to both stabilize TC binding and help hold the mRNA in the channel to promote processive scanning. The relocation of the eIF3b-CTD/eIF3i/eIF3g-NTD module near h44 on the intersubunit face, where it interacts with eIF2γ, would promote the same ends. As the open complex scans the mRNA, eIF5-mediated GTP hydrolysis by eIF2 occurs, but P_i release is blocked by the presence of eIF1, itself stabilized in the complex by its interaction with the NTD of eIF3c.

In py48S-closed, recognition of the start codon results in downward movement of the head, driven by a change in the pitch of h28 and changes in the orientation of eIF2β, closing the latch and fixing the mRNA in the channel to arrest scanning. Head closure also brings P-site elements in the 40S body into contact with the ASL, locking Met-$tRNA_i$ into the P site. Both this constriction of the entry channel and the enclosure of the P site around $tRNA_i$ (Figure S7B) are supported by recent

(F) WCEs were immunoprecipitated with FLAG affinity resin and immune complexes subjected to western analysis to detect Flag-eIF2β and co-immunoprecipitated eIF2γ, eIF2α, and eIF2Bε, resolving 1×, 2×, or 3× amounts in successive lanes. In, 20% input WCEs. Western signals were quantified to yield mean (± SEM) recoveries of eIF2γ, eIF2α, or eIF2Bε normalized to Flag-eIF2β. Lanes have been cropped from the same gels. Error bars represent the SEM from three biological replicates.

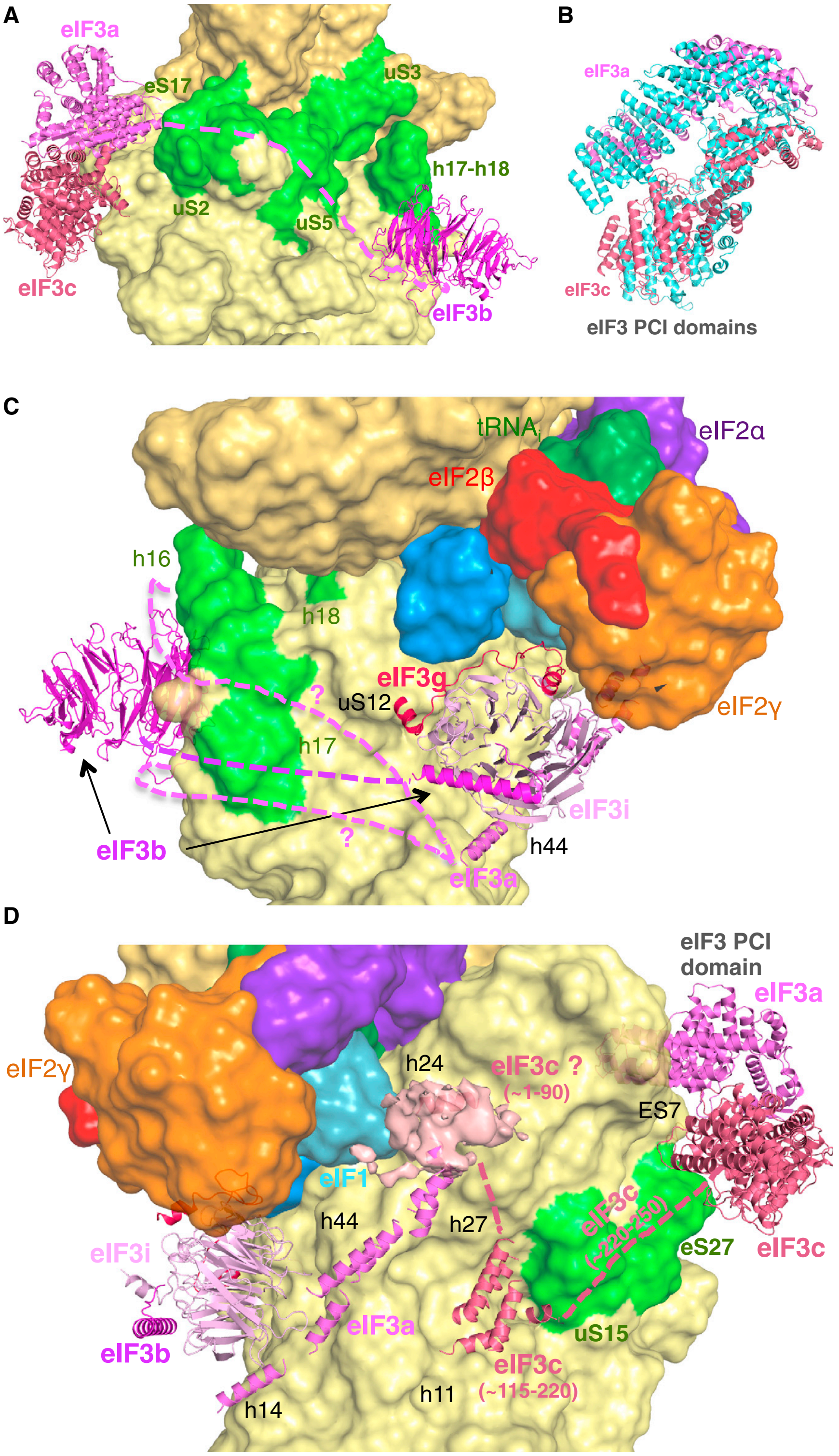

Figure 7. Structural Arrangement of eIF3 Components in 48S PICs

(A) Locations of the eIF3a/eIF3c PCI domains and β-propeller of eIF3b at different positions on the solvent-exposed surface of the 40S, highlighting rRNA helices and ribosomal proteins (green) predicted to bind to eIF3a. The proposed path of the unassigned central portion of the eIF3a-CTD connecting the PCI domain to the subunit interface is shown as a dashed purple line.

(B) Lateral displacement of eIF3a/eIF3c PCI domains in py48S-closed versus their positions in yeast 40S•eIF1•eIF1A•eIF3 (PDB: 4UER).

(C) Trimeric eIF3b-CTD/eIF3i/eIF3g-NTD subcomplex is shown near h44 and interacting with eIF2γ and the 40S interface surface. The β-propeller of eIF3b is also shown. Two alternative proposed paths of the eIF3a-CTD connecting the PCI domain to the bundle of helices below the eIF3i β-propeller are shown as dashed purple lines.

(D) A cluster of helices tentatively assigned to eIF3c is located near h11 and uS15 (green). A globular density with a single modeled helix is tentatively assigned to the eIF3c-NTD in proximity to eIF1 and h24. The proposed path of a linker connecting the cluster of helices to the eIF3c PCI domain is shown as a dashed magenta line. Long helices tentatively assigned to eIF3a bridge the eIF3i β-propeller and h44 with the putative eIF3c-NTD and h24.

hydroxyl radical probing of yeast 48S complexes reconstituted with AUG versus AUC start codons (Zhang et al., 2015). These and other rearrangements stabilize the P_{IN} state of the closed complex (Figure S7B): interactions between eIF2β and the $tRNA_i$ are remodeled; eIF2β exchanges its contacts with eIF1 and eIF1A for those with the 40S head; the eIF1A-NTT interacts with the AUG:anticodon duplex. Other rearrangements deform eIF1; different portions of $tRNA_i$ are brought into contact with eIF1, adjusting its position and promoting its eviction from the 40S subunit, which provokes P_i release and commits the complex to subunit joining. P_i release may also trigger detachment of eIF3b-CTD/eIF3i/eIF3g-NTD from the subunit interface, paving the way for release of eIF2, binding of eIF5B, and joining of the 60S subunit. Conformational changes within the TC in py48S-closed also bring the eIF2α-D1 loop in contact with the key −3 nt upstream of the start codon to regulate AUG selection (Hussain et al., 2014).

In summary, the py48S-open and py48S-closed structures described here address long-standing questions about various aspects of initiation. Comparison of these structures reveal how the PIC in the open state may facilitate both loading of the mRNA and subsequent scanning, all while holding both TC and mRNA in place for processive inspection of codons within the P site. Upon recognition of the

start codon, the PIC closes, both locking mRNA and $tRNA_i$ within the P site and preparing eIF1 for its departure from the complex. Our structures also reveal how eIF3, bound at the 40S solvent face, may encircle the PIC, linking the mRNA entry and exit channels with the locus of action near the P site.

EXPERIMENTAL PROCEDURES

Electron Microscopy

Data were collected on an FEI Titan Krios microscope operated at 300 kV under low-dose conditions (27 $e^{-}/Å^{2}$) using a defocus range of 1.8–3.2 μm. Images were recorded on a Falcon II detector at a calibrated magnification of 104,478 (yielding a pixel size of 1.34 Å). An in-house system was used to intercept the videos from the detector at a speed of 16 frames/s exposures. Micrographs that showed noticeable signs of astigmatism or drift were discarded.

Analysis and Structure Determination

Particles were picked using RELION (Scheres, 2012). Contrast transfer function parameters for the micrographs were estimated using CTFFIND3 (Mindell and Grigorieff, 2003). 2D class averaging, 3D classification, and refinements were done using RELION (Scheres, 2012).

Statistical movie processing was done (Bai et al., 2013) to improve resolution of all reconstructions. Resolutions reported are based on the gold-standard FSC = 0.143 criterion (Scheres and Chen, 2012). Local resolution was estimated using RESMAP (Kucukelbir et al., 2014). All maps were further processed for the modulation transfer function of the detector and sharpened by applying negative B factors (-20 $Å^2$ for py48S-open and -119 $Å^2$ py48S-closed; estimated as in Rosenthal and Henderson, 2003).

Model Building and Refinement

The atomic model of py48S (PDB: 3J81) was placed into density by rigid-body fitting using Chimera (Pettersen et al., 2004). Further model building was done in Coot (Emsley et al., 2010). For py48S-open, the body and head of the 40S were independently placed. For eIF2β, models from its archaeal counterpart were employed (PDB: 3CW2, 2D74). Wild-type $tRNA_i$ was used from PDB: 1YFG for initial rigid-body fitting into its corresponding density in the py48S-open complex. Model building and refinement were carried out using Coot and Refmac (Brown et al., 2015) (see Supplemental Experimental Procedures). All figures were generated using PyMOL (DeLano, 2006) or Chimera.

ACCESSION NUMBERS

Maps have been deposited in the EMDB with accession codes EMD: EMD-3047, EMD-3048, EMD-3049, EMD-3050 for the 40S•eIF1•eIF1A, py48S-closed, py48S-open, and py43S, respectively. Atomic coordinates have been deposited in the PDB with accession codes PDB: 3JAM, 3JAP, 3JAQ for 40S•eIF1•eIF1A, py48S-closed, and py48S-open, respectively.

SUPPLEMENTAL INFORMATION

Supplemental Information includes seven figures, one table, five movies, and Supplemental Experimental Procedures and can be found with this article online at http://dx.doi.org/10.1016/j.molcel.2015.06.033.

AUTHOR CONTRIBUTIONS

J.L.L. and T.H. made the samples, collected and analyzed the data, determined the structures and wrote a first draft of the manuscript. L.M. and A.T. performed the genetic experiments. C.E.A. provided advice on eIF3 purification, helped characterize recombinant eIF3, and helped write the manuscript. J.R.L., A.G.H., and V.R. supervised the work and helped to write the manuscript.

ACKNOWLEDGMENTS

We are grateful to S. Chen, C.G. Savva, K.R. Vinothkumar, G. McMullan, and the staff of FEI for technical support with cryo-EM; T. Darling and J. Grimmett for help with computing; X.C. Bai and S.H.W. Scheres for advice with EM data processing; and G. Murshudov for help with refinement of the atomic coordinates. We also thank A. Kelley and I.S. Fernandez for providing reagents. J.L.L. and T.H. were, respectively, supported by postdoctoral fellowships from FEBS and EMBO. This work was funded by grants to from the UK Medical Research Council (MC_U105184332), Wellcome Trust Senior Investigator award (WT096570), the Agouron Institute and the Jeantet Foundation to V.R.; from the NIH (GM62128) formerly to J.R.L.; the Human Frontiers in Science Program (RGP-0028/2009) to A.G.H., J.R.L., and V.R.; and by the Intramural Research Program of the NIH (A.G.H., J.R.L., and C.E.A.).

Received: March 12, 2015
Revised: May 18, 2015
Accepted: June 24, 2015
Published: July 23, 2015

REFERENCES

Algire, M.A., Maag, D., and Lorsch, J.R. (2005). Pi release from eIF2, not GTP hydrolysis, is the step controlled by start-site selection during eukaryotic translation initiation. Mol. Cell *20*, 251–262.

Asano, K., Clayton, J., Shalev, A., and Hinnebusch, A.G. (2000). A multifactor complex of eukaryotic initiation factors, eIF1, eIF2, eIF3, eIF5, and initiator tRNA(Met) is an important translation initiation intermediate in vivo. Genes Dev. *14*, 2534–2546.

Aylett, C.H., Boehringer, D., Erzberger, J.P., Schaefer, T., and Ban, N. (2015). Structure of a yeast 40S-eIF1-eIF1A-eIF3-eIF3j initiation complex. Nat. Struct. Mol. Biol. *22*, 269–271.

Bai, X.C., Fernandez, I.S., McMullan, G., and Scheres, S.H. (2013). Ribosome structures to near-atomic resolution from thirty thousand cryo-EM particles. Elife *2*, e00461.

Ben-Shem, A., Garreau de Loubresse, N., Melnikov, S., Jenner, L., Yusupova, G., and Yusupov, M. (2011). The structure of the eukaryotic ribosome at 3.0 Å resolution. Science *334*, 1524–1529.

Brown, A., Long, F., Nicholls, R.A., Toots, J., Emsley, P., and Murshudov, G. (2015). Tools for macromolecular model building and refinement into electron cryo-microscopy reconstructions. Acta Crystallogr. D Biol. Crystallogr. *71*, 136–153.

Chiu, W.L., Wagner, S., Herrmannová, A., Burela, L., Zhang, F., Saini, A.K., Valásek, L., and Hinnebusch, A.G. (2010). The C-terminal region of eukaryotic translation initiation factor 3a (eIF3a) promotes mRNA recruitment, scanning, and, together with eIF3j and the eIF3b RNA recognition motif, selection of AUG start codons. Mol. Cell. Biol. *30*, 4415–4434.

Cuchalová, L., Kouba, T., Herrmannová, A., Dányi, I., Chiu, W.L., and Valásek, L. (2010). The RNA recognition motif of eukaryotic translation initiation factor 3g (eIF3g) is required for resumption of scanning of posttermination ribosomes for reinitiation on GCN4 and together with eIF3i stimulates linear scanning. Mol. Cell. Biol. *30*, 4671–4686.

DeLano, W.L. (2006). The PyMOL Molecular Graphics System. http://www.pymol.org.

Donahue, T.F., Cigan, A.M., Pabich, E.K., and Valavicius, B.C. (1988). Mutations at a Zn(II) finger motif in the yeast eIF-2 beta gene alter ribosomal start-site selection during the scanning process. Cell *54*, 621–632.

Dong, J., Nanda, J.S., Rahman, H., Pruitt, M.R., Shin, B.S., Wong, C.M., Lorsch, J.R., and Hinnebusch, A.G. (2008). Genetic identification of yeast 18S rRNA residues required for efficient recruitment of initiator tRNA(Met) and AUG selection. Genes Dev. *22*, 2242–2255.

Dong, Z., Qi, J., Peng, H., Liu, J., and Zhang, J.T. (2013). Spectrin domain of eukaryotic initiation factor 3a is the docking site for formation of the a:b:i:g subcomplex. J. Biol. Chem. *288*, 27951–27959.

Dong, J., Munoz, A., Kolitz, S.E., Saini, A.K., Chiu, W.L., Rahman, H., Lorsch, J.R., and Hinnebusch, A.G. (2014). Conserved residues in yeast initiator tRNA calibrate initiation accuracy by regulating preinitiation complex stability at the start codon. Genes Dev. *28*, 502–520.

Emsley, P., Lohkamp, B., Scott, W.G., and Cowtan, K. (2010). Features and development of Coot. Acta Crystallogr. D Biol. Crystallogr. *66*, 486–501.

Erzberger, J.P., Stengel, F., Pellarin, R., Zhang, S., Schaefer, T., Aylett, C.H., Cimermančič, P., Boehringer, D., Sali, A., Aebersold, R., and Ban, N. (2014). Molecular architecture of the 40S•eIF1•eIF3 translation initiation complex. Cell *158*, 1123–1135.

Fletcher, C.M., Pestova, T.V., Hellen, C.U., and Wagner, G. (1999). Structure and interactions of the translation initiation factor eIF1. EMBO J. *18*, 2631–2637.

Hashem, Y., des Georges, A., Dhote, V., Langlois, R., Liao, H.Y., Grassucci, R.A., Hellen, C.U., Pestova, T.V., and Frank, J. (2013). Structure of the mammalian ribosomal 43S preinitiation complex bound to the scanning factor DHX29. Cell *153*, 1108–1119.

Herrmannová, A., Daujotyte, D., Yang, J.C., Cuchalová, L., Gorrec, F., Wagner, S., Dányi, I., Lukavsky, P.J., and Valásek, L.S. (2012). Structural analysis of an eIF3 subcomplex reveals conserved interactions required for a stable and proper translation pre-initiation complex assembly. Nucleic Acids Res. *40*, 2294–2311.

Hinnebusch, A.G. (2005). Translational regulation of GCN4 and the general amino acid control of yeast. Annu. Rev. Microbiol. *59*, 407–450.

Hinnebusch, A.G. (2006). eIF3: a versatile scaffold for translation initiation complexes. Trends Biochem. Sci. *31*, 553–562.

Hinnebusch, A.G. (2014). The scanning mechanism of eukaryotic translation initiation. Annu. Rev. Biochem. *83*, 779–812.

Hinnebusch, A.G., and Lorsch, J.R. (2012). The mechanism of eukaryotic translation initiation: new insights and challenges. Cold Spring Harb. Perspect. Biol. *4*, http://dx.doi.org/10.1101/cshperspect.a011544.

Hussain, T., Llacer, J.L., Fernandez, I.S., Munoz, A., Martin-Marcos, P., Savva, C.G., Lorsch, J.R., Hinnebusch, A.G., and Ramakrishnan, V. (2014). Structural changes enable start codon recognition by the eukaryotic translation initiation complex. Cell *159*, 597–607.

Jackson, R.J., Hellen, C.U., and Pestova, T.V. (2010). The mechanism of eukaryotic translation initiation and principles of its regulation. Nat. Rev. Mol. Cell Biol. *11*, 113–127.

Karásková, M., Gunišová, S., Herrmannová, A., Wagner, S., Munzarová, V., and Valášek, L. (2012). Functional characterization of the role of the N-terminal domain of the c/Nip1 subunit of eukaryotic initiation factor 3 (eIF3) in AUG recognition. J. Biol. Chem. *287*, 28420–28434.

Kucukelbir, A., Sigworth, F.J., and Tagare, H.D. (2014). Quantifying the local resolution of cryo-EM density maps. Nat. Methods *11*, 63–65.

Lomakin, I.B., and Steitz, T.A. (2013). The initiation of mammalian protein synthesis and mRNA scanning mechanism. Nature *500*, 307–311.

Maag, D., Algire, M.A., and Lorsch, J.R. (2006). Communication between eukaryotic translation initiation factors 5 and 1A within the ribosomal pre-initiation complex plays a role in start site selection. J. Mol. Biol. *356*, 724–737.

Martin-Marcos, P., Cheung, Y.N., and Hinnebusch, A.G. (2011). Functional elements in initiation factors 1, 1A, and 2β discriminate against poor AUG context and non-AUG start codons. Mol. Cell. Biol. *31*, 4814–4831.

Martin-Marcos, P., Nanda, J.S., Luna, R.E., Zhang, F., Saini, A.K., Cherkasova, V.A., Wagner, G., Lorsch, J.R., and Hinnebusch, A.G. (2014). Enhanced eIF1 binding to the 40S ribosome impedes conformational rearrangements of the preinitiation complex and elevates initiation accuracy. RNA *20*, 150–167.

Mindell, J.A., and Grigorieff, N. (2003). Accurate determination of local defocus and specimen tilt in electron microscopy. J. Struct. Biol. *142*, 334–347.

Nanda, J.S., Saini, A.K., Muñoz, A.M., Hinnebusch, A.G., and Lorsch, J.R. (2013). Coordinated movements of eukaryotic translation initiation factors eIF1, eIF1A, and eIF5 trigger phosphate release from eIF2 in response to start codon recognition by the ribosomal preinitiation complex. J. Biol. Chem. *288*, 5316–5329.

Passmore, L.A., Schmeing, T.M., Maag, D., Applefield, D.J., Acker, M.G., Algire, M.A., Lorsch, J.R., and Ramakrishnan, V. (2007). The eukaryotic translation initiation factors eIF1 and eIF1A induce an open conformation of the 40S ribosome. Mol. Cell *26*, 41–50.

Pestova, T.V., and Kolupaeva, V.G. (2002). The roles of individual eukaryotic translation initiation factors in ribosomal scanning and initiation codon selection. Genes Dev. *16*, 2906–2922.

Pestova, T.V., Borukhov, S.I., and Hellen, C.U. (1998). Eukaryotic ribosomes require initiation factors 1 and 1A to locate initiation codons. Nature *394*, 854–859.

Pettersen, E.F., Goddard, T.D., Huang, C.C., Couch, G.S., Greenblatt, D.M., Meng, E.C., and Ferrin, T.E. (2004). UCSF Chimera—a visualization system for exploratory research and analysis. J. Comput. Chem. *25*, 1605–1612.

Pisarev, A.V., Kolupaeva, V.G., Yusupov, M.M., Hellen, C.U., and Pestova, T.V. (2008). Ribosomal position and contacts of mRNA in eukaryotic translation initiation complexes. EMBO J. *27*, 1609–1621.

Rabl, J., Leibundgut, M., Ataide, S.F., Haag, A., and Ban, N. (2011). Crystal structure of the eukaryotic 40S ribosomal subunit in complex with initiation factor 1. Science *331*, 730–736.

Reibarkh, M., Yamamoto, Y., Singh, C.R., del Rio, F., Fahmy, A., Lee, B., Luna, R.E., Ii, M., Wagner, G., and Asano, K. (2008). Eukaryotic initiation factor (eIF) 1 carries two distinct eIF5-binding faces important for multifactor assembly and AUG selection. J. Biol. Chem. *283*, 1094–1103.

Rosenthal, P.B., and Henderson, R. (2003). Optimal determination of particle orientation, absolute hand, and contrast loss in single-particle electron cryomicroscopy. J. Mol. Biol. *333*, 721–745.

Scheres, S.H. (2012). RELION: implementation of a Bayesian approach to cryo-EM structure determination. J. Struct. Biol. *180*, 519–530.

Scheres, S.H., and Chen, S. (2012). Prevention of overfitting in cryo-EM structure determination. Nat. Methods *9*, 853–854.

Siridechadilok, B., Fraser, C.S., Hall, R.J., Doudna, J.A., and Nogales, E. (2005). Structural roles for human translation factor eIF3 in initiation of protein synthesis. Science *310*, 1513–1515.

Sokabe, M., Yao, M., Sakai, N., Toya, S., and Tanaka, I. (2006). Structure of archaeal translational initiation factor 2 betagamma-GDP reveals significant conformational change of the beta-subunit and switch 1 region. Proc. Natl. Acad. Sci. USA *103*, 13016–13021.

Stolboushkina, E., Nikonov, S., Nikulin, A., Bläsi, U., Manstein, D.J., Fedorov, R., Garber, M., and Nikonov, O. (2008). Crystal structure of the intact archaeal translation initiation factor 2 demonstrates very high conformational flexibility in the alpha- and beta-subunits. J. Mol. Biol. *382*, 680–691.

Valásek, L.S. (2012). 'Ribozoomin'—translation initiation from the perspective of the ribosome-bound eukaryotic initiation factors (eIFs). Curr. Protein Pept. Sci. *13*, 305–330.

Valásek, L., Nielsen, K.H., and Hinnebusch, A.G. (2002). Direct eIF2-eIF3 contact in the multifactor complex is important for translation initiation in vivo. EMBO J. *21*, 5886–5898.

Valásek, L., Mathew, A.A., Shin, B.S., Nielsen, K.H., Szamecz, B., and Hinnebusch, A.G. (2003). The yeast eIF3 subunits TIF32/a, NIP1/c, and eIF5 make critical connections with the 40S ribosome in vivo. Genes Dev. *17*, 786–799.

Valásek, L., Nielsen, K.H., Zhang, F., Fekete, C.A., and Hinnebusch, A.G. (2004). Interactions of eukaryotic translation initiation factor 3 (eIF3) subunit NIP1/c with eIF1 and eIF5 promote preinitiation complex assembly and regulate start codon selection. Mol. Cell. Biol. *24*, 9437–9455.

Voigts-Hoffmann, F., Klinge, S., and Ban, N. (2012). Structural insights into eukaryotic ribosomes and the initiation of translation. Curr. Opin. Struct. Biol. *22*, 768–777.

Weisser, M., Voigts-Hoffmann, F., Rabl, J., Leibundgut, M., and Ban, N. (2013). The crystal structure of the eukaryotic 40S ribosomal subunit in complex with eIF1 and eIF1A. Nat. Struct. Mol. Biol. *20*, 1015–1017.

Yatime, L., Mechulam, Y., Blanquet, S., and Schmitt, E. (2007). Structure of an archaeal heterotrimeric initiation factor 2 reveals a nucleotide state between the GTP and the GDP states. Proc. Natl. Acad. Sci. USA *104*, 18445–18450.

Yu, Y., Marintchev, A., Kolupaeva, V.G., Unbehaun, A., Veryasova, T., Lai, S.C., Hong, P., Wagner, G., Hellen, C.U., and Pestova, T.V. (2009). Position of eukaryotic translation initiation factor eIF1A on the 40S ribosomal subunit mapped by directed hydroxyl radical probing. Nucleic Acids Res. *37*, 5167–5182.

Zhang, F., Saini, A.K., Shin, B.S., Nanda, J., and Hinnebusch, A.G. (2015). Conformational changes in the P site and mRNA entry channel evoked by AUG recognition in yeast translation preinitiation complexes. Nucleic Acids Res. *43*, 2293–2312.

Molecular Cell, Volume *59*

Supplemental Information

Conformational Differences

between Open and Closed States

# of the Eukaryotic Translation Initiation Complex

Jose L. Llácer, Tanweer Hussain, Laura Marler, Colin Echeverría Aitken, Anil Thakur, Jon R. Lorsch, Alan G. Hinnebusch, and V. Ramakrishnan

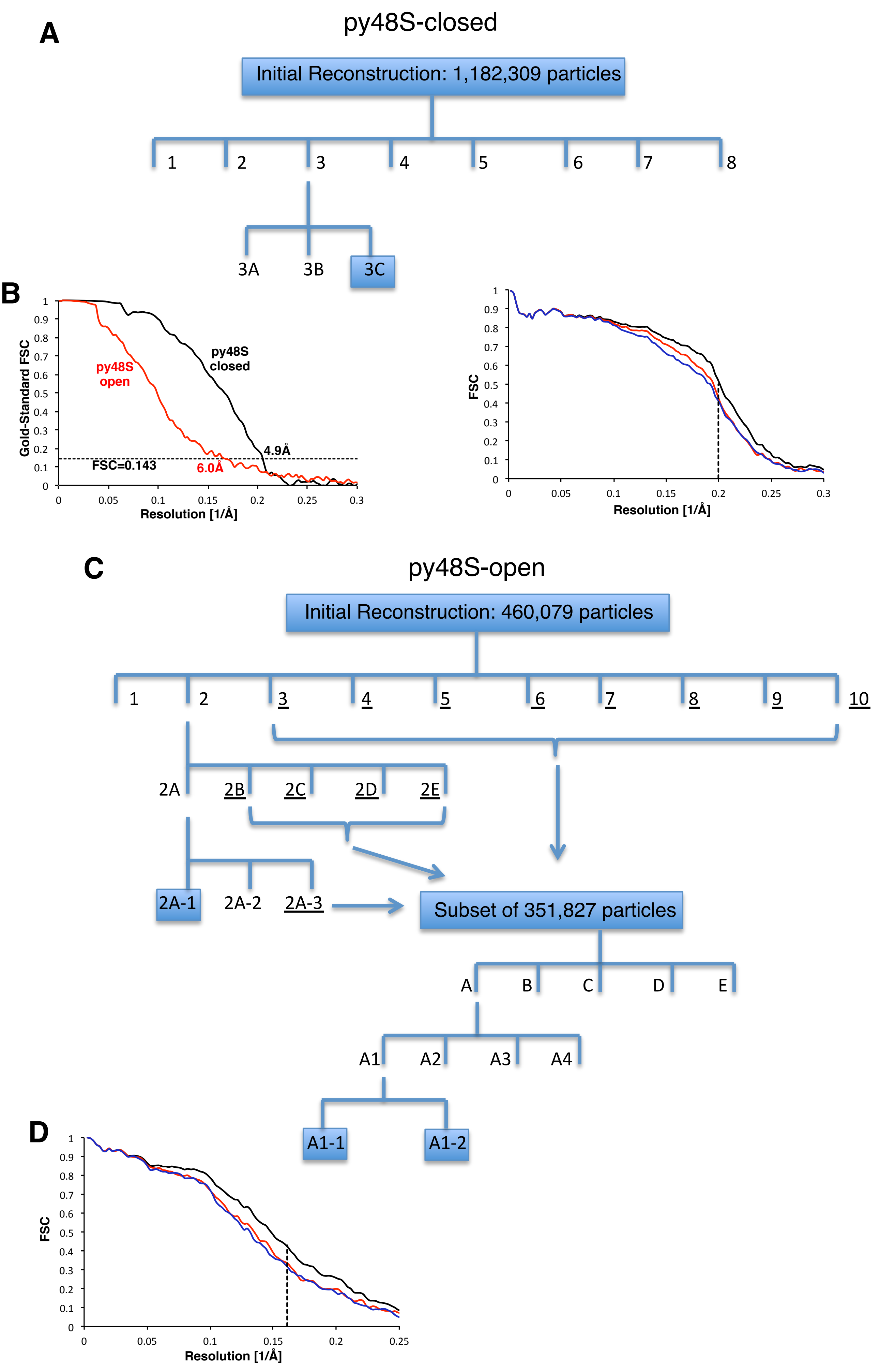
A
py48S-closed
Initial Reconstruction: 1,182,309 particles
1
2
3
4
5
6
7
8
3A
3B
3C
B
Gold-Standard FSC
py48S open
py48S closed
FSC=0.143
6.0Å
4.9Å
Resolution [1/Å]
FSC
Resolution [1/Å]
C
py48S-open
Initial Reconstruction: 460,079 particles
1
2
3
4
5
6
7
8
9
10
2A
2B
2C
2D
2E
2A-1
2A-2
2A-3
Subset of 351,827 particles
A
B
C
D
E
A1
A2
A3
A4
A1-1
A1-2
D
FSC
Resolution [1/Å]

Figure S1. Maximum-likelihood 3D classification scheme and data processing quality of density maps, related to Figure 1

Maximum-likelihood 3D classification schemes (See Supplemental Experimental Procedures):

(A) py48S-closed complex: Class 3C (21,401 particles; 4.9 Å) corresponding to py48S-closed complex is highlighted in a box.

(B) At the left, Gold-standard Fourier Shell Correlation (FSC) curves for the py48S-closed (black) and py48S-open (red) complexes. At the right, analysis of overfitting by cross-validation of the py48S-closed model. FSC_{work} curves (red) corresponding to the refined model versus the half-map it was refined against, and FSC_{test} curves (blue), i.e. those calculated between the refined atomic model and the other half-map. The black curve shows the FSC curve between a reconstruction from all particles and the model refined against the map. The dashed line represents the highest resolution (5.0Å) used in these refinements.

(C) py48S-open complex: Class 2A-1 (86,055 particles; 3.5 Å) corresponding to 40S•eIF1•eIF1A complex, Class A1-1 (4,547 particles; 6.0 Å) corresponding to py48S-open complex and Class A1-2 (1,580 particles; 14.9 Å) corresponding to py43S are highlighted in a box.

(D) Analysis of overfitting by cross-validation of the py48S-open model. FSC_{work} curves (red) corresponding to the refined model versus the half-map it was refined against, and FSC_{test} curves (blue), i.e. those calculated between the refined atomic model and the other half-map. The black curve shows the FSC curve between a reconstruction from all particles and the model refined against the map. The dashed line represents the highest resolution (6.2Å) used in these refinements.

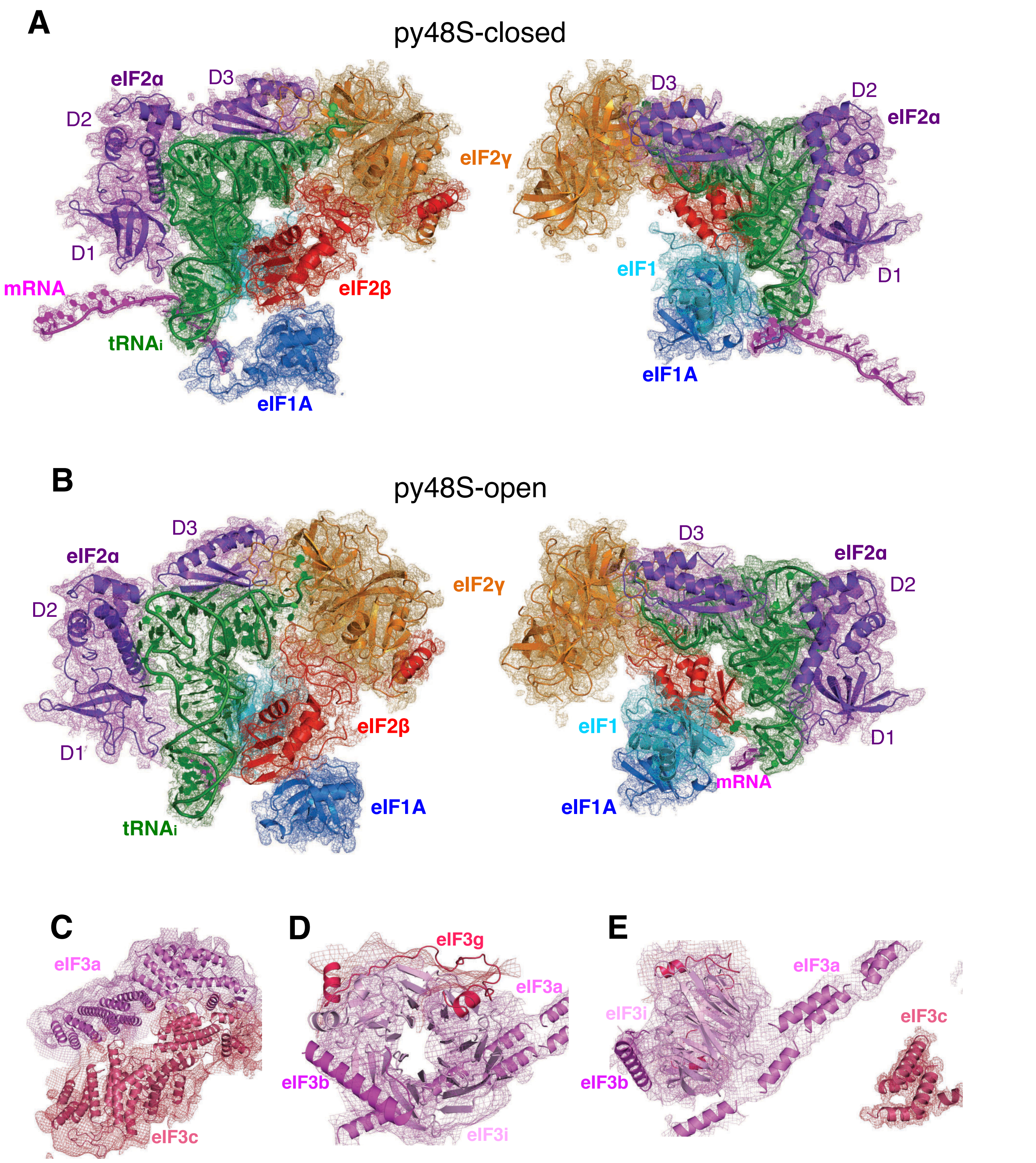

Figure S2. Fitting of ligands in density maps, related to Figure 1

(A) Fitting of eIF1, eIF1A, eIF2, tRNAi and mRNA in py48S-closed map at 4.9 Å shown in two orientations.

(B) Fitting of eIF1, eIF1A, eIF2, tRNAi and mRNA in py48S-open map at 6.0 Å shown in two orientations.

(C) eIF3a/eIF3c PCI heterodimer in py48S-closed map.

(D) eIF3b-CTD/eIF3i/eIF3g-NTD in py48S-closed map.

(E) eIF3b-CTD/eIF3i/eIF3g-NTD trimer, cluster of eIF3c helices and bundle of eIF3a long helices in py48S-closed map.

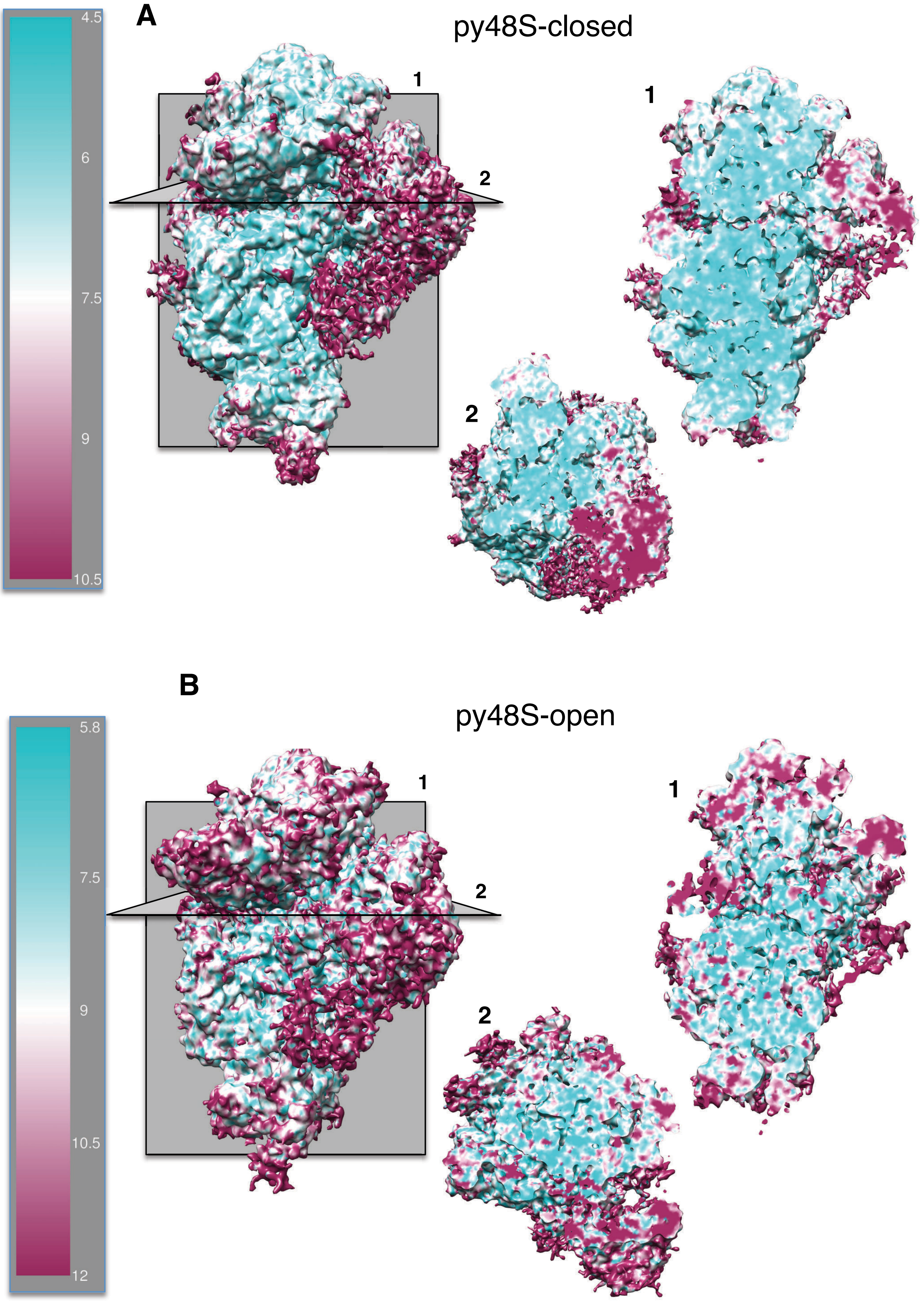

A
py48S-closed
4.5
6
7.5
9
10.5
1
2
1
2
B
py48S-open
5.8
7.5
9
10.5
12
1
2
1
2

Figure S3. Local resolution features, related to Figure 1

(A) Surface (left) and cross-sections along the mRNA channel in two different planes of an 4.9 Å map, colored according to local resolution (See Experimental Procedures) of py48S-closed complex.

(B) Surface (left) and cross-sections along the mRNA channel in two different planes of an 6.0 Å map, colored according to local resolution (See Experimental Procedures) of py48S-open complex.

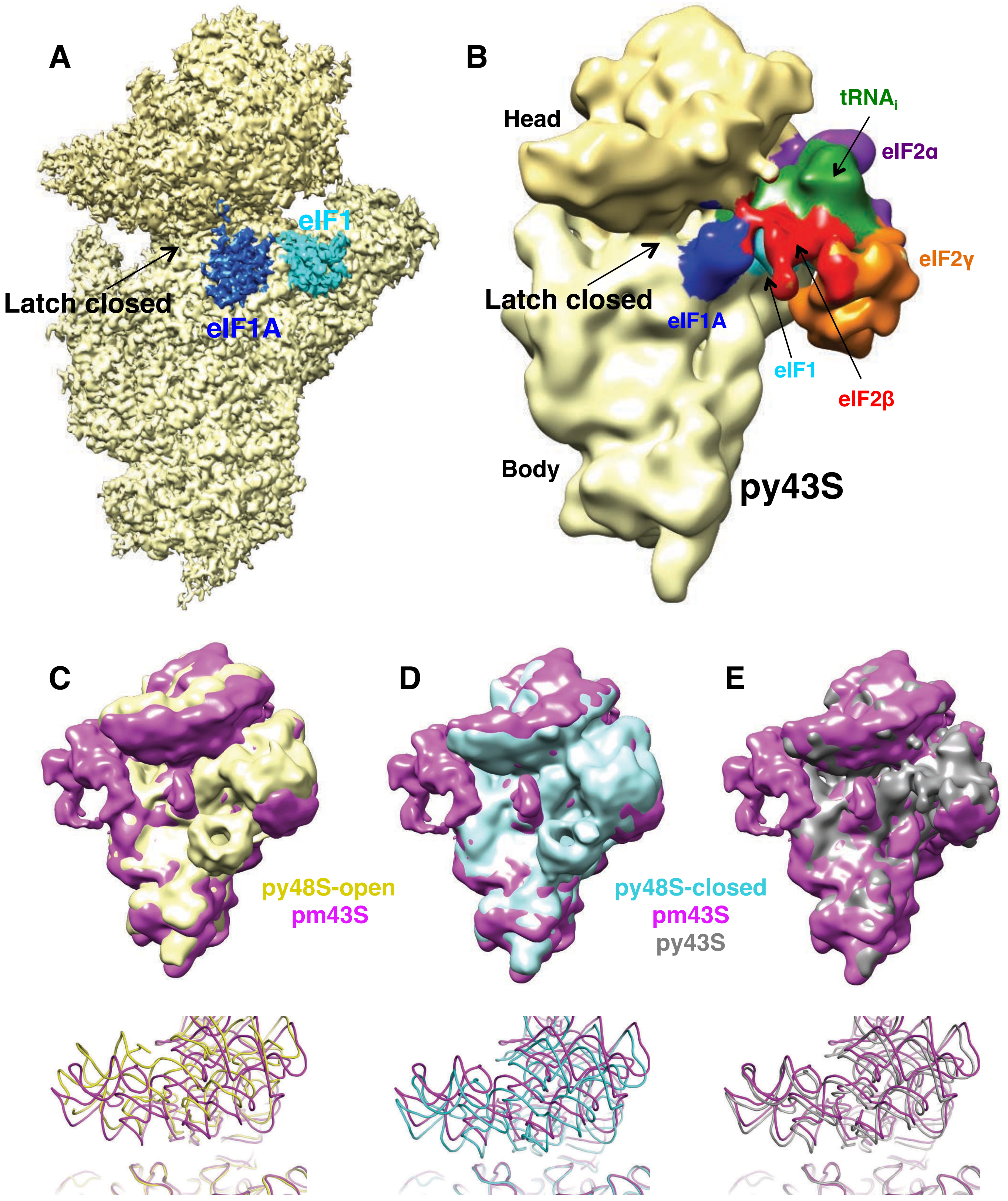
A
elF1
Latch closed
elF1A
B
Head
tRNA$_i$
elF2α
elF2γ
Latch closed
elF1A
elF1
elF2β
Body
py43S
C
D
E
py48S-open
pm43S
py48S-closed
pm43S
py43S

Figure S4. Cryo-EM maps of py43S and 40S•eIF1•eIF1A complexes, related to Figures 1 and 2
(A) Cryo-EM map of 40S•eIF1•eIF1A PIC at 3.5 Å. Density for eIF1 (cyan) and eIF1A (blue) can be clearly seen. The structure of the 40S•eIF1•eIF1A complex is similar to the PIC-2 complex reported earlier (Hussain et al., 2014) with an r.m.s.d. of 0.99 Å for 35,235 atoms of 18S rRNA.
(B) Cryo-EM map of py43S PIC at 15.0 Å. Density for eIF1, eIF1A and TC is observed.
(C) Superimposition of py48S-open (yellow) and pm43S (magenta) (Hashem et al., 2013) maps. *Below:* Cartoon representation of rRNAs of the two structures. The head is clearly moved up in py48S-open. The py48S-open map is low-pass filtered to 12 Å.
(D) Superimposition of py48S-closed (cyan) and pm43S (magenta) (Hashem et al., 2013) maps. *Below:* Cartoon representation of rRNAs of the two structures. The head is in a similar but not identical position in the two complexes. The py48S-closed map is low-pass filtered to 12 Å.
(E) Superimposition of py43S (grey) and pm43S (magenta) (Hashem et al., 2013) maps. *Below:* Cartoon representation of rRNAs of the two structures. The head is almost identical in both complexes.

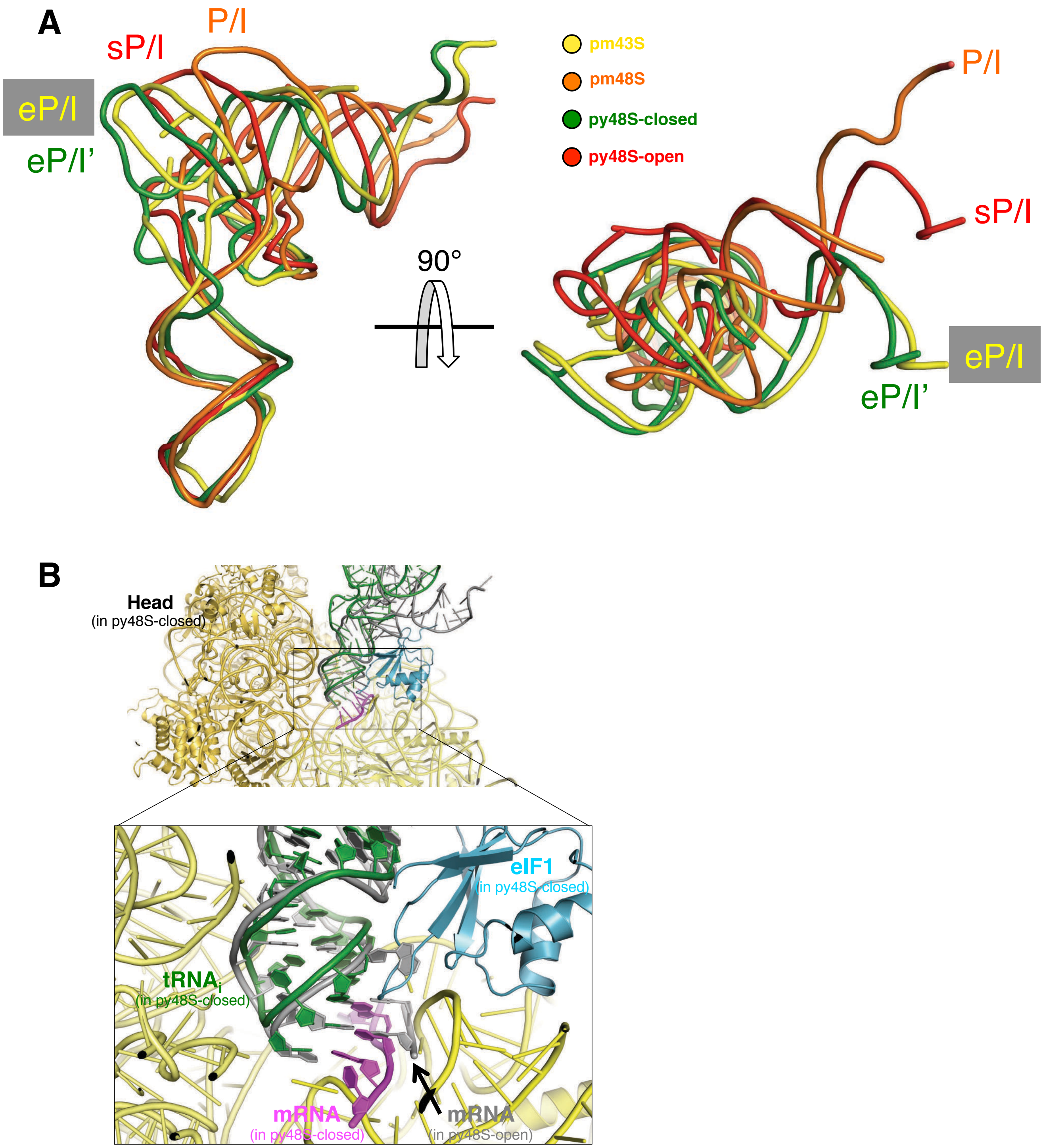

Figure S5. Distinct tRNA$_i$ conformations and the mRNA path, related to Figure 3

(A) Two different views of the superimposition of tRNA$_i$ from different complexes aligned to the head of 40S. The conformation of tRNA$_i$ from py48S-closed (eP/I', green) is similar to that described for pm43S complex [eP/I**,** yellow; (Hashem et al., 2013)], but different from the P/I conformation (from pm48S PIC, in orange, from 4KZZ). The tRNA$_i$ from py48S-open (red) complex is in an orientation that appears closer to P/I than the eP/I. We have termed this orientation sP/I (scanning P/I).

(B) Superimposition of py48S- open and closed complexes aligned to the 40S head shows the relative position of tRNA$_i$ in the P site. The body and head of py48S-closed complex is shown in yellow with its tRNA$_i$ in green. The mRNA (magenta) and β-hairpin 1 of eIF1 (cyan) of py48S-closed complex interact at the P site. The mRNA and tRNA$_i$ of py48S-open complex is shown in grey. The mRNA in the py48S-open complex would clash with the body of 40S in the py48S-closed complex.

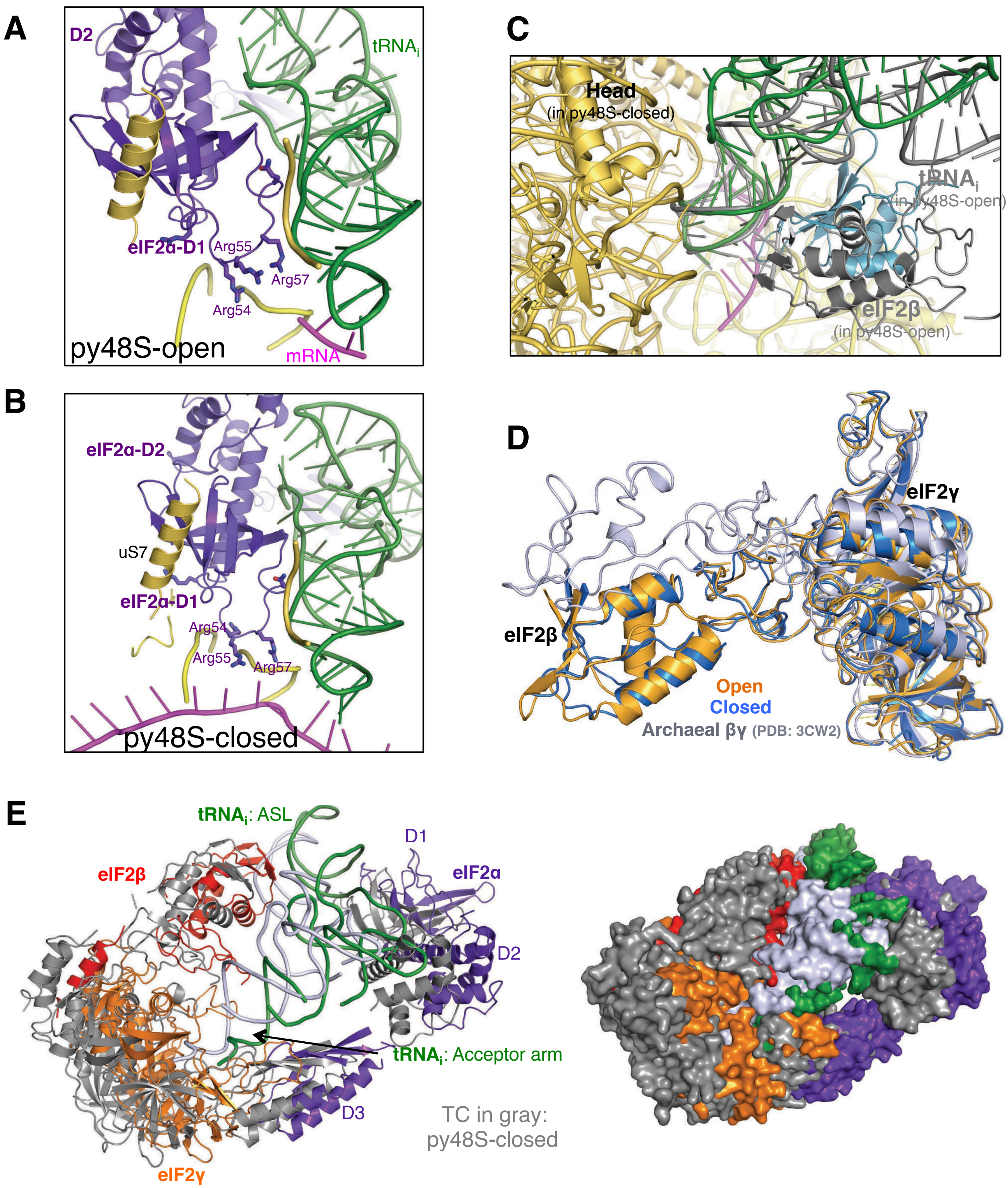
A
D2
tRNAi
eIF2α-D1
Arg55
Arg57
Arg54
py48S-open
mRNA
B
eIF2α-D2
uS7
eIF2α-D1
Arg54
Arg55
Arg57
py48S-closed
C
Head
(in py48S-closed)
tRNAi
(in py48S-open)
eIF2β
(in py48S-open)
D
eIF2γ
eIF2β
Open
Closed
Archaeal βγ (PDB: 3CW2)
E
tRNAi: ASL
D1
eIF2β
eIF2α
D2
tRNAi: Acceptor arm
D3
TC in gray:
py48S-closed
eIF2γ

Figure S6. Relative orientations of eIF2 in py48S- open and closed PICs, related to Figure 5

(A) eIF2α-D1 and ASL of $tRNA_i$ in py48S-open complex. The neighbouring rRNA residues are shown in yellow lines and a helix of uS7 is also shown in yellow. Conserved arginines are shown. Arg55 and Arg57 project away from the modeled mRNA (grey; from py48S-closed complex), and Arg54, which in the closed complex interacts with the body of the 40S, comes closer to the mRNA.

(B) eIF2α-D1 and the ASL of $tRNA_i$ in the py48S-closed complex. The neighbouring rRNA residues are shown in yellow lines and a helix of uS7 is also shown in yellow. Conserved arginines are shown. Arg55 and Arg57 interact with the mRNA (magenta).

(C) Superimposition of py48S- closed and open complexes shows that eIF2β of py48S-open complex (grey) would clash with eIF1 (cyan) of py48S-closed complex, highlighting the need for the conformational change within the TC during the open to closed PIC transition. The 40S, $tRNA_i$, mRNA and eIF1 of the py48S-closed complex are shown in color while only $tRNA_i$ and eIF2β of py48S-open complex are shown in grey.

(D) Superimposition of eIF2-βγ dimer of py48S- open (yellow) and closed (blue) complexes with the most similar archaeal βγ dimer (grey, from 3CW2) using γ as a reference shows different position of the β subunit with respect to the γ subunit.

(E) Relative position of TC in py48S-open (color) and py48S-closed (grey) based on superposition of the two complexes. The cartoon and surface representation show the relative position of each component of the TC.

A

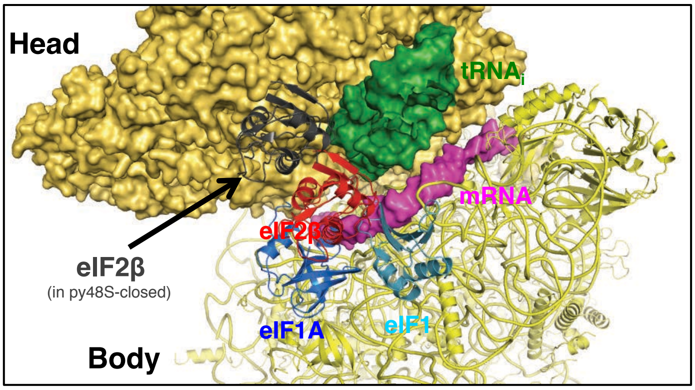

Head
tRNAi
mRNA
eIF2β
eIF2β
(in py48S-closed)
eIF1A
eIF1
Body

B

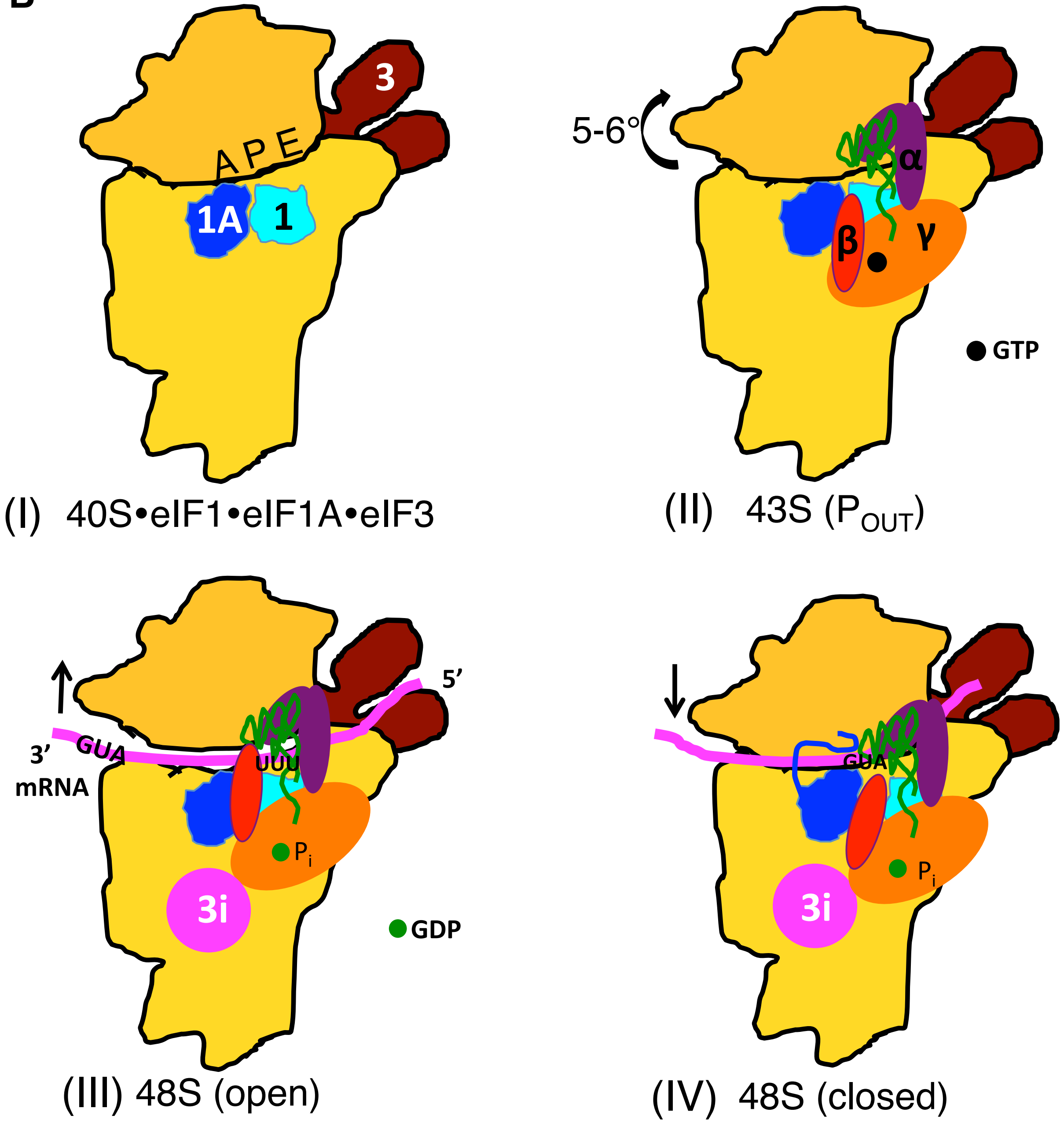

3
A P E
1A
1
(I) 40S•eIF1•eIF1A•eIF3
5-6°
α
β
γ
GTP
(II) 43S (POUT)
3'
mRNA
GUA
UUU
5'
Pi
3i
GDP
(III) 48S (open)
GUA
Pi
3i
(IV) 48S (closed)

Figure S7: Conformational changes from open to closed state, related to Figure 5

(A) Opening of the mRNA channel. Modeling of the position of eIF2β (grey) (with respect to 40S head and $tRNA_i$) observed in the py48S-closed complex into the py48S-open complex shows how in this conformation the mRNA channel would be opened up. In the py48S-open complex, eIF2β (red) blocks mRNA access by forming interactions with $tRNA_i$ (green) attached to head and eIF1 (cyan) and eIF1A (blue) attached to the body. The acceptor arm of $tRNA_i$ is not shown for clarity.

(B) Major structural changes during eukaryotic translation initiation. Binding of eIF1, eIF1A and eIF3 to the 40S subunit (I) facilitates TC binding in the P_{OUT} conformation to form the 43S PIC (II). Upward movement of the head expands the mRNA entry channel, allowing mRNA recruitment, and widens the P site to form the scanning-conducive py48S-open (III). eIF2β contacts eIF1 and probably stabilizes this open conformation while eIF3 undergoes major conformational change and eIF3i is repositioned on the subunit-interface. On AUG recognition, the head moves downward to clamp in the mRNA and enclose the $tRNA_i$ in the P_{IN} state of py48S-closed (IV). eIF2β loses contact with eIF1 and moves away. (See text for further details.)

Supplementary Table

Table S1. Local resolution, Related to Figure 1.

**Local resolution**	**py48S-open**	**py48S-closed**	**40S• eIF1• eIF1A**	**py48S calculated from EMD-2763** (Hussain et al., 2014)
Overall	6.00	4.90	3.45	4.00
Body of 40S	6.00	4.90	3.40	4.05
Head of 40S	6.30	5.05	3.55	4.10
eIF1	7.90	5.30	3.65	6.80
eIF1A	7.20	5.10	3.70	4.60
$tRNA_i$	7.90	5.50	-	5.90
eIF2α	8.75	6.25	-	6.85
eIF2β	9.15	7.15	-	-
eIF2γ	9.35	10.30	-	14.90
ASL of $tRNA_i$ and mRNA (-4 to +4)	7.30	4.80	-	4.70
eIF3-bgi subcomplex	10.30	8.30	-	-
eIF3c 5-helix bundle	7.05	5.70	-	-
eIF3 PCI domains	-	13.80	-	-

Supplementary Movie Legends

Movie S1: py48S-closed complex, related to Figure 1
This movie shows a 360° rotation of the map of the py48S-closed complex, followed by the fitting of refined coordinates in the map. The fitting of ligands can also be observed with the maps shown as a transparent surface. The β-propeller of eIF3b shown at the solvent interface is modeled based on previously reported structure (Aylett et al., 2015).

Movie S2: py48S-open complex, related to Figure 1
This movie shows a 360° rotation of the map of py48S-open complex, followed by the fitting of refined coordinates in the map. The fitting of ligands can also be observed with maps shown as a transparent surface.

Movie S3: Morphing of PICs: py48S-open to py48S-closed complex, related to Figure 2
This movie shows the morphing of 18S rRNA in the py48S-open to the py48S-closed complex (colored cyan in the first frame). A short region (1148-1163; 1615-1627) in helix h28 is shown in red. Most ligands (except $tRNA_i$ and eIF2α) and all ribosomal proteins have been removed for clarity. The front view shows the upward movement of the head while no major conformational change is observed in the body. The change in position of $tRNA_i$ and eIF2α with the head movement can be clearly seen.

Movie S4: Morphing of PICs: 40S•eIF1•eIF1A PIC to py48S-closed complex, related to Figure 2
This movie shows the morphing of 18S rRNA in the 40S•eIF1•eIF1A PIC to the py48S-closed complex (colored blue in the first frame). A short region (1148-1163; 1615-1627) in helix h28 is shown in red. All ligands and ribosomal proteins have been removed for clarity. The front view shows the rotation of the head while no major conformational change is observed in the body.

Movie S5: Morphing of ligands: py48S-open to py48S-closed complex, related to Figure 5

This movie shows the morphing of the 18S rRNA and ligands in the py48S-open to the py48S-closed complex. The ligands are shown in color, as in Figure 1. Only the eIF3i subunit is shown for eIF3. All ribosomal proteins have been removed for clarity. This movie shows the conformational change that TC undergoes during the transition from the open to the closed state.

Supplemental Experimental Procedures

Recombinant eIF3 production

In order to obtain an initiation complex in an open scanning-competent state, eIF5 was omitted in the preparation. Since eIF5 often copurifies with eIF3 in *S. cerevisiae* (Acker et al., 2007; Hussain et al., 2014), we overexpressed eIF3 in *Escherichia coli* as follows. The genes for subunits of eIF3 were cloned into two different but compatible polycistronic vectors: eIF3a and eIF3c were cloned into a pCDF Duet vector including an N-terminal his-tag for subunit a, and eIF3b, eIF3g and eIF3i in a pQlink vector. These two plasmids were used to transform *E. coli* Rosetta cells and the expression was carried out at 30 °C after induction with 0.5 mM IPTG. The protein was purified using the same steps used for the protein expressed in yeast (Mitchell et al., 2010). The protein obtained is soluble, pure and seems to be expressed in stoichiometric amounts (judged by SDS-PAGE). However visualization of the purified protein by SDS-PAGE shows that the eIF3c and the eIF3g subunits are slightly smaller than expected. Mass spectrometry analysis of these bands suggests that they may be missing part of their N-terminal region due to possible proteolysis (up to 42 N-terminal residues of its 812 amino acids for eIF3c and up to 65 N-terminal residues of its 274 amino acids for eIF3g). Nonetheless, the protein seems to be functional: it is able to interact with the other eIF3 subunit (subunit eIF3j), with the 40S ribosomal subunit, and it promotes mRNA recruitment using the assay described by (Mitchell et al., 2010), with a K_{extent} (defined as the concentration of eIF3 necessary for half maximal extent of mRNA recruitment) similar to that of eIF3 purified from yeast.

Reconstitution of 48S complexes

K. lactis 40S subunits were prepared as described earlier (Fernandez et al., 2014). *S. cerevisiae* eIF3 and eIF2 were expressed in yeast while eIF1, eIF1A and eIF5 were expressed in *E. coli* as recombinant proteins and purified as described (Acker et al., 2007). Recombinant eIF3 used for preparation of py48S-open complex was expressed in *E. coli.* Wild type $tRNA_i$ was expressed and purified from yeast and mutant $tRNA_i$ was transcribed and aminoacylated as described (Acker et al., 2007). Unstructured mRNAs with AUG (5′ GGAA$[UC]_4$U<u>AUG</u>$[CU]_4$C 3′) and AUC (5′ GGAA$[UC]_4$U<u>AUC</u>$[CU]_4$C 3′) codons were commercially synthesized by Integrated DNA Technologies and used for the py48S-closed and py48S-open complexes, respectively. Both complexes were reconstituted by incubating 120 nM 40S with eIF1, eIF1A, TC (consisting of eIF2, GDPCP and Met-$tRNA_i$), eIF3, eIF5 and mRNA in 40S:eIF1:eIF1A:TC:eIF3:eIF5:mRNA molar ratios of 1:2.5:2.5:1.5:1.2:2.5:2, with the exception that eIF5 was excluded in py48S-open, in 20 mM MES, pH 6.5, 40 mM potassium acetate, 10 mM ammonium acetate, 8mM magnesium acetate, 2mM dithiothreitol. The sample was used directly to make cryo-EM grids without further purification. Grids with sample for electron microscopy were prepared as described (Fernandez et al., 2014).

Analysis, structure determination, model building and refinement

Details of 3D classification

For py48S-closed complex data set

From about 5500 micrographs, a total of approximately 1,200,000 particles were picked. 2D class averaging was performed and aberrant particles were discarded. An initial reconstruction was made from all selected particles (1,182,309) after 2D class averaging using the yeast 40S crystal structure low pass filtered to 40 Å as an initial model. Next, a 3D classification into 8 classes with fine angular sampling was performed. Upon refinement only two classes were refined to high resolution: class 3 [12.6 %; 149,369 particles; 4.6 Å; PIC with TC] and class 4 [31.6 %; 374,737 particles; similar to PIC-2 (Hussain et al., 2014)]. The class 3, which showed a PIC with TC, was further classified into 3 classes: 3A [15,044 particles; 7.4 Å], 3B [112,924 particles; 4.3 Å; similar to py48S (Hussain et al., 2014)] and 3C [21,401 particles; 4.9 Å; py48S-closed complex].

For py48S-open complex data set

A data set of more than 2000 images was collected and about 500,000 particles were picked. An initial reconstruction was made from all selected particles (460,079) after 2D class averaging using the yeast 40S crystal structure low pass filtered to 40 Å as an initial model. Next, a 3D classification into 10 classes with fine angular sampling was performed. Class 1 showed the presence of a 40S dimer and was discarded. Class 2 showed the presence of TC. Classes 3-10 were not homogenous enough and showed the presence of at least eIF1A. Class 2 was subsequently divided into 5 classes: 2A, 2B, 2C, 2D and 2E. Class 2A was comprised of 97,864 particles and showed the presence of TC. It was again further divided into 3 classes: 2A-1, 2A-2 and 2A-3. Class 2A-1, comprised of 86,055 particles, consisted of the 40S•eIF1•eIF1A complex and was refined to a resolution of 3.5 Å. Class 2A-2 was comprised of 5,174 particles and shows a PIC containing TC but without eIF3, similar to py48S (Hussain et al., 2014), at about 7 Å resolution. Surprisingly this class does not seem to have density for eIF1. Class 2A-3 was not homogenous enough to be refined to moderate resolution. Hence we made a subset of 351,827 particles by combining Classes 3-8 from the first round of classification, Classes 2B-2E from the second round and Class 2A-3 from the third round. In other words, we left out Class 1 (which contained 40S dimers); Class 2A-1 (40S•eIF1•eIF1A) and Class 2A-2 (PIC with eIF1A and TC but without eIF3 and eIF1). This subset was then divided into 5 classes: A, B, C, D and E. Class A contained TC (70,365 particles) and it was then subsequently classified into 4 classes: A1, A2, A3 and A4. Only class A1 (6,127 particles; 6.1 Å) showed the presence of TC with eIF3. Class A1 was then further classified into 2 classes: A1-1 and A1-2. Class A1-1 (4,547 particles; 6.0 Å) represented the most complete class in this data set and contained 40S with eIF1, eIF1A, TC and eIF3, described here as the py48S-open complex, while Class A1-2 (1,580 particles; 14.9 Å) contains a PIC with eIF1, eIF1A and TC (without eIF3) corresponding to the previously reported pm43S complex (Hashem et al., 2013).

Detailed model building

Initially, the atomic coordinates of py48S (PDB: 3J81) were placed into the EM density of py48S-closed complex by rigid-body fitting using Chimera (Pettersen et al., 2004). For py48S-open, the body and head of the 40S of this same model were independently placed. Previously, 40S model had been further improved using the 40S•eIF1•eIF1A structure at 3.5 Å presented here. Then, each chain of the model (including ribosomal proteins, rRNA segments, protein factors and $tRNA_i$ and mRNA) was rigid-body fitted in Coot (Emsley et al., 2010) to overcome local differences in its positions. When necessary, also each separate domain of proteins was also subject to independent rigid body fitting, as was the case of factor eIF2α.

Most of eIF2β was not present in py48S (PDB: 3J81). In both open and closed complexes the relative orientation of eIF2β and eIF2γ is the same. There are three different published archaeal IF2 βγ (aIF2βγ) dimer crystal structures(Stolboushkina et al., 2008; Sokabe et al., 2006; Yatime et al., 2007), which substantially differ in the relative orientation of the two subunits. The most similar to ours correspond to PDB: 3CW2 (Stolboushkina et al., 2008), hence we superimposed aIF2βγ using our eIF2γ as a reference, and then we rigid-body fit the β-subunit independently. However, in this crystal structure used as a model, the ZBD is disordered. Therefore we used the ZBD in PDB: 2D74 to model it.

In py48S-open, wild type $tRNA_i$ was used from PDB: 1YFG for initial rigid-body fitting into its corresponding density and further improvement of the fitting was done with the morphing tool in Coot. Also in py48S-open NTT of eIF1A was removed from the model and eIF1 model was substituted by its counterpart in the 40S•eIF1•eIF1A structure. Finally, we observed a density close to the bases U and A from the anticodon of the $tRNA_i$. We reason this density most likely belongs to bases A and U from the mRNA and in consequence this fragment of mRNA was included in the final py48S-open model.

Model Building of eIF3

Fitting of eIF3a/eIF3c PCI dimer

Although the overall densities for eIF3 at the subunit interface were similar in the two structures, the higher-resolution py48S-closed map at 4.9 Å was used to generate a model for eIF3 bound in these initiation complexes. First the dimer of complete PCI domains of eIF3a and eIF3c was generated using the crystal structures of the full PCI domain of eIF3a (PDB: 4U1D) and the eIF3a/eIF3c PCI dimer (PDB: 4U1C) from *S. cerevisiae*. (Erzberger et al., 2014). This complete eIF3a/eIF3c PCI dimer was docked as a rigid body into the density on the solvent face of the 40S in py48S-close complex. Because of variation in the resolution of the eIF3 domains, we cannot resolve individual helices for the eIF3a/eIF3c PCI domains, however, it is possible to discern the overall shape and dimensions of PCI domains and do a rigid-body fit of the eIF3a/eIF3c PCI dimer. The fitting is similar to that of the eIF3a/eIF3c PCI heterodimers in the recent yeast 40S•eIF1•eIF1A•eIF3 structure (Aylett et al., 2015) and also to the PCI•MPN core model (PDB: 3J7K) docked into the pm43S EM map (Hashem et al., 2013).

Fitting of eIF3i and associated eIF3b-CTD and eIF3g-NTD

Density for a β-propeller domain was observed near h44 in the vicinity of eIF2γ. There are β-propeller domains in two subunits of eIF3: eIF3b and eIF3i. Crystal structures of both domains (PDB: 4U1E, 4U1F) from *S. cerevisiae* are now available (Erzberger et al., 2014). The nine-bladed β-propeller domain of eIF3b has been well documented to interact at the solvent (Liu et al., 2014; Erzberger et al., 2014) rather than the intersubunit face of the 40S as also observed in the yeast 40S•eIF1•eIF1A•eIF3 structure (Aylett et al., 2015). Moreover, the nine-bladed β-propeller domain of eIF3b is larger than the observed density near h44. In fact, rigid body fitting of the β-propeller domain of eIF3b shows a steric clash with 40S.

In contrast, the seven-bladed β-propeller domain of eIF3i fits well into the density. At the local resolution, it is not possible to discern its individual β-strands but the overall shape and dimensions guides the fitting of eIF3i. In one of the two crystal structures of eIF3i (PDB: 3ZWL; (Herrmannova et al., 2012)), there is a loop (residues 258-273) emanating from the β-propeller. A single mutation of a residue of this eIF3i loop confers a severe decrease of translation initiation without affecting the integrity of eIF3 (Cuchalova et al., 2010). We observe density for this loop in a slightly different conformation and interacting with h44. eIF3i also makes an interaction with a long helix at the C-terminus of eIF3b (PDB: 4U1E, 3ZWL). We observe density for this helix, interacting with the β-propeller domain further supporting that this density belongs to eIF3i and not eIF3b. Extra density was also observed for a portion of eIF3g-NTD in direct contact with this β-propeller domain that is consistent with the eIF3b-CTD/eIF3i/eIF3g-NTD trimeric crystal structure (PDB: 4U1E). These observations strongly suggest that the β-propeller domain is part of eIF3i. Moreover, its contacts with eIF3b and eIF3g make it possible to orient the β-propeller domain despite the fact that the individual blades cannot be resolved.

The C-terminal helix of this stretch of eIF3g-NTD points towards the entry channel, where the remainder of the protein (not resolved here) likely binds. Similarly, the N-terminal end of the eIF3b-CTD segment in the trimeric subcomplex points towards the likely position of the eIF3b β-propeller domain and these further supports the positioning of eIF3b-CTD/eIF3i/eIF3g-NTD trimer at the subunit interface near h44 in the vicinity of eIF2γ. As we observe density for only a portion of the eIF3bβ-propeller domain in py48S-close complex, the exact length of the connector between the two eIF3b domains (eIF3b β-propeller domain and eIF3b-CTD segment) resolved here cannot be specified. However, 39 residues in this connector are adequate to span the distance as an unstructured linker.

Fitting of helices of eIF3a and eIF3c at the intersubunit interface

The eIF3a/eIF3c PCI dimer and eIF3b-CTD/eIF3i/eIF3g-NTD trimer make up more than half of the eIF3 complex in *S. cerevisiae*. Apart from the density corresponding to the eIF3b-CTD/eIF3i/eIF3g-NTD trimer, we observe three additional and differentiated regions of densities at the intersubunit interface of the 40S. One of these corresponds to a group of 5 helices that is clearly recognizable at this resolution near

h21/h24/h27. More density is located in contact eIF1 on the platform and seems to correspond to a globular domain of around 70-100 residues. Finally, there is density for two very long helices (clearly recognizable at this resolution) arranged as a coiled coil spanning the beta propeller of eIF3i and the density on the platform near eIF1.

Having assigned both the eIF3a/eIF3c PCI dimer and the eIF3b-CTD/eIF3i/eIF3g-NTD trimer, the C-terminus of eIF3a (496-964), NTD and β-propeller domain of eIF3b, N-terminus of eIF3c (1-250) and eIF3g-CTD are not accounted for. eIF3b can be ruled out because both the NTD and β-propeller domains of eIF3b are expected to be at the solvent interface (Liu et al., 2014; Erzberger et al., 2014). In fact, although we do not observe a distinct density for the whole β-propeller domain of eIF3b, we observe extra density at low resolution for part of the eIF2β-propeller domain in py48S-close complex at its expected position (see Movie S1). The eIF3g-CTD is known to bind near the entry channel (Cuchalova et al., 2010) and a solution structure of human eIF3g-CTD is available (PDB: 2CQ0). Based on this available structural data and its known location on the 40S, the eIF3g-CTD was also ruled out. Therefore, these unassigned densities should mainly correspond to segments of eIF3a and eIF3c.

Secondary structure prediction for the N-terminus of eIF3c (1-250) suggests a region of about 100 residues containing 5-6 helices of various lengths and at least another isolated helix close to the N-terminus, whereas the C-terminus of eIF3a (496-964) is predicted to consist of very long helices. Thus for the remaining unassigned density, we reason that the 5 helices near h21/h24/h27 belong to the 120-220 region of eIF3c based on this secondary structure prediction. This region (residues 120-220 of eIF3c) is predicted to have a group of 5 helices and the observed density clearly corresponds to a group of helices. The length of helices observed in the density also corresponds to what is expected according to the secondary structure prediction for 120-220 region of eIF3c. We modeled individual helices into the density but were unable to determine its topology, as the connecting loops are not clear and there is no side-chain information for an unambiguously sequence assignment. However, we reason that these densities correspond to the helices in the region of residues120-220 of eIF3c. Crosslinking data indicate eIF3c interacts with uS15 (Erzberger et al., 2014) and these helices can be easily linked to the PCI domain present on the solvent interface by a linker (~30 residues), which may interact with uS15 (Figure 6D) further supporting this assignment.

Based on the volume of density (equivalent to a globular domain of around 70-100 amino acids) in contact with eIF1 on the platform and its relative proximity to group of helices near h21/h24/h27 (tentatively assigned to region of residues120-220 of eIF3c), we suggest that it belongs to the N-terminal end of eIF3c (residues ~1-90), where it would form a direct contact with eIF1, in agreement with previous studies describing the most extreme N-terminal part of eIF3c as an interacting partner of eIF1 (Reibarkh et al., 2008; Erzberger et al., 2014). However, the density we observe is not sufficiently detailed so as to enable any model building. So, the assignment of this density in contact with eIF1 to N-terminal end of eIF3c (residues ~1-90) is primarily based on biochemical studies indicating N-terminal end of eIF3c interacts with eIF1. Secondly, its proximity to region of residues120-220 of eIF3c further supports it. Thirdly, we have ruled out more or less the rest of eIF3.

The remaining density for two long kinked helices spanning the β-propeller domain of eIF3i and the proposed eIF3c-NTD density near eIF1 can therefore only belong to eIF3a, in agreement with its secondary prediction of long stretches of helices, most likely to its CTD (from residue 760). The density for the long helices is reasonably clear and thus we assign it to the CTD of eIF3a. In fact previous studies have suggested the existence of a spectrin domain (bundle of three long helices) at the CTD of eIF3a functioning as the docking site for the formation of the a:b:i:g subcomplex (Dong et al., 2013). A recent study suggested direct interaction of the CTD of eIF3a with eIF3i and with the NTD of eIF3c (Politis et al., 2015), in agreement with the model proposed here. These helices account for more than 100 residues, and therefore our assignment (from residue 760) places the extreme C-terminal ~100 residues of eIF3a not modeled here in the vicinity of the TC, consistent with a known eIF3a-CTD interaction with eIF2 (Valasek et al., 2002).

Model Refinement and Validation

For an optimal fitting of the models into the EM density maps we used REFMAC v5.8, which has been modified to work with EM maps in a wide range of resolutions (Amunts et al., 2014; Brown et al., 2015). For all ribosomal and protein factors, ProSMART (Nicholls et al., 2012) was used to generate idealized helical restraints and hydrogen bond restraints for β-sheets. Base pair and stacking restraints for rRNA, $tRNA_i$ and mRNAs were generated using the program LIBG (Brown et al., 2015). All restraints were maintained throughout refinement. Refinement with restraints helps to preserve the correct geometry of previously known structures as well as reduce overfitting. Therefore, in this work at the present resolutions, REFMAC essentially fixes the small clashes and geometry that occur after separate rigid body fitting of individual domains.
Average Fourier shell correlation (FSC) was monitored during refinement. The result of the refinements was checked visually in Coot (Emsley et al., 2010). Final model was validated using MolProbity (Chen et al., 2010). To prevent overfitting, the global refinement and external restraints weights were carefully adjusted by cross-validation, as previously described (Brown et al., 2015; Amunts et al., 2014). Refinement statistics are given in Table 1.

Yeast strain constructions

To generate strains LMY61, LMY74, and LMY76, strain KAY18 (*MATα leu2-3 leu2-112 ura3-53 ino1 sui3Δ gcn2Δ* p921 (*SUI3*$^+$*, URA3*)) (Asano et al., 1999) was transformed to Leu$^+$ with low-copy (lc) *LEU2* plasmids harboring FLAG-tagged alleles *SUI3-FL* (plasmid YCpSUI3), *sui3-FL-S202A,K214A* (pLEM13), or *sui3-FL-F217A,Q221A* (pLEM15), respectively, and the resident *SUI3*$^+$*,URA3* plasmid p921 was evicted by selection on 5-fluoorotic acid (5-FOA) medium. To generate strains ATY49, ATY53, and ATY54, strain JCY03 (*MATa ura3-52 leu2-3 leu2-112 trp1Δ-63 his4-301(ACG) sui1Δ::hisG* p1200 (*SUI1*$^+$, *URA3*)) (Cheung et al., 2007) was transformed to Leu$^+$ with single-copy *LEU2* plasmids harboring *SUI1*$^+$ (pJCB101), *sui1-F108A* (pAT117), or *sui1-F108D* (pAT118), respectively, and the resident *SUI1*$^+$ *URA3* plasmid (p1200) was evicted by selecting for growth on 5-FOA medium. Plasmids pLEM13 and pLEM15 were constructed from YCpSUI3 (Asano et al.,

1999) using the QuikChange site-directed mutagenesis system (Stratagene) according to the manufacturer's directions and the appropriate primers. Plasmids pAT117 and pAT118 were similarly constructed from pJCB101 (Martin-Marcos et al., 2011).

Yeast biochemical methods

Assays of β-galactosidase activities in whole-cell extracts (WCEs) were performed as described previously (Moehle and Hinnebusch, 1991) using transformants harboring the appropriate reporter plasmids, *HIS4*$_{AUG}$*-lacZ* (p367, *HIS4*$_{UUG}$*-lacZ* (p391) (Donahue and Cigan, 1988), or *GCN4-lacZ* (p180) (Hinnebusch, 1985). Transformants were cultured in appropriately supplemented synthetic dextrose minimal media (SD) at 30°C to an A_{600} of ~0.8. The same culture conditions were used for Western or coimmunoprecipitation analyses. WCEs for Western analysis were prepared by trichloroacetic acid extraction as previously described (Reid and Schatz, 1982), and immunoblot analysis was conducted as described (Nanda et al., 2009) using antibodies against Flag epitope (Sigma), eIF2Bε (Bushman et al., 1993), eIF1 (Valasek et al., 2004), or eIF3j (Valasek et al., 2001). Coimmunoprecipitations were conducted as previously described (Asano et al., 1999) and immunoblot analysis of immune complexes was conducted as above using antibodies against Flag epitope, eIF2γ (Hannig et al., 1993), eIF2α (Dever et al., 1995), and eIF2Bε.

SUPPLEMENTAL REFERENCES

Acker, M. G., Kolitz, S. E., Mitchell, S. F., Nanda, J. S., and Lorsch, J. R. (2007). Reconstitution of yeast translation initiation. Methods Enzymol *430*, 111-145.

Amunts, A., Brown, A., Bai, X. C., Llacer, J. L., Hussain, T., Emsley, P., Long, F., Murshudov, G., Scheres, S. H., and Ramakrishnan, V. (2014). Structure of the yeast mitochondrial large ribosomal subunit. Science *343*, 1485-1489.

Asano, K., Krishnamoorthy, T., Phan, L., Pavitt, G. D., and Hinnebusch, A. G. (1999). Conserved bipartite motifs in yeast eIF5 and eIF2Bepsilon, GTPase-activating and GDP-GTP exchange factors in translation initiation, mediate binding to their common substrate eIF2. EMBO J *18*, 1673-1688.

Aylett, C. H., Boehringer, D., Erzberger, J. P., Schaefer, T., and Ban, N. (2015). Structure of a Yeast 40S-eIF1-eIF1A-eIF3-eIF3j initiation complex. Nat Struct Mol Biol

Brown, A., Long, F., Nicholls, R. A., Toots, J., Emsley, P., and Murshudov, G. (2015). Tools for macromolecular model building and refinement into electron cryo-microscopy reconstructions. Acta Crystallogr D Biol Crystallogr *71*, 136-153.

Bushman, J. L., Foiani, M., Cigan, A. M., Paddon, C. J., and Hinnebusch, A. G. (1993). Guanine nucleotide exchange factor for eukaryotic translation initiation factor 2 in *Saccharomyces cerevisiae*: interactions between the essential subunits GCD2, GCD6, and GCD7 and the regulatory subunit GCN3. Mol Cell Biol *13*, 4618-4631.

Chen, V. B., Arendall, W. B., Headd, J. J., Keedy, D. A., Immormino, R. M., Kapral, G. J., Murray, L. W., Richardson, J. S., and Richardson, D. C. (2010). MolProbity:

all-atom structure validation for macromolecular crystallography. Acta Crystallogr D Biol Crystallogr *66*, 12-21.
Cheung, Y. N., Maag, D., Mitchell, S. F., Fekete, C. A., Algire, M. A., Takacs, J. E., Shirokikh, N., Pestova, T., Lorsch, J. R., and Hinnebusch, A. G. (2007). Dissociation of eIF1 from the 40S ribosomal subunit is a key step in start codon selection in vivo. Genes Dev *21*, 1217-1230.
Cuchalova, L., Kouba, T., Herrmannova, A., Danyi, I., Chiu, W. L., and Valasek, L. (2010). The RNA recognition motif of eukaryotic translation initiation factor 3g (eIF3g) is required for resumption of scanning of posttermination ribosomes for reinitiation on GCN4 and together with eIF3i stimulates linear scanning. Mol Cell Biol *30*, 4671-4686.
Dever, T. E., Yang, W., Astrom, S., Bystrom, A. S., and Hinnebusch, A. G. (1995). Modulation of tRNA(iMet), eIF-2, and eIF-2B expression shows that GCN4 translation is inversely coupled to the level of eIF-2.GTP.Met-tRNA(iMet) ternary complexes. Mol Cell Biol *15*, 6351-6363.
Donahue, T. F., and Cigan, A. M. (1988). Genetic selection for mutations that reduce or abolish ribosomal recognition of the HIS4 translational initiator region. Mol Cell Biol *8*, 2955-2963.
Dong, Z., Qi, J., Peng, H., Liu, J., and Zhang, J. T. (2013). Spectrin domain of eukaryotic initiation factor 3a is the docking site for formation of the a:b:i:g subcomplex. J Biol Chem *288*, 27951-27959.
Emsley, P., Lohkamp, B., Scott, W. G., and Cowtan, K. (2010). Features and development of Coot. Acta Crystallogr D Biol Crystallogr *66*, 486-501.
Erzberger, J. P., Stengel, F., Pellarin, R., Zhang, S., Schaefer, T., Aylett, C. H., Cimermancic, P., Boehringer, D., Sali, A., Aebersold, R., and Ban, N. (2014). Molecular architecture of the 40SeIF1eIF3 translation initiation complex. Cell *158*, 1123-1135.
Fernandez, I. S., Bai, X. C., Murshudov, G., Scheres, S. H., and Ramakrishnan, V. (2014). Initiation of translation by cricket paralysis virus IRES requires its translocation in the ribosome. Cell *157*, 823-831.
Hannig, E. M., Cigan, A. M., Freeman, B. A., and Kinzy, T. G. (1993). GCD11, a negative regulator of GCN4 expression, encodes the gamma subunit of eIF-2 in *Saccharomyces cerevisiae*. Mol Cell Biol *13*, 506-520.
Hashem, Y., des Georges, A., Dhote, V., Langlois, R., Liao, H. Y., Grassucci, R. A., Hellen, C. U., Pestova, T. V., and Frank, J. (2013). Structure of the mammalian ribosomal 43S preinitiation complex bound to the scanning factor DHX29. Cell *153*, 1108-1119.
Herrmannova, A., Daujotyte, D., Yang, J. C., Cuchalova, L., Gorrec, F., Wagner, S., Danyi, I., Lukavsky, P. J., and Valasek, L. S. (2012). Structural analysis of an eIF3 subcomplex reveals conserved interactions required for a stable and proper translation pre-initiation complex assembly. Nucleic Acids Res *40*, 2294-2311.
Hinnebusch, A. G. (1985). A hierarchy of trans-acting factors modulates translation of an activator of amino acid biosynthetic genes in *Saccharomyces cerevisiae*. Mol Cell Biol *5*, 2349-2360.
Hussain, T., Llacer, J. L., Fernandez, I. S., Munoz, A., Martin-Marcos, P., Savva, C. G., Lorsch, J. R., Hinnebusch, A. G., and Ramakrishnan, V. (2014). Structural changes enable start codon recognition by the eukaryotic translation initiation complex. Cell *159*, 597-607.

Liu, Y., Neumann, P., Kuhle, B., Monecke, T., Schell, S., Chari, A., and Ficner, R. (2014). Translation initiation factor eIF3b contains a nine-bladed beta-propeller and interacts with the 40S ribosomal subunit. Structure *22*, 923-930.
Martin-Marcos, P., Cheung, Y. N., and Hinnebusch, A. G. (2011). Functional elements in initiation factors 1, 1A, and 2beta discriminate against poor AUG context and non-AUG start codons. Mol Cell Biol *31*, 4814-4831.
Mitchell, S. F., Walker, S. E., Algire, M. A., Park, E. H., Hinnebusch, A. G., and Lorsch, J. R. (2010). The 5'-7-methylguanosine cap on eukaryotic mRNAs serves both to stimulate canonical translation initiation and to block an alternative pathway. Mol Cell *39*, 950-962.
Moehle, C. M., and Hinnebusch, A. G. (1991). Association of RAP1 binding sites with stringent control of ribosomal protein gene transcription in *Saccharomyces cerevisiae*. Mol Cell Biol *11*, 2723-2735.
Nanda, J. S., Cheung, Y. N., Takacs, J. E., Martin-Marcos, P., Saini, A. K., Hinnebusch, A. G., and Lorsch, J. R. (2009). eIF1 controls multiple steps in start codon recognition during eukaryotic translation initiation. J Mol Biol *394*, 268-285.
Nicholls, R. A., Long, F., and Murshudov, G. N. (2012). Low-resolution refinement tools in REFMAC5. Acta Crystallogr D Biol Crystallogr *68*, 404-417.
Pettersen, E. F., Goddard, T. D., Huang, C. C., Couch, G. S., Greenblatt, D. M., Meng, E. C., and Ferrin, T. E. (2004). UCSF Chimera--a visualization system for exploratory research and analysis. J Comput Chem *25*, 1605-1612.
Politis, A., Schmidt, C., Tjioe, E., Sandercock, A. M., Lasker, K., Gordiyenko, Y., Russel, D., Sali, A., and Robinson, C. V. (2015). Topological Models of Heteromeric Protein Assemblies from Mass Spectrometry: Application to the Yeast eIF3:eIF5 Complex. Chem Biol *22*, 117-128.
Reibarkh, M., Yamamoto, Y., Singh, C. R., del Rio, F., Fahmy, A., Lee, B., Luna, R. E., Ii, M., Wagner, G., and Asano, K. (2008). Eukaryotic initiation factor (eIF) 1 carries two distinct eIF5-binding faces important for multifactor assembly and AUG selection. J Biol Chem *283*, 1094-1103.
Reid, G. A., and Schatz, G. (1982). Import of proteins into mitochondria. Extramitochondrial pools and post-translational import of mitochondrial protein precursors in vivo. J Biol Chem *257*, 13062-13067.
Sokabe, M., Yao, M., Sakai, N., Toya, S., and Tanaka, I. (2006). Structure of archaeal translational initiation factor 2 betagamma-GDP reveals significant conformational change of the beta-subunit and switch 1 region. Proc Natl Acad Sci U S A *103*, 13016-13021.
Stolboushkina, E., Nikonov, S., Nikulin, A., Blasi, U., Manstein, D. J., Fedorov, R., Garber, M., and Nikonov, O. (2008). Crystal structure of the intact archaeal translation initiation factor 2 demonstrates very high conformational flexibility in the alpha- and beta-subunits. J Mol Biol *382*, 680-691.
Valasek, L., Nielsen, K. H., and Hinnebusch, A. G. (2002). Direct eIF2-eIF3 contact in the multifactor complex is important for translation initiation in vivo. EMBO J *21*, 5886-5898.
Valasek, L., Nielsen, K. H., Zhang, F., Fekete, C. A., and Hinnebusch, A. G. (2004). Interactions of eukaryotic translation initiation factor 3 (eIF3) subunit NIP1/c with eIF1 and eIF5 promote preinitiation complex assembly and regulate start codon selection. Mol Cell Biol *24*, 9437-9455.
Valasek, L., Phan, L., Schoenfeld, L. W., Valaskova, V., and Hinnebusch, A. G. (2001). Related eIF3 subunits TIF32 and HCR1 interact with an RNA recognition

motif in PRT1 required for eIF3 integrity and ribosome binding. EMBO J *20*, 891-904.
Yatime, L., Mechulam, Y., Blanquet, S., and Schmitt, E. (2007). Structure of an archaeal heterotrimeric initiation factor 2 reveals a nucleotide state between the GTP and the GDP states. Proc Natl Acad Sci U S A *104*, 18445-18450.
